# Deep neural networks with knockoff features identify nonlinear causal relations and estimate effect sizes in complex biological systems

**DOI:** 10.1093/gigascience/giad044

**Published:** 2023-07-03

**Authors:** Zhenjiang Fan, Kate F Kernan, Aditya Sriram, Panayiotis V Benos, Scott W Canna, Joseph A Carcillo, Soyeon Kim, Hyun Jung Park

**Affiliations:** Department of Computer Science, University of Pittsburgh, Pittsburgh, PA 15213, USA; Division of Pediatric Critical Care Medicine, Department of Critical Care Medicine, Children's Hospital of Pittsburgh, Center for Critical Care Nephrology and Clinical Research Investigation and Systems Modeling of Acute Illness Center, University of Pittsburgh, Pittsburgh, PA 15260,USA; Department of Human Genetics, University of Pittsburgh, Pittsburgh, PA 15213, USA; Department of Epidemiology, University of Florida, Gainesville, FL 32610, USA; Pediatric Rheumatology, The Children's Hospital of Philadelphia, Philadelphia, PA 19104, USA; Division of Pediatric Critical Care Medicine, Department of Critical Care Medicine, Children's Hospital of Pittsburgh, Center for Critical Care Nephrology and Clinical Research Investigation and Systems Modeling of Acute Illness Center, University of Pittsburgh, Pittsburgh, PA 15260,USA; Division of Pediatric Pulmonary Medicine, Children's Hospital of Pittsburgh, Pittsburgh, PA 15224, USA; Department of Pediatrics, School of Medicine, University of Pittsburgh, Pittsburgh, PA 15224, USA; Department of Human Genetics, University of Pittsburgh, Pittsburgh, PA 15213, USA

**Keywords:** causal inference, deep neural networks, effect size estimation

## Abstract

**Background:**

Learning the causal structure helps identify risk factors, disease mechanisms, and candidate therapeutics for complex diseases. However, although complex biological systems are characterized by nonlinear associations, existing bioinformatic methods of causal inference cannot identify the nonlinear relationships and estimate their effect size.

**Results:**

To overcome these limitations, we developed the first computational method that explicitly learns nonlinear causal relations and estimates the effect size using a deep neural network approach coupled with the knockoff framework, named causal directed acyclic graphs using deep learning variable selection (DAG-deepVASE). Using simulation data of diverse scenarios and identifying known and novel causal relations in molecular and clinical data of various diseases, we demonstrated that DAG-deepVASE consistently outperforms existing methods in identifying true and known causal relations. In the analyses, we also illustrate how identifying nonlinear causal relations and estimating their effect size help understand the complex disease pathobiology, which is not possible using other methods.

**Conclusions:**

With these advantages, the application of DAG-deepVASE can help identify driver genes and therapeutic agents in biomedical studies and clinical trials.

## Background

Since molecular and clinical variables interact for the development of complex diseases such as cancer, asthma, and sepsis [1–3], learning the causal structure among the variables helps identify risk factors, disease mechanisms, and candidate therapeutics for the complex diseases for future evaluation. For example, if an abnormal expression of a certain gene modifies the expression level of other genes and contributes to the development of a disease, then controlling this gene can lead to the effective treatment of the disease.

A popular statistical model for causal inference is the causal directed acyclic graph (DAG), which learns conditional dependence among variables [4–10] because the conditional dependence can further imply the causal relationships under 3 causal assumptions: Markov, faithfulness, and sufficiency. The causal Markov condition states that causal relationships among the set of variables in their probability distributions (e.g., Bayesian network) are conditionally independent of their nondescendants given their parents [11]. The causal faithfulness condition states that all independence relations in the data are consequences of the causal Markov condition. The causal sufficiency condition states that input data measured all the common causes of the measured variables, and thus no latent (unobserved) confounder exists. Since the assumptions are not usually met in data, statistical causal inference is limited to identifying causal relationships that are Markov equivalent, which hold the same adjacencies and imply the same independence and conditional independence relationships on the same variables (v-structure). Under the assumptions, bioinformatic methods have incorporated 2 main approaches to building DAGs: constraint based or score based [12–19]. Constraint-based algorithms learn constraints that restrict the set of possible causal graphs by testing conditional independence in the input data. Peter and Clark (PC) [12], one of the most popular algorithms under this category [20–25], uses a combination of conditional independence tests and graph-pruning techniques first to determine a skeleton of the DAG and then to determine the causal directions in the skeleton network. On the other hand, score-based algorithms generally formulate the causal learning problem as a search problem to optimize a certain score function with respect to an unknown DAG and the input data. For example, the degenerate Gaussian score (DG) was recently proposed [26] by extending the widely used Bayesian Information Criteria (BIC) score [13, 27] for mixed types of data. Specifically, by embedding discrete variables into a continuous space using one-hot vector representations, DG demonstrates a near-perfect performance under certain simulation scenarios of high-dimensional data.

Previously, causal inference methods have been successfully used to provide insights into molecular mechanisms and predict treatment effects. First, to provide insights into molecular mechanisms (e.g., transcriptional regulatory relationship between genes), methods have been developed to integrate multiple types of data where the direction of effect is known from one type to another (e.g., from DNA variants to gene expression). Developments in this approach utilized both score-based [28, 29] and constraint-based [30–33] algorithms. For example, a PC algorithm with the principle of Mendelian Randomization (MRPC) uses PC to examine a set of causal relationships between DNA variants and gene expression information implied by the principle of Mendelian randomization. Second, to predict treatment effects, causal inference was done using multiple intervention trial or experiment data (e.g., RNA interference–based gene knockout experiments). For example, conservative local causal discovery tests conditional (in)dependence among multiple entities (e.g., proteins) across experiments [34] and BACKSHIFT evaluates particular causal scenarios shared across experiments using a linear causal model [35]. Treatment effect can also be predicted based on the relationship of the input samples with other samples for which treatment effects are known. To this end, the causal k-nearest neighbor algorithm estimates the effect based on the nearest neighbors with known treatment effects. Similarly, causal random forest attempts to identify neighbors after recursively partitioning the covariate space through creating a set of decision trees. While they successfully identified biologically meaningful or clinically reasonable causal relations in various validation experiments, they are not necessarily relying on artificial intelligence (AI) methods and tend to test independence for a limited number of entities or based on naive assumptions on the relationships among data points.

Recently, methods have employed AI methods to address the limitations of previous causal inference methods. For example, a recent development, causal mixed graphical model (causalMGM) [36], first identifies associations between different types of data using a mixed graphical model (MGM) and then infers causality of the associations through PC. This 2-stage approach showed good scalability and accuracy for high-dimensional simulated and biological data of mixed types [36]. Also, to identify an optimal DAG based on an optimality score, a challenge can be the intractable search space that increases with a complexity super exponential to the number of the input variables. Thus, a group of methods has been developed to efficiently navigate the search space. Previously, this problem was addressed with additional structure assumptions, for example, in terms of tree width [37], number of variables [38], ancestral constraints [39], or a set of prior knowledge [40]. While they were designed to shrink the intractable search space with the assumptions, methods can also be developed to expand the search space and efficiently navigate it. In that regard, a recent breakthrough formulates the problem as a continuous optimization with a structural constraint that ensures acyclicity [41] and spurs further development of deep neural network (DNN) models. For example, Yu et al.[42] proposed a deep generative model and applied a variant of the structural constraint to learn the DAG (DAG-GNN), and Zheng et al. [43] generalized this framework so various approximations can be used for search (NOTEARS), including neural networks. Despite all substantial progresses in those approaches, we found several challenges to identify causality for complex diseases. First, a method should identify both linear and nonlinear associations. While linear associations may exist, complex biological systems are characterized by nonlinear associations [44, 45]. For example, the effects of hormone receptor status on breast cancer biology are often nonlinear due to their complex interactions with other molecular complexes in multiple regulation processes [46–48]. Some of the nonlinear associations may be revealed in the existing DNN methods. However, the methods utilize the DNN component to effectively navigate the search space over various DAGs while optimizing an optimality score across all the relationships in a DAG as has been done previously. Generally, the optimality scores are based on a likelihood model with product terms to represent the variable relationships. For example, as the optimality score, both DAG-GNN and NOTEARS can use the BIC score that selects the product terms to determine significant variable relationships. A product term of 2 variables assumes that the relationship between the variables is additive and proportional, meaning that the effect of one variable on the other is assumed to be constant across all levels of the other variable. Since the constant effect is satisfied only in linear relationships, the methods based on such optimality scores are designed to consider only linear relationships. In other words, as a DNN component is to address nonlinearity, existing methods use the DNN component to address nonlinearity in how various DAGs are searched through, not to address nonlinearity in each relationship. In this sense, they do not explicitly identify each causal relationship as nonlinear. Second, a method should estimate the effect size of each association. This is critical to facilitating a translatable understanding of the causal relationships since it is important to select a limited number of the most significant causal relationships for downstream experiments or clinical trials due to both technical and practical limitations. However, currently, no method can not only identify the nonlinear relationships but also estimate their effect size.

To address these limitations and enable a more realistic and translatable causal structure learning for complex diseases, we developed the first computational method that explicitly learns nonlinear causal relationships as well as linear causal relationships, named causal directed acyclic graphs using deep learning variable selection (DAG-deepVASE). To identify nonlinear causal relationships in high-dimensional data, DAG-deepVASE incorporated a 2-step approach: (i) identify associations and estimate their effect sizes and (ii) infer the causality among the associations. In the first step, to identify each causal relationship as nonlinear, DAG-deepVASE puts a DNN model between each potential causal relationship. However, a regular DNN model cannot estimate the effect size between an input variable and the response variable since it would be difficult to summarize the edge weights between neurons across multiple layers between the variables. To address this difficulty, DAG-deepVASE incorporated the knockoff framework into the DNN model to estimate the effect size. Previously, this architecture was used to control the false-positive rate in the context of variable selection [49]. In this work, we extend this architecture to measure the effect size in the context of causal inference for the first time. Further, to learn the causal direction for the identified nonlinear associations, DAG-deepVASE extends a score-based approach, DG. While it was not known which causal inference approach would learn the causal direction of nonlinear associations, we conducted extensive studies to find that its asymptotic properties make the inference tractable and flexible enough to learn nonlinear causalities.

DAG-deepVASE consistently outperforms other methods in identifying true causal relations in simulation data of diverse scenarios and identifying known and novel causal relations in molecular and clinical data of various diseases (pediatric sepsis, gut bacteria/nutrient intake and body mass index [BMI], and breast cancer), facilitating a systematic understanding of the complex disease pathobiology. In the analyses, we also illustrate how identifying nonlinear causal relations and estimating their effect size help understand the complex disease pathobiology, which is not possible using other methods.

## Findings

### DAG-deepVASE

**Figure 1:**
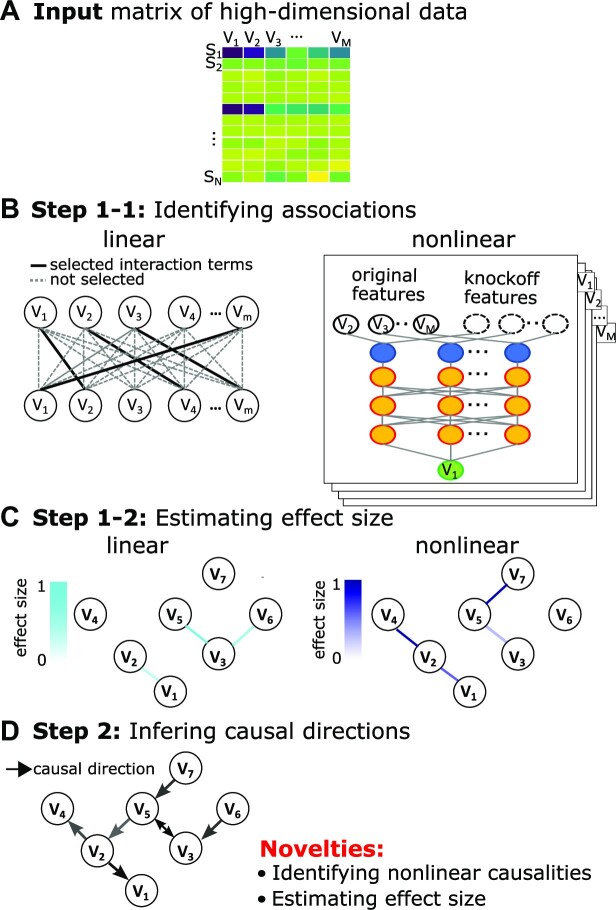
Overview of DAG-deepVASE. (A) An input data matrix consisting of *M* variables (V_1_, V_2_, …, V_M_), either continuous or ordinal categorical, collected from *N* samples. (B) Left: An example of the identified linear associations using a statistical graphical model (MGM). Right: Identifying nonlinear associations using a deep neural network (deep learning) model. After the first run sets V_1_ as response and identifies its association with other variables, DAG-deepVASE will run this model with each of the other variables (V_2_, V_3_, …, V_M_) as response and with all the other variables as input. (C) Left: Estimating the effect size of linear associations in the statistical graphical model. Right: Estimating the effect size of nonlinear associations in reference to the knockoff filter implemented in the deep learning model. (D) Learning the causalities by running the DG separately on the identified associations, either linear or nonlinear.

We provide here a brief overview of DAG-deepVASE that aims to identify linearly and nonlinearly associated variables while estimating their effect sizes (Fig. 1B, C, respectively) and learn their causal directions (Fig. 1D) to produce a DAG from data matrix *X* consisting of *M* input variables (Fig. 1A). In the first step, to identify linearly associated variables, DAG-deepVASE develops a penalized regression function with the interaction terms connecting the variables and maximizes the likelihood score with sparsity penalties (Methods, Fig. 1B). While the linear associations have been the main focus of previous causal inference methods [5], DAG-deepVASE further identifies nonlinearly associated variables by developing a set of DNN models, each with one of the input variables as the outcome and all the others as the dependent variables of the model (Fig. 1B). Note that this approach is different from most existing DNN-based causal inference methods in that DAG-deepVASE models nonlinearity in individual variable relationships while other methods model nonlinearity in the way variable relationships are combined with respect to the input data. Further, we set out to estimate the effect size on the individual variable relationships in our DNN model. Although estimating the effect size is important to design further clinical trials and/or experimental validations with strong drivers, it is not straightforward to summarize the edge weights across multiple layers for effect size estimation in a regular DNN approach. DAG-deepVASE successfully estimates the effect size of the nonlinear associations by embedding the knockoff variables in the DNN model (Fig. 1B). Knockoff variables are a synthetic and noisy copy of the input variables, which resemble the correlation structure of the input variables but are conditionally independent of the outcome, given the input variables. This property of knockoff variables allows us to estimate how important the original association is in reference to the knockoff variables, leading to the effect size estimation.

In the second step, after identifying both linear and nonlinear associations, DAG-deepVASE determines their causal direction using a single metric to ensure causal inference consistency between linear and nonlinear causalities. Since this is one of the first methods that identify nonlinear causal directions, it is unknown whether PC or DG would work better to identify nonlinear causal directions. Among various measures, we chose to use DG because it is accurate, decomposable, and flexible. While its accuracy, which was demonstrated in simulations [26], is clearly beneficial to learning accurate causal directions, we separately conducted an extensive study to find that its decomposability and flexibility were critical to identifying nonlinear causal directions. DG decomposes the task of identifying the optimal causal structure into determining the causal direction of each association. Whereas PC determines the optimal causal structure by considering all associations simultaneously, decomposability allows us to determine the causal direction of each nonlinear association without referring to other associations, making each causal inference tractable. DG also shows flexibility in learning the causal structure generated outside of its model class (conditional Gaussian model). This flexibility allows us to extend DG to learn nonlinear causal directions. In simulation data of diverse scenarios and biological data of various contexts, we demonstrate that DAG-deepVASE can learn causal relations up to the Markov equivalence classes of the true causal relationship.

### DAG-deepVASE improves power in identifying nonlinear causal relations in simulation data

To evaluate the performance of DAG-deepVASE in the presence of multiple causal variables, we compared DAG-deepVASE with competing methods on simulation data. Such methods include causalMGM, DG, NOTEARS, and DAG-GNN. We included causalMGM and DG because they employ the same 2-step strategy as DAG-deepVASE: identifying variable associations and then learning the causal direction of the associations. While causalMGM was originally developed with the 2-step strategy, DG does not have the first step because DG is developed to learn causality based on given associations. To be fair to DG, we developed the first step for DG: in the first step, we applied MGM to identify associations, and in the second step, we used the original DG to learn their causalities. We will refer to this model as the linear DG model since the MGM implementation identifies variable associations based on the linear interaction terms. Also, note that whereas DAG-deepVASE identifies both linear and nonlinear associations and uses DG to learn their causal directions, linear DG identifies linear associations and uses DG to learn their causal directions, and causalMGM identifies only linear associations and applies PC to learn their causal directions. We included NOTEARS and DAG-GNN because they are established DNN methods to infer causality. We ran the methods using default parameters or those suggested by the authors throughout this article (Table 1).

**Table 1:** Parameter settings for the deep learning component of DAG-deepVASE

	Parameter	Value
DNN	Activation function	Rectified linear unit (ReLU)
	Initial weight values	Glorot normal intializer
	Regularization	L1-regularization
	Optimization	Adam optimization
	Loss function	Mean squared error (MSE)
FDR	FDR control rate	0.05

**Figure 2:**
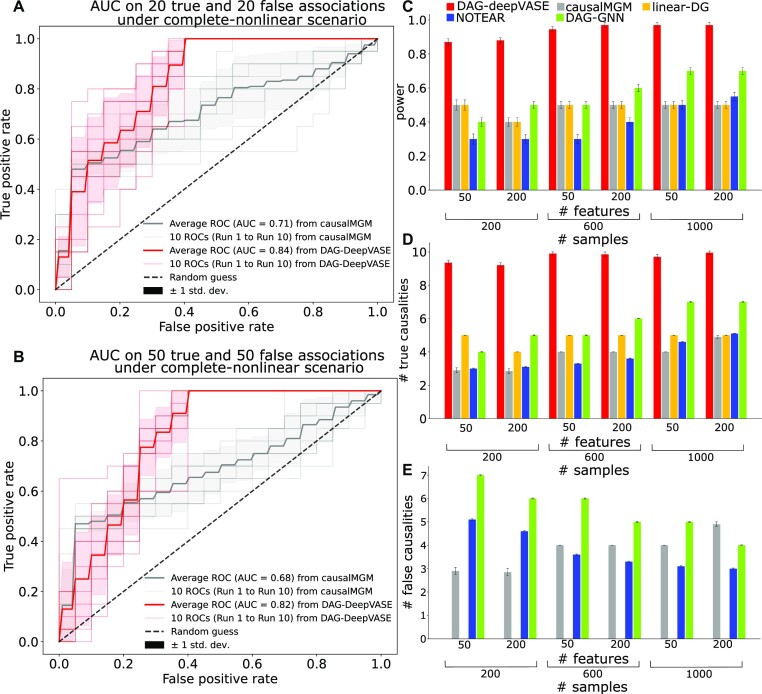
Performance assessment of causal inference methods on the simulated data AUC estimated for DAG-deepVASE and causalMGM on (A) 20 true and false associations and (B) 40 true and false associations, both under the complete-nonlinear scenarios. (C) Average number and standard error (error bar) of true associations in the complete-nonlinear scenario identified by DAG-deepVASE (red), causalMGM (gray), linear DG (yellow), NOTEAR (blue), and DAG-GNN (green) over 50 runs in various simulation scenarios, varying the number of features and sample sizes. Average number and standard error (error bar) of (D) true causalities and (E) false causalities. DAG-deepVASE and linear DG did not identify any false causalities.

To compare the methods in sensitivity and specificity simultaneously, we simulated 10 datasets of 40 or 100 variables where half (20 or 50, respectively) of the variables collectively determine the outcome (true associations) and the other half are not associated with the outcome (false associations; see Methods). Each dataset was simulated for 10,000 samples. To mimic biological variables that would interact in various degrees of nonlinearity, simulations were conducted under 2 scenarios: complete-nonlinear or partial-nonlinear scenarios. We ran DAG-deepVASE and causalMGM on the datasets. We did not run linear DG since it identifies the same association pairs as causalMGM. We did not run NOTEARS and DAG-GNN for this experiment since it is not straightforward to vary threshold values for plotting the receiver operating characteristic curve in the DNN architecture. In both complete- and partial-nonlinear scenarios, DAG-deepVASE consistently outperformed causalMGM in AUC area under the receiver operating characteristic curve (AUC). Specifically, for the simulations with 40 and 100 associations under the complete-nonlinear scenario, DAG-deepVASE achieves an average of 0.84 and 0.82 AUC, respectively, outperforming causalMGM, which achieves an average of 0.71 and 0.68 AUC (Fig. 2A and Fig. 2B, respectively). The same trend is observed under the partial-nonlinear scenario where DAG-deepVASE achieves an average of 0.84 and 0.83 AUC and causalMGM achieves an average of 0.73 and 0.71 AUC for the simulations with 40 and 100 associations (Supplementary Fig. S2A and Supplementary Fig. S2B, respectively).

To further mimic biological situations where true associations would be relatively rare among all pairwise combinations of biological variables, we simulated different numbers of variables (M = 50, 100, 200, 400, 600, 800, 1,000, 1,500, 2,000, 2,500, and 3,000) with various sample sizes (*n* = 200, 600, 1,000), where 10 variables collectively determine the outcome (true associations). For each combination of variable number and sample size, we conducted the simulation experiment 50 times. In the complete-nonlinear simulation scenario, we first compared the number of true associations identified by each method before assessing the causal directions. DAG-deepVASE shows a 2-fold higher power than the other methods by identifying more than 90% of the true associations in most simulation scenarios (Fig. 2C). Interestingly, while DAG-GNN performs slightly better than linear approaches, causalMGM and linear DG, in terms of power and sensitivity, NOTEARS performs the worst in all scenarios in general. Second, we compared the number of true and false causal directions learned from the identified associations (Fig. 2D, respectively, Supplementary Table S1). In all experiments under the complete-nonlinear scenario, DAG-deepVASE consistently outperforms the other methods in identifying true causalities. Especially for larger sample sizes (*n* = 600 and 1,000), DAG-deepVASE identified more than 97% of the true causalities. causalMGM returned bidirectional causal directions for all identified associations, which are counted as both true and false positives. On the other hand, although linear DG identified less than half of the true associations as mentioned above, it learned the true causalities on the small number of the identified associations (Fig. 2D), demonstrating that DG can be used to learn nonlinear causalities. Further, the other DNN methods also identified less than 50% of the true causalities than DAG-deepVASE. Together with such high true-positive rates, DAG-deepVASE also outperforms the other methods by not identifying any false casualties in any of the scenarios, whereas competing methods suffer from high false causalities. For example, causalMGM returns 3 to 5 false-positive causalities by returning bidirectional causalities (Fig. 2E, Supplementary Fig. S1D), and both DNN methods, NOTEARS and DAG-GNN, suffer from the highest number of false causalities. In the partial-nonlinear scenario, a very similar result is returned for power (Supplementary Fig. S1C), true-positive causalities (Supplementary Fig. S1D), and false-positive causalities (Supplementary Fig. S1E).

Altogether, DAG-deepVASE outperforms the other methods by identifying the highest number of true nonlinear associations and by learning the highest number of true causalities across various simulation scenarios without false positives, while competing methods could identify less than half of true nonlinear causalities with several false positives.

### DAG-deepVASE identifies both linear and nonlinear associations among clinical features with high sensitivity in pediatric sepsis data

**Figure 3:**
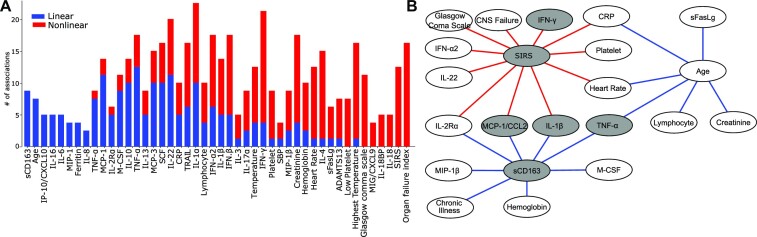
Linear and nonlinear associations in pediatric sepsis data. (A) Number of linear (blue) and nonlinear (red) associations involving each of the 45 variables. (B) A subnetwork of linear (blue) or nonlinear (red) variable associations involving SIRS (associated only nonlinearly) and sCD183 (associated only linearly) and with normalized effect size. Gray nodes connect between IFN-γ and TNF-α. For full names of the variables, readers are referred to Methods.

To demonstrate the importance of identifying nonlinear variable associations for sensitive causal inference, we first focus on identifying associations among diverse types of variables in clinical data. The data consist of clinical and biomarker variables (laboratory parameters, cytokines, and chemokine measurements) from 404 children with severe sepsis [50]. We compared DAG-deepVASE and causalMGM in this section. We excluded linear DG because they identify the same set of associations with causalMGM. We excluded NOTEAR and DAG-GNN from further analyses since they identified high rates of false-positive causalities in simulation studies. Since DAG-deepVASE assumes that the variables follow the Gaussian distribution, we consider 45 continuous or ordinal categorical variables excluding 1 binary/nominal variable in the dataset. Among the variables, DAG-deepVASE identifies 118 associations (Fig. 3A), whereas causalMGM identifies 42 associations (49.5%; Supplementary Table S2) of the associations. Since causalMGM is only able to identify linear associations, the 42 associations are likely linear. Many of the identified linear associations are already clinically and biologically verified. For example, the serum level of soluble CD163 (sCD163), a macrophage activator [51], only has linear associations (Fig. 3B) with biomarkers known to activate macrophages, such as macrophage colony-stimulating factor (M-CSF) [52], monocyte chemoattractant protein 1 (MCP-1) [53], interleukin (IL)–1b [54], tumor necrosis factor α (TNF-α) [55,56] and other key drivers of macrophage response, including its ligand hemoglobin [57]. Also, age is another variable only linearly associated with other variables, including heart rate, creatinine, and lymphocyte count (Fig. 3B). Since each of them changes monotonically with age in pediatric subjects [58–60], it is reasonable that they are identified as linear associations.

In addition to the 42 linear associations that are identified by both DAG-deepVASE and causalMGM, DAG-deepVASE uniquely identifies 76 nonlinear associations. Multiple nonlinear associations have been validated in previous clinical and biological studies with an implication for nonlinearity. An example is an association between systemic inflammatory response syndrome (SIRS) status and heart rate (Fig. 3B). This association is expected to be nonlinear, as the SIRS status is diagnosed by a nonlinear combination, which is the presence of any 2 of the 4 clinical criteria, including tachycardia (elevated heart rate) [72]. Also, as the SIRS response is defined as a result of systemic immunological activation, DAG-deepVASE uniquely found nonlinear associations between SIRS status and proinflammatory cytokines, including C-reactive protein (CRP) [73, 74], IL-1β [75], and interferon γ (IFN-γ) [76] (Fig. 3B), corroborating their collective roles in inflammation. Since cytokines are produced involving different combinations of signal transduction pathways [77, 78], their associations with SIRS are expected to be nonlinear rather than linear.

While the method identified validated associations, DAG-deepVASE also identified novel nonlinear relationships of clinical potential for future validation. For example, it identified the nonlinear associations between central nervous system (CNS) dysfunction and SIRS and between IL-22 and SIRS (Fig. 3B). The former is validated: critically ill patients with SIRS are known to have a measurable risk for organ dysfunction such as CNS dysfunction [79, 80]. This validation also confirms our causal inference that found the causal direction from SIRS status to CNS dysfunction (Supplementary Table S2). As it is imperative to elucidate how SIRS interacts with modifiable cytokines for clinical potential, our causal inference also suggests the novel clinical potential of IL-22 to treat CNS dysfunction through modifying SIRS status. While IL-22 plays a key role in immunoregulation and has been linked to the development of organ failure in mouse models of abdominal sepsis [81], DAG-deepVASE revealed the causal relationship from IL-22 to SIRS status in children with sepsis. Especially, it identified this relationship by strong effect size (top 19th out of 118; Supplementary Table S2), suggesting a strong reproducibility and thus clinical utility. After more experimental validations, this result can help design future clinical trials to treat organ dysfunctions with IL-22 for pediatric sepsis. Altogether, DAG-deepVASE identifies both validated and novel findings by identifying linear and nonlinear associations with high sensitivity.

### DAG-deepVASE accurately identifies nonlinear causalities and estimates their effect sizes in the nutrients/gut bacteria and BMI data

**Figure 4:**
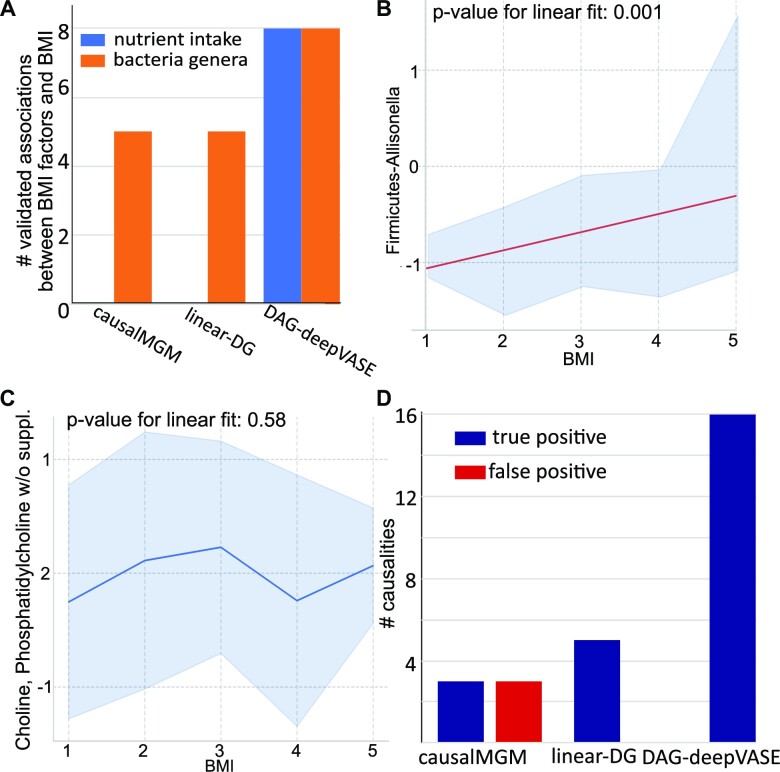
Performance assessment of 4 causal inference methods on various degrees of nonlinear associations in BMI/bacteria/gut microbiome data. (A) Number of associations the methods (causalMGM, linear DG, and DAG-deepVASE) identified between the BMI status and 8 nutrient intake (blue) and 8 bacteria genera in the gut (red) that are validated associated with the BMI status. (B) The relationship between BMI and Firmicutes–*Allisonella* identified with confidence interval (gray intervals). Red line represents the estimated linear regression, and *P* value for linear fit is calculated from a permutation test with *R*^2^ (Methods). (C) The relationship between BMI and choline, phosphatidylcholine w/o suppl. identified with confidence interval (gray intervals). Blue line connects the middle point of the BMI values 1 to 5. (D) Number of true-positive (dark blue) and false-positive (red) causalities identified by causalMGM, linear DG, and DAG-deepVASE. DAG-deepVASE and linear DG did not identify any false causalities.

Variables in complex biological systems interact in varying degrees of nonlinearity [82–84]. To examine the sensitivity of DAG-deepVASE in the presence of various degrees of nonlinearity, we compared DAG-deepVASE with causalMGM and linear DG on a cross-sectional dataset consisting of 214 nutrient intakes, 87 gastrointestinal (GI) bacteria genera, and BMI collected from 90 healthy volunteers [85]. Note that nutrient intakes would affect GI bacteria before affecting BMI, suggesting generally a more nonlinear relationship between the nutrient intakes and BMI than between them and GI bacteria. For a balanced assessment, we selected the same number (8) of nutrient intakes and bacteria genera that are known to affect BMI in animal experiments or clinical trials out of the 214 nutrient intakes and 87 bacteria genera data (Table 2). We selected the 16 features also because they were previously suggested to have nonlinear associations with BMI by a DNN-based variable selection method [49]. DAG-deepVASE identified 15 associations, while causalMGM and linear DG identified only 5 associations (31.3%): all these 5 associations are between specific GI bacteria and BMI (Fig. 4A). Note that causalMGM and linear DG failed to identify any association between nutrient intakes and BMI, while DAG-deepVASE could identify all 8 of them. Since nutrient intakes likely affect BMI more nonlinearly than between GI bacteria and BMI, this result reaffirms that DAG-deepVASE uniquely identifies nonlinear relationships. To characterize the nonlinear associations, we examined how the 8 nutrient intake and 8 bacteria genera levels change against the BMI value. The 5 associations between GI bacteria and BMI identified by all 3 methods show a single linear association throughout the BMI region (Fig. 4B [*P* -value for linear fit: 0.001], Supplementary Figure S2K–O). On the other hand, the other 8 associations between nutrient intakes and BMI and 3 associations between GI bacteria and BMI, which are identified by only DAG-deepVASE, show nonlinear relationships (Fig. 4C [*P* value for linear fit: 0.58], Supplementary Figure S2A–J [*P* value for linear fit on average: 0.42]), characterized by multiple subtrends across the BMI ranges. For example, choline and phosphatidylcholine w/o suppl. intake (Fig. 4C) shows an increasing trend from BMI 1 to 3, a decreasing trend from BMI 3 to 4, and then another increasing trend from BMI 4 to 5.

**Table 2:** Sixteen nonlinear associations (8 nutrient intakes and 8 bacteria genera) that were validated in literature

	Nutrient intake		Bacteria genera		
	Micronutrient	Reference	Phylum	Genus	Reference
1	Linoleic	[61]	Proteobacteria	*Sutterella*	[62]
2	Omega 6	[63]	Firmicutes	*Allisonella*	[64]
3	Dairy protein	[65]	Firmicutes	*Holdemania*	[66]
4	Aspartic acid, aspartame	[67]	Firmicutes	*Mitsuokella*	[68]
5	Phenylalanine, aspartame	[67]	Firmicutes	*Clostridium*	[64]
6	Choline, phosphatidylcholine	[69]	Firmicutes	*Megamonas*	[70]
7	Theaflavin 3-gallate, flavan-3-ol(2)	[71]	Firmicutes	*Megasphaera*	[68]
8	Choline, phosphatidylcholine w/o suppl.	[69]	Firmicutes	*Acidaminococcus*	[64]

In the second step of determining causalities from the identified associations, we deemed true the causal directions from nutrient intake/bacteria genera to BMI based on literature in Table 1. DAG-deepVASE identified true causal directions from all the 15 associations it found. On the other hand, as causalMGM uses PC to learn the causal directions of the associations, PC removed 2 of the 5 associations in its step of testing the conditional independence relationship and identified the other 3 associations as bidirectional causalities that we considered to be both false positive and false negative (Fig. 4D). While linear DG identified 5 true causal directions on the 5 identified associations, it still could not learn 11 causalities because of its inability to identify nonlinear causality. Altogether, DAG-deepVASE outperforms other methods due to its ability to identify nonlinear associations combined with the excellent performance of DG in learning nonlinear causalities among the identified associations.

Further, to demonstrate how the effect size DAG-deepVASE estimates lead to the unbiased discovery of causal relations, we estimated the effect size of all 301 potential associations between nutrient intake/bacteria genera and BMI using DAG-deepVASE, including nonvalidated ones (Supplementary Table S3). The top 5 associations that have the largest effect sizes estimated by DAG-deepVASE's nonlinear module are all validated: *Meganomas* [70], Phenylalanine [67], *Mitsuokella* [68], *Parvimonas* [67], and *Sporobacter* [68]. Since these findings were independent, it is difficult to prioritize their importance. To conduct further experimental validations or clinical trials that target a limited number of strong associations, estimating effect sizes via DAG-deepVASE enables to prioritize important variables. Also, while the 5 top features are already validated, it would be interesting to validate other novel features with large effect sizes.

### DAG-deepVASE identifies causal relations among molecular and clinical variables in breast cancer data

Among various types of variable interactions in a complex disease, identifying causal relationships between molecular variables (e.g., gene expression) and clinical variables (e.g., cytokine measurements in the serum) is particularly interesting because the findings can help identify molecular therapeutics. To evaluate the performance of DAG-deepVASE in learning the complex molecular pathogenic mechanisms, we compared DAG-deepVASE with causalMGM and linear DG on the The Cancer Genome Atlas (TCGA) breast cancer of gene expression and clinical variables, such as PAM50 (*n* = 601 tumor samples). PAM50 is an important clinical feature to categorize breast tumors, which is defined by the tumor's expression of 50 genes (PAM50 genes) [86]. Therefore, we consider the causal directions from the genes to the PAM50 status as true positives. For our analysis, we chose 10 of the 50 genes (PAM50-defining genes) that are also included in the top 500 genes that have the highest variance across the samples (high-variance gene set). In addition to the 10 genes, we also considered 5 clinical variables, which are known to characterize breast tumors with the PAM50 status: estrogen receptor (ER) [87], progesterone receptor (PER) [87], human epidermal growth factor receptor (HER) [88], lymph node status [89], and tumor staging code [90].

**Figure 5:**
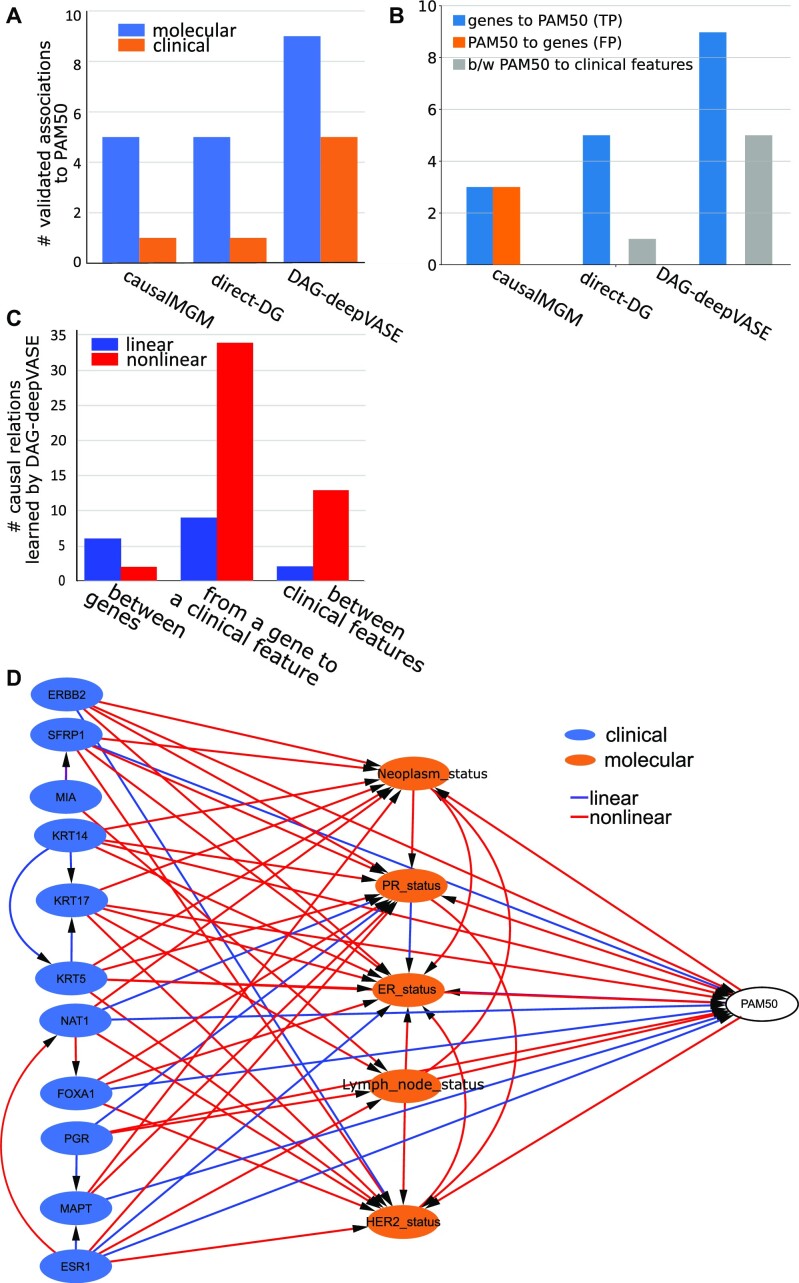
DAG-deepVASE on TCGA breast cancer data. (A) Number of validated associations from molecular (blue) and clinical (orange) variables to PAM50 identified by causalMGM, linear DG, and DAG-deepVASE. (B) Number of causalities identified by causalMGM, linear DG, and DAG-deepVASE. (C) Number of linear and nonlinear causalities DAG-deepVASE learned between 2 of the 10 genes, between a gene and a clinical variable, or between 2 of the 6 clinical variables. (D) Causalities inferred by DAG-deepVASE over 10 molecular variables, 5 clinical variables, and the PAM50 status as linear (purple) and nonlinear (red) by DAG-deepVASE. “person_neoplasm_cancer_status” refers to the state or condition of an individual's neoplasm. “PR_status,” “ER_status,” and “HER2_status” refer to the status of progesterone receptor, estrogen receptor, and human epidermal growth factor 2 receptor in the tumor sample, respectively.

In the first step of identifying associations between the 10 PAM50-defining genes with the highest variances and PAM50 status, DAG-deepVASE identified 9 of 10 associations, while causalMGM and linear-DG identified only 5 of them, attributing the 40% power increase of DAG-deepVASE to the identification of nonlinear associations. Between the 5 clinical variables and PAM50, DAG-deepVASE identified all 5 associations while causalMGM and linear DG identified only 1 association (20%) (Fig. 5A, Supplementary Figure S3A, B), suggesting that 80% (4 of 5) of the associations are nonlinear. In the second step of learning causal directions, DAG-deepVASE outperformed both causalMGM and linear DG, identifying true causalities from all 9 identified associations between the genes and PAM50 (Fig. 5B). On the other hand, causalMGM identified bidirectional causalities for 3 associations after the PC step removed the other 2 associations based on the conditional independence relationships. And linear DG identified the correct causalities on all 5 identified associations but was still missing causalities for the other 5 associations that linear DG could not identify. We did not assess the causal directions between the 5 clinical variables and PAM50 since the true causal directions are not clear between them. We tried different parameter settings of the methods to find that this trend holds true across the parameter settings (Supplementary Fig. S3C, Supplementary Table S4).

To ensure reproducibility of this finding, we further selected the top 20 genes from PAM50 genes with the largest variance in the data and evaluated the methods on the genes. In identifying the true associations, DAG-deepVASE identified 19 associations out of 20 (95.5%) while both causalMGM and linear DG identified 6 of them (30%) (Supplementary Fig. S3D). And, in learning the causal directions, DAG-deepVASE identified 19 true causalities from all the identified associations, while causalMGM identified 4 true and 2 false causalities out of the 6 associations and direct DG identified 6 true causalities from all the identified associations (Supplementary Fig. S3D). Altogether, the results demonstrate that DAG-deepVASE outperforms causalMGM and linear DG in identifying true associations, learning true causalities, and differentiating false causalities in the breast cancer data.To demonstrate how DAG-deepVASE enables us to understand complex pathogenic mechanisms across multiple regulatory layers in breast cancer, we expanded our analysis by investigating causalities among the 10 genes and the 6 clinical features, including the PAM50 status. Specifically, we inspected whether there are linear or nonlinear causalities in the following categories: causalities between a gene and a clinical feature and causalities between clinical features. First, while only a few causalities between genes were identified as nonlinear interactions (2 of 8 [25%]), most of the causalities between clinical features and between a clinical feature and a gene were identified as nonlinear causalities (13 of 15 [86.7%[ and 34 of 43 [79.1%], respectively). While many of them have been previously validated in clinical trials or biological experiments—for example, from ERBB2 (HER2) to PR, ERBB2 to ER, and ERBB2 to PAM50 [91] and KRT5 (keratin5) to PR and KRT5 to ER [92] (Fig. 5D)—the prevalence of nonlinear causality is consistent with the expectation since the clinical features, mostly hormone receptor status, are regulated through multiple biochemical pathways [93], and thus these relations are likely nonlinear. Other studies also advocate the nonlinear interactions of the clinical features by showing that incorporating nonlinearity in statistical models improves the prediction accuracy of their effects on breast tumor biology (e.g., in the transcriptional profile and survival analysis) [46–48]. Second, between a gene and a clinical feature, the method found that all 43 causal directions are from genes to clinical features (Fig. 5C). Since the clinical features in these data are mainly hormone receptor status, this result conforms to the expectation that genes code for the hormone receptor activity [94]. Incorporating all linear and nonlinear causalities under the categories sheds mechanistic insight into the complex tumorigenic process underlying breast cancer. For example, although the keratin genes (KRT5, KRT14, and KRT17) were found to interact in cancer genome studies [95–97], it was not clear how the cluster affects clinical features for cancer. Our result suggests that it is because the gene cluster is formed in linear interactions, but its effect on clinical features is mostly nonlinear. Altogether, DAG-deepVASE could identify nonlinear causalities, consisting of 74.2% of all causalities in these data, which would be missed by other existing methods. Identifying nonlinear causal relationships sheds insights into not only genetic interactions but also their interactions with clinical features of tumor biology.

## Discussion

We developed the first method, DAG-deepVASE, that explicitly learns both linear and nonlinear causal relationships in complex biological systems in high-dimensional molecular and clinical data. In complex biological systems, multiple regulatory layers (e.g., transcriptome and methylation layers) extensively interact [1, 3, 98, 99] and render variable interactions highly nonlinear. In the simulated data of diverse scenarios and biological data of various contexts (pediatric sepsis, TCGA breast cancer, BMI with nutrients and gut bacteria), DAG-deepVASE consistently outperforms existing methods in identifying known and new nonlinear causal relations. In the first step to identify associations, while DAG-deepVASE identifies all the linear associations that are identified by causalMGM and linear DG, the method identifies nonlinear associations through DNN, which shows the power ranging from 87% to 100% in identifying associations. In the second step to identify causalities from the identified associations, DAG-deepVASE inferred causalities with a high accuracy (ranging from 88% to 100%) while causalMGM learned bidirectional causalities on most of the associations and linear DG learned only a small number of identified associations correctly. causalMGM learned bidirectional causalities in our analyses because it returned a bidirectional causality between variables mediated or confounded by latent variables [8], which very likely exist in molecular and clinical datasets. In contrast, the second step in DAG-deepVASE imposes a model on input variables (${x_j}$) instead of on the conditional distribution of the association (distribution of ${x_j}|{x_i}$). This imposition guarantees to identify nonlinear associations even when the model for the association is misspecified due to absence of latent mediating variables.

To explicitly learn nonlinear associations, DAG-deepVASE leverages a DNN approach differently from other DNN-based causal inference methods by explicitly modeling nonlinearity in individual variable pairs. Previous DNN approaches have been proposed mainly to navigate the intractable search space for the optimal DAG. Although the studies showed that their local optimal DAGs are often comparable to the global ones obtained through expensive combinatorial search, these methods can also return only a stationary-point solution rather than the global optimum. For example, in our simulation experiments, NOTEARS and DAG-GNN showed generally less than 50% of power compared to DAG-deepVASE. However, our experiments also suggest that the approach of using DNN for navigating the search space may perform better when more samples are collected to construct a more comprehensive search space. For example, the DNN methods identified generally more true causalities when more samples are input (Fig. 2C, D and Supplementary Fig. S1C, D), though the improvement seems to come at the expense of high false positives (Fig. 2E and Supplementary Fig. S1E).

Another advantage of DAG-deepVASE is the knockoff framework to estimate effect size for nonlinear associations that prioritizes causal relations over simple correlation based on the exchangeability property of the knockoff framework (see Methods). The estimated effect size is significantly larger for validated causal relationships than for nonvalidated ones in both the BMI data (*P* = 0.02; Supplementary Fig. S2P) and the breast cancer data (*P* = 0.03; Supplementary Fig. S3D). Based on the rationale that the effect sizes of validated associations are more apparent and thus stronger, these results suggest that the effect sizes estimated by DAG-deepVASE make sense, and these can facilitate translatable findings of the causal relationships by selecting strong causal relations, either linear or nonlinear, to test in downstream experiments or clinical trials. However, care needs to be taken in interpreting the nonlinear effect size as it does not indicate the strength or the direction of causal relations.

DAG-deepVASE enables a further translatable understanding of complex diseases by putting linear and nonlinear associations together. In the subnetwork of pediatric sepsis data presented above, IFN-γ and TNF-α are connected through linear and nonlinear associations (gray nodes in Fig. 3B). Mouse experiments showed that the interaction between IFN-γ and TNF-α triggers inflammatory cell death, tissue damage, and mortality in acute immune diseases characterized by a “cytokine storm,” including lipopolysaccharide (LPS)–mediated sepsis [100]. While it is difficult to identify multiple cytokines involved in the complex interactions, DAG-deepVASE could identify the interactions between IFN-γ and TNF-α via multiple associations of both linear and nonlinear ones, including MCP-1/CCL2. Since MCP-1/CCL2 shows a protective role in a similar mouse model (a polymicrobial sepsis model with LPS) [101], DAG-deepVASE suggests a therapeutic potential to the detrimental interaction between IFN-γ and TNF-α.

Despite the clear advantages, DAG-deepVASE has some limitations in improving the clinical relevance of the findings. The first is that DAG-deepVASE cannot take nonordinal categorical variables and take only continuous and ordinal categorical variables that approximately follow Gaussian distribution since model-X knockoff assumes Gaussian distribution. In this article, this condition did not pose any problem as all variables of our interest were either continuous or ordinal categorical. However, in the future, we will generate the knockoff variables for nonordinal categorical variables based on a regression model for nonordinal categorical variables [102]. Second, while DAG-deepVASE can estimate the effect size, it does not estimate statistical significance. Thus, to identify significant causal relations in the future, we will estimate statistical significance of the likelihood ratio test we derived to determine the causal direction in causal inference step 1. Third, as with other methods of learning causalities from observational data, the validity of the learned causalities depends on how well the data comply with the 3 causal assumptions: Markov, faithfulness, and sufficiency. However, biological data could violate these assumptions and weaken the applicability of the inference results. For example, since multiple biological layers, such as genomic, transcriptomic, and epigenetic layers, often interact to render a phenotype in humans, confounders can occur in any of the layers. However, it is not always feasible to measure all variables from all the layers due to technical and practical reasons, indicating that the causal sufficiency assumption of no latent confounder would be hardly met for biological data. Thus, it is necessary to conduct further experiments or clinical trials to validate the causal relationship learned through DAG-deepVASE.

In summary, we developed DAG-deepVASE, which learns causal relationships in complex biological systems. DAG-deepVASE is the first method that uses a DNN approach to identify linear and nonlinear associations and learn their causal directions. DAG-deepVASE outperforms existing methods, causalMGM, and linear DG, in identifying known causal relations in various simulation scenarios and molecular and clinical datasets. In addition to known causalities, DAG-deepVASE identifies novel complex pathobiological interactions involving nonlinear causal relations, which is not possible using other methods. By applying the knockoff framework to DNN, DAG-deepVASE estimates effect size for nonlinear associations that prioritizes causal relations, which allows to prioritize future clinical and experimental validations. With these advantages, the application of DAG-deepVASE can help identify driver genes and therapeutic agents in biomedical studies and clinical trials.

## Methods

In developing our method, we followed the DOME (Data, Optimization, Model, and Evaluation) guidelines stated in [103]. Especially, we selected testing data that are representative of the domain (TCGA breast cancer for molecular data, gut microbiome and obesity data for metagenomics, and pediatric sepsis data for clinical data) per the Data guidelines. Their accessions are further detailed in the Availability of Supporting Data section. Per the Optimization guidelines, we performed experiments with various numbers of neuron layers (1–5 layers) and various numbers of neurons (10, 50, 100, 200, 400, and 600 neurons) in each layer on the simulation data (10 and 190 features in true and false causal relation to the outcome, respectively, generated for 1,000 samples) (Supplementary Fig. S4). These experiments justify the current design principle of DNN methods to put multiple layers of the neurons that is the same as the number of input features. For example, our experiments demonstrate that, to run on the simulation data, which consist of 100 features, DNN models of multiple layers of 100 neurons perform the best. In this article, we followed this design principle to implement our methods and reported all hyperparameters (Table 1) and optimization protocol under the Running Parameters of DAG-deepVASE section. Per the Model guidelines, we dockerized our method to make it easier for people to test and deploy. Lastly, per the Evaluation guidelines, we compare our method with the public method (causalMGM) and the simple (baseline) method (linear DG) on the same dataset.

### Pre- and postprocessing

To reduce false-positive discoveries, DAG-deepVASE carries out several pre- and postprocessing steps. As a preprocessing step, DAG-deepVASE filters out variable pairs that are conditionally independent on all the other variables based on inverse covariance (<0.0001), using a Python function in the package for machine learning optimization (scipy.linalg.inv). Although it is a common practice for computational causal inference under certain assumptions and the filtered-out nodes may not change the rest of the network, we made it optional since they can be important nodes for downstream analysis [104]. As another optional postprocessing step, DAG-deepVASE can detect a cycle (a nonempty tail in which the first and the last nodes are equal) in the network connecting the causal relations. Further, users can remove the cycle components by removing edges with their prior knowledge or DAG-deepVASE can automatically remove the edges with the least effect size it estimates.

### Running parameters of DAG-deepVASE

To identify linear and nonlinear associations in each dataset, we first performed preprocessing steps described in the Pre- and Postprocessing section. To identify linear associations after the steps, we ran Lee and Hastie's log-likelihood model [6] for all possible variable pairs ($x,y$ in Equation 1) with the penalty to select important variables (Equation 2). We set the sparsity penalty values of the likelihood function to 0.3 unless specified otherwise. Variable pairs that remained after applying the penalty are significant linear associations.

To identify nonlinear associations, we first built a deep neural network model consisting of the input layer, 2 hidden layers, and the output layer (Step 1–1 in Fig. 1). Assume that the input data have *p* input variables, and then we set the input layer with 2*p neurons, since we generated the knockoff variable for each input variable and combined them in a pairwise fashion in the input layer (Equations 3, 4). The combined input-knockoff neurons are fully connected to the hidden layers. For the case of *p* input variables, each hidden layer has *p* neurons, further transformed using the rectified linear unit activation function [105]. The initial weights for the hidden layer are generated using the Glorot normal initializer [106], which uses L1-regularization with the regularization parameter set to $O( {\sqrt {\frac{{2logp}}{n}} } )$. To train this model, mean squared error (MSE) is used to calculate the loss in comparison with the response on the output layer. To train the model's parameters with respect to the loss function, we used a stochastic gradient descent method called “Adam optimization.” Then, we ran it to identify variables that predict the outcome variable with a high effect size estimated in Equations 5, 6. Equations are described in the Algorithm of DAG-deepVASE section. All running parameters for the nonlinear module are summarized in Table 1. To identify nonlinear associations between all pairs, we ran this procedure repeatedly with each variable as outcome and all the rest as input. While the procedure was previously developed to identify input variables that can predict the outcome [49], DAG-deepVASE identified these prediction pairs as associated variables based on a widely accepted notion that a predictor and the outcome are statistically an associated pair. For theoretical understanding of the equations, readers are referred to the following section.

### Algorithm of DAG-deepVASE

Let *X* be the data matrix of interest with variables measured over *N* observations. ${x_i} \in X$ is the *M* -dimensional feature vector observed for sample *i*, consisting of *C* continuous variable set ${X_C}$ and *D* ordinal categorical variable set ${X_D}$ ($C + D\,\, = \,\,M)$. To systematically construct a DAG from both linear and nonlinear associations among variables, DAG-deepVASE leverages a well-established computational framework where variable associations are first identified, and their causal directions are then learned [5,7, 107].

In the first step, DAG-deepVASE selects linearly associated variables based on Lee and Hastie's log-likelihood [6] as follows.


(1) \begin{eqnarray*}
logp\left( {{X_C},{X_D},{\mathrm{\,\,\Theta }}} \right) &=& \mathop \sum \limits_k^C \mathop \sum \limits_l^C \left( { - \frac{1}{2}{\beta _{kl}}{X_{Ck}}{X_{Cl}}} \right)+\, \mathop \sum \limits_k^C {\alpha _k}{X_{Ck}}\\ &&+ \mathop \sum \limits_k^C \mathop \sum \limits_l^D {v_{kl}}\left( {{X_{Dl}}} \right){X_{Ck}} + \mathop \sum \limits_k^D \mathop \sum \limits_l^D {{\mathrm{\Phi }}_{{\mathrm{kl}}}}\left( {{X_{Dk}},{X_{Dl}}} \right)\\ &&- \log \left( Z \right), \end{eqnarray*}

where ${\mathrm{\Theta }}$ represents all of the model parameters; ${\beta _{kl}}$ is the interaction coefficient between 2 continuous variables, ${X_{Ck}}$ and ${X_{Cl}}$; ${\alpha _k}$ is the potential of continuous variable ${X_{Ck}}$; ${v_{kl}}$ is the interaction parameter between continuous variable ${X_{Ck}}$ with each index of the categorical variable ${X_{Dl}}$; and ${{\mathrm{\Phi }}_{kl}}$ is a matrix of interaction parameters between discrete variables ${X_{Dk}}$ and ${X_{Dl}}$ (indexed by their levels) [6]. If the data consist only of continuous variables, this model reduces to a multivariate Gaussian model with ${\beta _{kl}}$ coefficient as entries in the precision matrix. If only with categorical variables, this model is the popular pairwise Markov random field with potentials given ${{\mathrm{\Phi }}_{{\mathrm{kl}}}}$. While calculating the partition function *Z* can be expensive, it is possible to optimize the log-likelihood edge by edge [8] under the faithfulness and causal Markov assumptions. Overall, this equation models the log-likelihood of interactions of continuous variables and categorical variables as a multinomial linear regression. To ensure sparsity and select associated variables in the regression model, Sedgewick et al. [8] introduced sparsity penalties for associations between continuous variables, between a continuous and a categorical variable, and between categorical variables (${\lambda _{cc}},\,\,{\lambda _{cd}},\,\,{\lambda _{dd}}$, respectively) as follows:


(2) \begin{eqnarray*}
minimiz{e_{\mathrm{\Theta }}}\tilde l\left( {\mathrm{\Theta }} \right) + {\lambda _{cc}}\mathop \sum \limits_{i < j} \left| {{\beta _{ij}}} \right| + {\lambda _{cd}}\mathop \sum \limits_{i,j} {\left| {\left| {{v_{ij}}} \right|} \right|_2} + {\lambda _{dd}}\mathop \sum \limits_{i < j} {\left| {\left| {{{\mathrm{\Phi }}_{{\mathrm{ij}}}}} \right|} \right|_F}
\end{eqnarray*}

For balance estimation of the associations, DAG-deepVASE uses the same sparsity penalty (0.3 for all 3 interactions) and set the False Discovery Rate (FDR) level *q* as 0.05. After selecting the interactions, we report as effect size the coefficients in the model (${\beta _{ij}},\,\,{v_{ij}},$ or, $\,\,{{\mathrm{\Phi }}_{{\mathrm{ij}}}}$, corresponding to the type of the selected variables).

In the second step, DAG-deepVASE selects nonlinearly associated variables as follows. To identify nonlinear associations with ${x_i}$, DAG-deepVASE sets multiple perceptron layers between ${X_{\backslash i}} = \{ {{x_1},{x_2}, \ldots ,\,\,{x_{i - 1}},\,\,{x_{i + 1}},..,{x_M}} \}$ and ${x_i}$ (Step 1. Nonlinear association in Fig. 1) and estimates the effect size of the association between (${x_j} \in {X_{\backslash i}},$  ${x_i}$). To estimate the effect size, DAG-deepVASE generates model-X knockoff [108]. For input variables ${x_i}$ and ${x_j}$, the exchangeability property ensures that $( {{x_i},\,\,{x_j},\widetilde {{x_j}}} ){ = ^d}( {{x_i},\widetilde {{x_j}},{x_j}} ),$ where "=*^d^*" denotes equality in distribution. The exchangeability properties help prioritize causal relations with ${x_i}$ over simple correlations. For example, suppose $( {{x_i},{x_k}} )$ is a correlation without causal relation. Then, the feature exchangeability $( {{x_i},\,\,{x_k},\widetilde {{x_k}}} ){ = ^d}( {{x_i},\widetilde {{x_k}},{x_k}} )$ will hold and make their relationship measure $| {R{I_{ik}}} |$ and $| {\widetilde {R{I_{ik}}}} |$ exchangeable, which will make ${S_{ik}} = \,\,| {R{I_{ik}}} | - | {\widetilde {R{I_{ik}}}} |$ to follow a distribution symmetric around 0. On the other hand, suppose $( {{x_i},{x_j}} )$ is a causal relation. Then, ${S_{ij}}$ will indicate how deviated the relationship of (${x_i},$  ${x_j})$ is compared to the null hypothesis, leading to estimation of the effect size. The idea is that knockoff matrix $\tilde X$ is generated to mimic the correlation structure within *X* but minimizes the cross-correlation with the outcome variable [108]. Specifically, model-X knockoff variables for the set of random variables $X = {( {{x_1},\,\, \ldots ,\,\,{x_p}} )^T}$ of our interest are a new family of random variables $\tilde X = {( {\widetilde {{x_1}}, \ldots ,\,\,\widetilde {{x_p}}} )^T}$ that satisfy 2 properties: (i) ${( {X,\,\,\tilde X} )_{swap( s )}}{ = ^d}\,\,(X,\tilde X$) for any subset $S \subset \{ {1, \ldots ,M} \}$, where $swap( s )$ means swapping ${x_j}$ and $\widetilde {{x_j}}\,\,$ for each $j \in S$ and ${ = ^d}$ denotes equal in distribution, and (ii) $\tilde X \bot Y|X$, that is, $\tilde X$ is independent of *X* given outcome *Y*. Suppose ${x_j}\sim{\rm N}( {0,\,\,\Sigma } )$ with $\Sigma \in \,{{\mathrm{R}}^{M \times M}}$ the covariance matrix. A valid construction of $\widetilde {{x_j}}$ is


(3) \begin{eqnarray*}
\widetilde {{x_{j}}}|{x_{j}}\sim {\rm N}\left( {{x_{j}} - diag\left\{ S \right\}{\Sigma ^{ - 1}}{x_{j}},\,\,2diag\left\{ S \right\} - diag\left\{ S \right\}{\Sigma ^{ - 1}}diag\left\{ S \right\}} \right). \end{eqnarray*}

Model-X knockoffs can be sampled from the conditional distribution of $\widetilde {{x_i}}|{x_i}$ as follows:


(4) \begin{eqnarray*}
\left( {{x_j},\widetilde {{x_j}}} \right)\sim\,\,{\rm N}\left( {\left( {\begin{array}{@{}*{1}{c}@{}} 0\\ 0 \end{array}} \right),\left( {\begin{array}{@{}*{1}{c}@{}} \Sigma \\ {\Sigma - diag\left\{ S \right\}} \end{array}\begin{array}{@{}*{1}{c}@{}} {\Sigma - diag\left\{ S \right\}}\\ \Sigma \end{array}} \right)} \right) \end{eqnarray*}

In this sampling, the sensitivity of identifying ${x_j}$ can increase with a larger *S* since it will make the knockoffs more different from *S*, subjected to another constraint that *S* should make $\Sigma - diag\{ s \} \ge 0.$ By pairing knockoff variable $\widetilde {{x_j}}$ with the corresponding input variable ${x_j}$ and optimizing together for ${x_i}$, one can quantify the importance of ${x_j}$ in reference to $\widetilde {{x_j}}$. Specifically, let $W_i^{( 0 )} \in \,{{\mathrm{R}}^{M \times \,\,1}},\,\,W_i^{( 1 )} \in \,{{\mathrm{R}}^{M \times M}},\,\,W_i^{( 2 )} \in \,{{\mathrm{R}}^{M \times M}},\,\,and\,\,W_i^{( 3 )} \in \,{{\mathrm{R}}^{M \times \,\,1}}$ be the weight matrices connecting the input vector to the first hidden layer, the first hidden layer to the second hidden layer, the second hidden layer to the third hidden layer, and the third hidden layer to ${x_i}$, respectively. The weight estimates can be summarized into ${w_i} = W_i^{( 0 )} \otimes ( {W_i^{( 1 )}W_i^{( 2 )}W_i^{( 3 )}} )$, where $\otimes$ denotes the element-wise matrix operation. Also, let $r{i_{ji}}$ and $\widetilde {r{i_{ji}}}$ be the filter weight for ${x_j}$ and its knockoff counterpart $\widetilde {{x_j}}$. Then, variable importance values can be estimated for input and knockoff variables as follows:


(5) \begin{eqnarray*}
R{I_{ji}} = r{i_{ji}} \times {w_i}\,\,and\,\,\widetilde {R{I_{ji}}} = \widetilde {r{i_{ji}}} \times \widetilde {{w_i}}. \end{eqnarray*}

We use Adam to train this deep learning model with respect to the mean squared error loss, using an initial learning rate of 0.001 and batch size 10. With ${S_{ji}} = \,\,| {R{I_{ji}}} | - | {\widetilde {R{I_{ji}}}} |$, DAG-deepVASE estimates effect size on (${x_j} \in {X_{\backslash i}},$  ${x_i}$) adopted from [109, 108], which can be described in the following 2 options:


(6) \begin{eqnarray*}
T\,\, &=& \,\,min\left\{ {t \in S,\frac{{\left| {\left\{ {j:{S_{ji}} \le - \,\,t} \right\}} \right|}}{{\left| {\left\{ {j:{S_{ji}} \ge t} \right\}} \right|}}\,\, \le q} \right\}or\,\,{T_ + }\\ &=& min\left\{ {t \in S,\frac{{1 + \left| {\left\{ {j:{S_{ji}} \le - \,\,t} \right\}} \right|}}{{1{\mathrm{V}}\left| {\left\{ {j:{S_{ji}} \ge t} \right\}} \right|}}\,\, \le q} \right\}
\end{eqnarray*}

where *q* is a user-defined nominal false discovery rate and *T* or ${T_ + }$ is a threshold value for determining which features should be selected. We controlled FDR $q\,\, = \,\,0.05$ based on ${S_{ji}}$. While this setting has previously been used for a variable selection problem with respect to outcome [30–33], we extend this problem to estimate the nonlinear effect size for associated variables in this article. Since model-X knockoff assumes to follow a Gaussian distribution, we will include only continuous or ordinal categorical variables that approximately follow Gaussian distribution (using the Q–Q plot). We set parameters of DAG-deepVASE according to a guideline that utilized the knockoff framework for variable selection [49] (Table 1).

In the third step, for each identified variable association (${x_i},\,\,{x_j}$), whether linear or nonlinear, DAG-deepVASE determines the causal direction as extended from the DG framework as follows, calculated as


(7) \begin{eqnarray*}
DG\left( {G,Z} \right) = \mathop \sum \limits_{j = 1}^p dg\left( {{Z_j}|{Z_{Pa_j^G}}} \right), \end{eqnarray*}

where


\begin{eqnarray*}
dg\left( {{Z_j}|{Z_{Pa_j^G}}} \right) &=& \,\,\ell \left( {{{\hat \theta }_{mle}}|{Z_{\left\{ j \right\} \cup Pa_j^G}}} \right) - \ell \left( {{{\hat \theta }_{mle}}{\mathrm{|}}{Z_{Pa_j^G}}} \right)\\ &&- \frac{c}{2}\left| {{Z_j}} \right|\left| {{Z_{Pa_j^G}}} \right|\log \left( n \right), \end{eqnarray*}

where *c* is a penalty discount used to tune the density of the resulting graph. Also, $\ell ( {{{\hat \theta }_{mle}}|{Z_{sub}}} )$, which is the log-likelihood of a subset of Z, is computed using the Gaussian log-likelihood function in reference to ${\widehat \sum _{sub}}$, the partial covariance matrix for the input variables. Note $dg( {{Z_j}|{Z_{Pa_j^G}}} ) = logP( {{X_j}|{X_{Pa_j^G}}} )$ if the data have only continuous variables [13]. By maximum likelihood, the DG framework determines ${x_j}$ as causal and ${x_i}$ as effect if $dg( {{x_i}|{x_j}} ) > dg( {{x_j}|{x_i}} )\,\,$or $dg( {{x_i}|{x_j}} ) - dg( {{x_j}|{x_i}} ) > 0$. Due to multiplication commutativity,


(8) \begin{eqnarray*} \begin{array}{@{}l@{}} \,\,dg\left( {{x_i}|{x_j}} \right) - dg\left( {{x_j}|{x_i}} \right)\\ = \,\,\ell \left( {{{\hat \theta }_{mle}}|{x_{\left\{ {i,j} \right\}}}} \right) - \ell \left( {{{\hat \theta }_{mle}}{\mathrm{|}}{x_j}} \right) - \frac{c}{2}\left| {{x_i}} \right|\left| {{x_j}} \right|\log \left( N \right)\\ - \left( {\ell \left( {{{\hat \theta }_{mle}}|{x_{\left\{ {j,i} \right\}}}} \right) - \ell \left( {{{\hat \theta }_{mle}}{\mathrm{|}}{x_i}} \right) - \frac{c}{2}\left| {{x_j}} \right|\left| {{x_i}} \right|\log \left( {\mathrm{N}} \right)} \right)\\ = \ell \left( {{{\hat \theta }_{mle}}{\mathrm{|}}{x_i}} \right) - \ell \left( {{{\hat \theta }_{mle}}{\mathrm{|}}{x_j}} \right)\\ \,\, = \,\,l\left( {\frac{{{{\hat \theta }_{mle}}|{x_i}}}{{{{\hat \theta }_{mle}}|{x_j}}}} \right).(1) \end{array}
\end{eqnarray*}

After running this likelihood ratio test, we algorithmically remove the causal relations that create a cycle (a nonempty tail in which the first and the last nodes are equal) to ensure acyclicity by removing the one association with the least effect size (${S_{ji}})$.

### Simulation for nonlinear associations

The nonlinear simulation datasets were generated using a single index model [110–113]. Each simulation dataset consists of 3 parts: outcome variable $y = {( {{Y_1}, \ldots ,{Y_n}} )^T} \in \,{{\mathrm{R}}^{N \times 1}}$; a set of independently and identically distributed random variables $X \in \,{{\mathrm{R}}^{N \times M}}$, which have a different degree of nonlinear association with *y*; and a set of independently and identically distributed random variables $Z \in \,{{\mathrm{R}}^{Q \times M}}$, which have no association with *Y*. The following model was used to generate associated variable pairs ${x_i}$ and *Y*:


(9) \begin{eqnarray*}
{Y_i} = \alpha g\left( {x_i^T\beta } \right) + \left( {1 - \alpha } \right)x_i^T\gamma + {\varepsilon _i}
\end{eqnarray*}

where *g* is a nonlinear link function that we set to be a cube (${X^3})$ function, ${Y_i}$ is the outcome value, and ${\varepsilon _i}$ is noise added to the $ith$ outcome. $\alpha $ determines the proportion of the (non)linearity of the simulation where $\alpha \,\, = \,\,1$ determines the association of ${X_i}$ and ${Y_i}$ only with the nonlinear link function (complete-nonlinear) and $\alpha \,\, = \,\,0.5$ determines the association half by the nonlinear function and half by the linear function (partial-nonlinear). The distribution for noise $\varepsilon $ was simulated from $\mathcal{N}( {0,{\sigma ^2}{{\mathrm{{\rm I}}}_N}} )$, where $\sigma $ is set as 1. The rows of *X* were simulated independently from a distribution $\mathcal{N}( {0,\sum } )$ with a precision matrix ${\sum ^{ - 1}} = {( {{\rho ^{| {j - k} |}}} )_{1 \le j,\,\,k \le ( {q + p} )}}$ with $\rho \,\, = \,\,0.5$. A similar strategy has been used to assess the performance of deep learning methods developed for variable selection and causal inference, deepPINK [49] and DAG-GNN [42], respectively. In this article, we extended their methods by diversifying the degree of nonlinearity by adding the proportion of linearity $\alpha $. Also, note that this simulation satisfies the essential condition for causal inference, causal sufficiency. Specifically, *Y* is the direct product of *X* without a mediator (Equation 9). Since this means no latent confounder in the causal relationship from *X* to *Y*, it satisfies causal sufficiency. The other 2 essential causal assumptions, causal Markov and faithfulness, are not relevant to the simulation since there is no other variable in the simulation that is conditionally dependent or independent of *X* and *Y*. Altogether, this simulation experiment is designed to evaluate the performance of causal inference methods in a straightforward setting.

For each parameter combination (number of features and samples, complete- or partial-nonlinear), we ran various numbers of repetitions (50, 100, and 150) but report the results of 50 repetitions as different numbers of repetitions returned very similar results.

### Availability of supporting data

We used a simulation dataset, 2 public data, and 1 access-controlled data. Our simulation data are downloadable from our project website [114]. TCGA breast invasive carcinoma (BRCA) data were downloaded from the USCS Xena browser [115], available under the BRCA cohort, under the gene expression RNAseq section, on the IlluminaHiSeq (*n* = 1,218) TCGA Hub. It consists of the gene expression RNAseq dataset (dataset ID: TCGA.BRCA.sampleMap/HiSeqV2) and the clinical phenotype dataset (dataset ID: TCGA.BRCA.sampleMap/BRCA_clinicalMatrix). To investigate the dietary effect of the human gut microbiome, we downloaded cross-sectional data of 98 healthy volunteers from the DeepPINK resource site [116] that preprocessed the dataset collected from [85]. We also used access-controlled data of pediatric sepsis. The entire data are available upon request and after taking due steps for the rights and welfare of human research subjects involved in the study (regarding the institutional review board review). However, to ensure reproducibility of our findings, we uploaded a downsampled (70%) version of our datasets for the interactions of SIRS on the code and data repository site described below. The interactions of SIRS form the basis of our novel findings and include SIRS with heart rate, CRP, IFN-γ, CNS dysfunction, and IL-22. We ensured that our findings are reproduced using this dataset. Details of each data are given below.

### Breast cancer data

The gene expression RNAseq section in the Xena website includes the level 3 data estimates in the log2(x + 1)–transformed RNA-Seq by Expectation-Maximization (RSEM) normalized count obtained from the TCGA data coordination centers. The University of North Carolina TCGA genome characterization center experimentally measured the gene expression profile using the Illumina HiSeq 2000 RNA sequencing platform. Since we selected genes based on the expression variation, we did not use gene expression data with further normalization in the Xena website, such as pancan normalization or percentile normalization. For the gene expression dataset, we selected 500 or 2,000 expressed genes based on their variances. Then, we added ERBB2 (also known as HER2 or *neu*) to the selected gene set to the 500 genes selected above. ERBB2 was included due to its important role in human malignancies, especially for human breast cancers [117]. For the clinical dataset, we used 10 well-known clinical status features: PAM50 status (PAM50Call_RNAseq), HER2 status (HER2_Final_Status_nature2012), tumor stage (Converted_Stage_nature2012), tumor node status (Node_nature2012), the PR status (breast_carcinoma_progesterone_receptor_status), the ER status (breast_carcinoma_estrogen_receptor_status), the number of lymph nodes (lymph_node_examined_count), neoplasm cancer status (person_neoplasm_cancer_status), and pathologic stage information (pathologic_stage).

### Gut microbiome data

These data have 214 micronutrients and 87 genera from 90 healthy donors. They were between the ages of 18 and 40 and required to be free from any chronic GI disease, cardiac disease, diabetes mellitus, or immunodeficiency diseases; to have a normal bowel frequency (between once every 2 days and 3 times per day); to have BMI between 18.5 and 35. They had not taken antibiotics within 6 months prior to enrollment, proton pump inhibitors, H2 receptor antagonists, tricyclic antidepressants, narcotics, anticholinergic medications, laxatives, or antidiarrhea medications within 4 weeks of enrollment or nonsteroidal anti-inflammatory drugs, dietary supplements, or antacids within 2 weeks prior to enrollment.

The BMI data were evaluated based on the donors’ information, and the bacteria data were extracted using 16S ribosomal RNA sequencing from the stool samples. For a consistent result with previous analyses, we used the same data preprocessing procedure as previous computational work on the data [49]. In particular, the nutrient values were normalized using the residual method to adjust for caloric intake and then standardized [118]. Then, these data were log-ratio transformed to get rid of the sum-to-1 constraint and then centralized. Following [64], 0s were replaced with 0.5 before converting the data to a compositional form. With both the nutrient intake and genera composition as predictors, we treated BMI as the response.

### Pediatric sepsis data

The pediatric sepsis data were collected from 9 pediatric intensive care units in the Eunice Kennedy Shriver National Institutes of Child Health and Human Development Collaborative Pediatric Critical Care Research Network (including Children's Hospital of Pittsburgh, Children's Hospital of Philadelphia, Children's National Medical Center, Children's Hospital of Michigan, Nationwide Children's Hospital, Children's Hospital of Los Angeles, St. Louis Children's Hospital, C. S. Mott Children's Hospital, and Mattel Children's Hospital at the University of California, Los Angeles) [119]. Briefly, we collected blood samples and clinical data obtained from our previously published PHENOMS study [119]. Approval was obtained from the University of Utah Institutional Review Board, Central IRB #70976. Written informed consent was obtained from 1 or more parents/guardians for each child. Assent was garnered when the child was able. Patients were enrolled from 2015 to 2017. The CONSORT diagram and details of the clinical study protocol have been previously published [119]. In brief, children qualified for enrollment in PHENOMS if they (i) were aged from 44 weeks’ gestation to 18 years; (ii) were suspected of having infection, meeting 2 or more of 4 systemic inflammatory response criteria [120]; and (iii) had 1 or more organ failures [121]. Three consented and enrolled children who were excluded from reporting in the parent study manuscript due to a maximum per site enrollment of 81 patients to evenly distribute enrollment among the centers were additionally included in this analysis. Another work investigating these data is in progress, and thus these data are currently not deposited in the public domain yet. There originally were 55 candidate clinical features and 33 cytokine features measured from 404 children admitted. We removed features with a missing rate higher than 20% as well as highly correlated features (Pearson's correlation coefficient >0.6). Finally, we dropped samples with any missing data. As a result, this dataset provides 56 features (Table 3) from 281 samples with low correlations (<0.3 and >−0.44 in Pearson's correlation coefficient). In our analyses, some clinical terms were reported with the following abbreviations: GCS: Glasgow Coma Scale; CRP: C-reactive protein; SIRS: systemic inflammatory response syndrome: sCD163: soluble CD163; M-CSF: macrophage colony-stimulating factor. Table 3 has all the variables with full names. To address the high right-skewness of the clinical data, we employed the log transformation (log10) on the values.

**Table 3:** Variables in the pediatric sepsis data

Variable	Description of variable
**Demographic**	
Age	
**PRISM^a^**	
Low SBP	Lowest systolic blood pressure
High heart rate	Highest heart rate
Low temp	Lowest temperature
High temp	Highest temperature
GCS	Lowest Glasgow Coma Scale (GCS) score
Lower platelet	Lowest platelets
**Labs**	
Higher creatinine	Highest value from PRISM High Creatinine and High Creatinine
Low lymphocyte	Absolute lymphocyte count
Low hemoglobin	Hemoglobin
Low platelet	Platelet count
Ex vivo TNF-α	Blood endotoxin-stimulated TNF-α
SFASLigand	sFas ligand
sCD163	Soluble CD163
ADAMTS13	A disintegrin and metalloproteinase with a thrombospondin type 1 motif, member 13
**Organ failure**	
SIRS	Systemic inflammatory response syndrome criteria
**Cytokine**	
CRP	C-reactive protein
IFN-β	Interferon β
IL-22	Interleukin 22
IL-18	Interleukin 18
IL-18BP	Interleukin 18 binding protein
MIG-CXCL9	Chemokine (C-X-C motif) ligand 9 (CXCL9) or monokine induced by interferon gamma (MIG)
IL-1β	Interleukin 1β
IL-4	Interleukin 4
IL-6	Interleukin 6
IL-8	Interleukin 8
IL-10	Interleukin 10
IL-13	Interleukin 13
IL-17A	Interleukin 17A
IFN‐γ	Interferon γ
IP-10/CXCL10	C-X-C motif chemokine 10 (CXCL10) or interferon γ–induced protein 10 kDa (IP-10)
MCP-1/CCL2	Chemokine (C-C motif) ligand 2 (CCL2) or monocyte chemoattractant protein 1 (MCP1)
MIP-1α	Macrophage inflammatory protein 1α
MIP-1β	Macrophage inflammatory protein 1β
TNF-α	Tumor necrosis factor α
MCP-3	Monocyte chemotactic protein 3
IFNα2	Interferon α2
IL-1α	Interleukin 1α
IL-2Ra	Interleukin 2 receptor antagonists
IL-3	Interleukin 3
IL-16	Interleukin 16
M-CSF	Macrophage colony-stimulating factor
SCF	Stem cell factor
Trail	Trail
Ferritin	Ferritin

a PRISM: Pediatric RISk of Mortality.

## Supplementary Material

giad044_GIGA-D-22-00234_Original_Submission

giad044_GIGA-D-22-00234_Revision_1

giad044_GIGA-D-22-00234_Revision_2

giad044_GIGA-D-22-00234_Revision_3

giad044_GIGA-D-22-00234_Revision_4

giad044_Response_to_Reviewer_Comments_Original_Submission

giad044_Response_to_Reviewer_Comments_Revision_1

giad044_Response_to_Reviewer_Comments_Revision_2

giad044_Response_to_Reviewer_Comments_Revision_3

giad044_Reviewer_1_Report_Original_Submission Paola Lecca -- 10/30/2022 Reviewed

giad044_Reviewer_1_Report_Revision_1 Paola Lecca -- 2/11/2023 Reviewed

giad044_Reviewer_2_Report_Original_Submission Yunan Luo -- 10/31/2022 Reviewed

giad044_Reviewer_2_Report_Revision_1 Yunan Luo -- 2/6/2023 Reviewed

giad044_Reviewer_2_Report_Revision_2 Yunan Luo -- 4/7/2023 Reviewed

giad044_Supplemental_Files

## Data Availability

An archival copy of the code and supporting data is also available via the GigaScience database GigaDB [122].

## Additional Files

**Supplementary Figure S1**. AUC estimated for DAG-deepVASE and causalMGM on (A) 20 true and false associations and (B) 40 true and false associations, both under complete-nonlinear scenarios. (C) Average number, and standard error (error bar), of true associations in the partial-nonlinear scenario identified by DAG-deepVASE (red), causalMGM (gray), or linear-DG (yellow) over 50 runs in various simulation scenarios, varying the number of features and sample sizes. Average number, and standard error (error bar), of (D) true causalities and (E) false causalities identified by DAG-deepVASE (red), causalMGM (gray), or linear-DG (yellow) over 50 runs. DAG-deepVASE and linear-DG did not identify any false causalities.

**Supplementary Figure S2**. (A-J) Variable values against the BMI value window (1~5) that are identified as a nonlinear association to BMI (K-O) Variable values against the BMI value window (1~5) that are identified as a linear association to BMI. In the figures, KS is Kolmogorov-Smirnov (KS) test statistic, pval next to it is estimated from the KS test, rval is from a linear regression model, pval next to it is from the regression, and importance is the effect size measured in DAG-deepVASE under the knockoff framework. The gray area indicates 95% confidence intervals, the blue line indicates median values, and the red line represents a linearly regressed line (Methods). (P) Effect size of the 11 causalities identified uniquely by DAG-deepVASE out of the 16 validated causalities and 285 unvalidated factors to BMI out of 301 potential associations between nutrient intake/bacteria genera and BMI estimated by DAG-deepVASE.

**Supplementary Figure S3**. (A) PAM50-defining genes associated with the PAM50 status of patients identified by both DAG[1]deepVASE and causalMGM (purple) or uniquely by DAG-deepVASE (red). Both DAG-deepVASE and causalMGM could not identify a PAM50-defining gene (MIA) in a dotted line. (B) clinical features (e.g., hormone status) of the breast cancer samples associated with PAM50 that are identified by both DAG-deepVASE and causalMGM (purple) or uniquely by DAG-deepVASE (red). (C) Number of causalities, either from genes to PAM50 status (blue), which we deemed true positive, or from PAM50 status to genes (orange), which we deemed false positive, identified by causalMGM, linear-DG, and DAG-deepVASE run with various parameter settings. We also evaluated the number of causalities between PAM50 and clinical features (gray). (D) Effect size comparison between 9 PAM-defining and 491 other genes in the 500 genes with high variance of expression. P-value is estimated by t-test.

**Supplementary Table S1**. Average prediction power on true associations evaluated in simulation studies of different scenarios (using 50 repetitions of data). Supplementary Table S2. Causal relationships and the effect size identified in the Pediatric Sepsis Data by DAG-deepVASE. The causal relationships are under DAG-deepVASE column and the effect sizes are under Association effect size column. Supplementary Table S3. The effect size of all 301 potential associations between nutrient intake/bacteria genera and BMI estimated by DAG-deepVASE. Supplementary Table S4. Number of causalities identified by each combination of parameters for identifying associations (e.g., alpha and lambda as defined by causalMGM or DAG-deepVASE) and inferring causalities (e.g., alpha and penaltyDiscount as defined by PC or DG).

**Supplementary Table S2**. Causal relationships identified in the Pediatric Sepsis Data by causalMGMS.

**Supplementary Table S3**. The effect size of all 301 potential associations between nutrient intake/bacteria genera and BMI estimated by DAG-deepVASE.

**Supplementary Table S4**. Parameter setting for the simulation experiments.

## Abbreviations

AI: artificial intelligence; AUC: area under the receiver operating characteristic curve; BMI: body mass index; BRCA: breast invasive carcinoma; CNS: central nervous system; CRP: C-reactive protein; DAG: directed acyclic graph; DAG-deepVASE: directed acyclic graphs using deep learning variable selection; DG: degenerate Gaussian; DNN: deep neural network; ER: estrogen receptor; GI: gastrointestinal; HER: human epidermal growth factor receptor; IFN-γ: interferon γ; IL: interleukin; LPS: lipopolysaccharide; MCP-1: monocyte chemoattractant protein 1; M-CSF: macrophage colony-stimulating factor; MGM: mixed graphical model; PC: Peter and Clark; PER: progesterone receptor; SIRS: systemic inflammatory response syndrome; TNF-α: tumor necrosis factor α.

## Availability of Supporting Source Code and Requirements

Project name: DAG-deepVASE

Project homepage: https://github.com/ZhenjiangFan/DAG-deepVASE [114]

Operating system(s): Platform independent

Programming language: Python, Java, C, and R

License: MIT license

RRID: SCR_023612

Bio.tools ID: biotools:dag-deepvase

## References

[1] Kim S, Park HJ, Cui X et al. Collective effects of long-range DNA methylations predict gene expressions and estimate phenotypes in cancer. Sci Rep. 2020;10(1):3920. 32127627 10.1038/s41598-020-60845-2 PMC7054398

[2] Kim S, Bai Y, Fan Z et al. The microRNA target site landscape is a novel molecular feature associating alternative polyadenylation with immune evasion activity in breast cancer. Brief Bioinform. 2021;22:1–10. 32844230 10.1093/bib/bbaa191 PMC8138879

[3] Fan Z, Kim S, Bai Y et al. 3′-UTR shortening contributes to subtype-specific cancer growth by breaking stable ceRNA crosstalk of housekeeping genes. Front Bioeng Biotechnol. 2020;8:334. 32411683 10.3389/fbioe.2020.00334 PMC7201092

[4] Sedgewick AJ, Ramsey JD, Spirtes P., et al. Mixed graphical models for causal analysis of multi-modal variables. CoRR. 2017;1;35(7):1204–12.

[5] Loh P-L, Bühlmann P. High-dimensional learning of linear causal networks via inverse covariance estimation. J Mach Learn Res. 2014;15(1):3065–105.

[6] Lee J, Hastie T. Structure learning of mixed graphical models. J Mach Learn Res. 2013;31:388–96.

[7] Cui R, Groot, Heskes T. Copula PC algorithm for causal discovery from mixed data. 2016;9852: doi:10.1007/978-3-319-46227-1_24.

[8] Sedgewick AJ, Shi I, Donovan RM et al. Learning mixed graphical models with separate sparsity parameters and stability-based model selection. BMC Bioinf. 2016;17(5):S175. 10.1186/s12859-016-1039-0 PMC4905606 27294886

[9] Bottcher S . Learning Bayesian networks with mixed variables. Proc Eighth Int Workshop Artificial Intell Stat. 2001;R3:13–20.

[10] Romero V, Rumí R, Salmerón A. Learning hybrid Bayesian networks using mixtures of truncated exponentials. Int J Approximate Reasoning. 2006;42(1):54–68.

[11] Pearl J . Probabilistic Reasoning in Intelligent Systems: Networks of Plausible Inference. San Mateo, CA, US: Morgan Kaufmann, 1988.

[12] Spirtes P, Glymour C, Scheines R. *Causation, Prediction, and Search*. 2nd ed. The MIT Press, 2000; Available from: https://www.bibsonomy.org/bibtex/2e2b107e8fd3469c8b0e944ca37a559f3/mozaher.

[13] Chickering DM. Optimal structure identification with greedy search. CrossRef Listing of Deleted DOIs. 2000;1:507–54.

[14] Koivisto M, Sood K. Exact Bayesian structure discovery in Bayesian networks. J Mach Learn Res. 2004;5:549–73.

[15] Silander T, Myllymäki P. A simple approach for finding the globally optimal Bayesian network structure. 2006; Available from: https://arxiv.org/abs/1206.6875.

[16] Jaakkola T, Sontag D, Globerson A, et al. Learning Bayesian network structure using LP relaxations. PMLR. 2010;9:358–65.

[17] Cussens J . Bayesian network learning with cutting planes. In Proceedings of the Twenty-Seventh Conference on Uncertainty in Artificial Intelligence. Corvallis, Oregon: AUAI Press. 2011;153–60.

[18] Yuan C, Malone B, Wu X. Learning Optimal Bayesian Networks Using A* Search. In IJCAI International Joint Conference on Artificial Intelligence, 2011; doi:10.5591/978-1-57735-516-8/IJCAI11-364.

[19] Gao T, Wei D. Parallel Bayesian network structure learning. Proc 35th Int Conf Machine Learning. 2018;80:1685–94.

[20] Zhang X, Zhao X, He K, et al. Inferring gene regulatory networks from gene expression data by path consistency algorithm based on conditional mutual information. Bioinformatics. 2012;28(1):98–104. 22088843 10.1093/bioinformatics/btr626

[21] Maathuis MH, Colombo D, Kalisch M et al. Predicting causal effects in large-scale systems from observational data. Nat Methods. 2010;7(4):247–8. 20354511 10.1038/nmeth0410-247

[22] Le TD, Liu L, Tsykin A et al. Inferring microRNA–mRNA causal regulatory relationships from expression data. Bioinformatics. 2013;29(6):765–71. 23365408 10.1093/bioinformatics/btt048

[23] Zhang J, Le TD, Liu L et al. Inferring condition-specific miRNA activity from matched miRNA and mRNA expression data. Bioinformatics. 2014;30(21):3070–7. 25061069 10.1093/bioinformatics/btu489 PMC4609012

[24] Zhang J, Le TD, Liu L et al. Identifying direct miRNA–mRNA causal regulatory relationships in heterogeneous data. J Biomed Inform. 2014;52:438–47. 25181465 10.1016/j.jbi.2014.08.005

[25] Silverstein C, Brin S, Motwani R, et al. Scalable techniques for mining causal structures. Data Min Knowl Discov. 2000;4(2):163–92.

[26] Andrews B, Ramsey J, Cooper GF. Learning high-dimensional directed acyclic graphs with mixed data-types. Proc Mach Learn Res. 2019;104:4–21. 31453569 PMC6709674

[27] Schwarz G . Estimating the dimension of a model. Ann Statist. 1978;38(2):461–4.

[28] Neto EC, Keller M, Attie AD et al. Causal graphical models in systems genetics: a unified framework for joint inference of causal network and genetic architecture for correlated phenotypes. Ann Appl Stat. 2010;4(1):320–39. 21218138 10.1214/09-aoas288 PMC3017382

[29] Kruijer W, Behrouzi P, Bustos-Korts D et al. Reconstruction of networks with direct and indirect genetic effects. Genetics. 2020;214(4):781–807. 32015018 10.1534/genetics.119.302949 PMC7153926

[30] Yazdani A, Yazdani A, Samiei A et al. Generating a robust statistical causal structure over 13 cardiovascular disease risk factors using genomics data. J Biomed Inform. 2016;60:114–9. 26827624 10.1016/j.jbi.2016.01.012 PMC4886234

[31] Yazdani A, Yazdani A, Saniei A et al. A causal network analysis in an observational study identifies metabolomics pathways influencing plasma triglyceride levels. Metabolomics. 2016;12(6):104. 27330524 10.1007/s11306-016-1045-2 PMC4869741

[32] Yazdani A, Yazdani A, Bowman TA, et al. Arachidonic acid as a target for treating hypertriglyceridemia reproduced by a causal network analysis and an intervention study. Metabolomics. 2018;14(6):78. 30830364 10.1007/s11306-018-1368-2

[33] Yazdani A, Yazdani A, Elsea S, et al. Genome analysis and pleiotropy assessment using causal networks with loss of function mutation and metabolomics. BMC Genomics. 2019;20(1):395. 31113383 10.1186/s12864-019-5772-4 PMC6528192

[34] Triantafillou S, Lagani V, Heinze-Deml C, et al. Predicting causal relationships from biological data: applying automated causal discovery on mass cytometry data of human immune cells. Sci Rep. 2017;7(1):12724. 28983114 10.1038/s41598-017-08582-x PMC5629212

[35] Rothenhäusler D, Heinze C, Peters J, et al. BACKSHIFT: learning causal cyclic graphs from unknown shift interventions. Adv Neural Inf Process Syst. 2015;(1):1513–21.

[36] Sedgewick AJ, Buschur K, Shi I, et al. Mixed graphical models for integrative causal analysis with application to chronic lung disease diagnosis and prognosis. Bioinformatics. 2019;35(7):1204–12. 30192904 10.1093/bioinformatics/bty769 PMC6449754

[37] Nie S, Maua DD, de Campos C et al. Advances in learning Bayesian networks of bounded treewidth. Adv Neural Inf Process Syst. 2014;27. https://proceedings.neurips.cc/paper/2014/file/3948ead63a9f2944218de038d8934305-Paper.pdf

[38] Scanagatta M, de Campos C, Corani G, et al. Learning Bayesian networks with thousands of variables. Adv Neural Inf Proc Syst. 2015;28. https://proceedings.neurips.cc/paper/2015/file/2b38c2df6a49b97f706ec9148ce48d86-Paper.pdf

[39] Chen EY-J, Shen Y, Choi A et al. Learning Bayesian networks with ancestral constraints. Adv Neural Inf Process Syst. 2016;29. https://proceedings.neurips.cc/paper/2016/file/144a3f71a03ab7c4f46f9656608efdb2-Paper.pdf

[40] Rantanen K, Hyttinen A, Järvisalo M. Discovering causal graphs with cycles and latent confounders: an exact branch-and-bound approach. Int J Approximate Reasoning. 2020;117:29–49.

[41] Zheng X, Aragam B, Ravikumar K, et al. DAGs with NO TEARS: continuous optimization for structure learning. Adv Neural Inf Process Syst. 2018;31. https://proceedings.neurips.cc/paper/2018/file/e347c51419ffb23ca3fd5050202f9c3d-Paper.pdf

[42] Yu Y, Chen J, Gao T, et al. DAG-GNN: DAG structure learning with graph neural networks. 36th Int Conf Mach Learn ICML. 2019:12395–406.

[43] Zheng X, Dan C, Aragam B, et al. Proceedings of the Twenty Third International Conference on Artificial Intelligence and Statistics. PMLR. 2020;108:3414–25.

[44] Higgins J . Nonlinear systems in medicine. Yale J Biol Med. 2002;75:247–60. 14580107 PMC2588816

[45] Trefois C, Antony MA, Goncalves J, et al. Critical transitions in chronic disease: transferring concepts from ecology to systems medicine. Curr Opin Biotechnol. 2015;34:48–55. 25498477 10.1016/j.copbio.2014.11.020

[46] Naik N, Madani A, Esteva A et al. Deep learning-enabled breast cancer hormonal receptor status determination from base-level H&E stains. Nat Commun. 2020;11(1):5727. 33199723 10.1038/s41467-020-19334-3 PMC7670411

[47] Lebedeva G, Yamaguchi A, Langdon S et al. A model of estrogen-related gene expression reveals non-linear effects in transcriptional response to tamoxifen. BMC Syst Biol. 2012;6(1):138. 23134774 10.1186/1752-0509-6-138 PMC3573949

[48] Perera M, Tsokos C. A statistical model with non-linear effects and non-proportional hazards for breast cancer survival analysis. ABCR. 2018;07:65–89.

[49] Lu YY, Fan Y, Lv J, et al. Deeppink: reproducible feature selection in deep neural networks. Adv Neural Inf Process Syst. 2018;8676–86.

[50] Qin Y, Kernan K, Fan Z, et al. Four computable 24-hour pediatric sepsis phenotypes have different inflammation profiles and heterogeneous outcome with anti-inflammatory therapies. Crit Care. 2022;7;26(1). 10.1186/s13054-022-03977-3 PMC9077858 35526000

[51] Crayne CB, Albeituni S, Nichols KE et al. The immunology of macrophage activation syndrome. Front Immunol. 2019;10:119. 30774631 10.3389/fimmu.2019.00119 PMC6367262

[52] Ushach I, Zlotnik A. Biological role of granulocyte macrophage colony-stimulating factor (GM-CSF) and macrophage colony-stimulating factor (M-CSF) on cells of the myeloid lineage. J Leukoc Biol. 2016;100(3):481–9. 27354413 10.1189/jlb.3RU0316-144R PMC4982611

[53] Deshmane SL, Kremlev S, Amini S et al. Monocyte chemoattractant protein-1 (MCP-1): an overview. J Interferon Cytokine Res. 2009;29(6):313–26. 19441883 10.1089/jir.2008.0027 PMC2755091

[54] Zhu L, Zhao Q, Yang T et al. Cellular metabolism and macrophage functional polarization. Int Rev Immunol. 2015;34(1):82–100. 25340307 10.3109/08830185.2014.969421

[55] Dige A, Støy S, Thomsen KL et al. Soluble CD163, a specific macrophage activation marker, is decreased by anti-TNF-α antibody treatment in active inflammatory bowel disease. Scand J Immunol. 2014;80(6):417–23. 25346048 10.1111/sji.12222

[56] Rittig N, Svart M, Jessen N et al. Macrophage activation marker sCD163 correlates with accelerated lipolysis following LPS exposure: a human-randomised clinical trial. Endocr Connect. 2018;7(1):107–14. 29295869 10.1530/EC-17-0296 PMC5754508

[57] Finn AV, Nakano M, Polavarapu R et al. Hemoglobin directs macrophage differentiation and prevents foam cell formation in human atherosclerotic plaques. J Am Coll Cardiol. 2012;59(2):166–77. 22154776 10.1016/j.jacc.2011.10.852 PMC3253238

[58] Fleming S, Thompson M, Stevens R et al. Normal ranges of heart rate and respiratory rate in children from birth to 18 years of age: a systematic review of observational studies. Lancet. 2011;377(9770):1011–8. 21411136 10.1016/S0140-6736(10)62226-X PMC3789232

[59] Jury DR. Serum creatinine concentration in children: normal values for sex and age. N Z Med J. 1979;90(649):453–6. 294520

[60] Shearer WT, Rosenblatt HM, Gelman RS et al. Lymphocyte subsets in healthy children from birth through 18 years of age: the pediatric AIDS clinical trials group P1009 study. J Allergy Clin Immunol. 2003;112(5):973–80. 14610491 10.1016/j.jaci.2003.07.003

[61] Blankson H, Stakkestad JA, Fagertun H, et al. Conjugated linoleic acid reduces body fat mass in overweight and obese humans. J Nutr. 2000;130(12):2943–8. 11110851 10.1093/jn/130.12.2943

[62] Chiu C-M, Huang WC, Weng SL et al. Systematic analysis of the association between gut flora and obesity through high-throughput sequencing and bioinformatics approaches. Biomed Res Int. 2014;2014:906168. 25202708 10.1155/2014/906168 PMC4150407

[63] Vanhala M, Saltevo J, Soininen P et al. Serum omega-6 polyunsaturated fatty acids and the metabolic syndrome: a longitudinal population-based cohort study. Am J Epidemiol. 2012;176(3):253–60. 22791741 10.1093/aje/kwr504

[64] Lin W, Shi, Feng R et al. Variable selection in regression with compositional covariates. Biometrika. 2014;101(4):785–97.

[65] Pimpin L, Jebb S, Johnson L et al. Dietary protein intake is associated with body mass index and weight up to 5 y of age in a prospective cohort of twins. Am J Clin Nutr. 2016;103(2):389–97. 26718416 10.3945/ajcn.115.118612 PMC4733258

[66] Rabot S, Membrez M, Blancher F, et al. High fat diet drives obesity regardless the composition of gut microbiota in mice. Sci Rep. 2016;6(1):32484. 27577172 10.1038/srep32484 PMC5006052

[67] Yang Q . Gain weight by ‘going diet?’ Artificial sweeteners and the neurobiology of sugar cravings: neuroscience 2010. Yale J Biol Med. 2010;83(2):101–8. 20589192 PMC2892765

[68] Yun Y, Kim HN, Kim SE, et al. Comparative analysis of gut microbiota associated with body mass index in a large Korean cohort. BMC Microbiol. 2017;17(1):151. 28676106 10.1186/s12866-017-1052-0 PMC5497371

[69] Reeds DN, Mohammed BS, Klein S, et al. Metabolic and structural effects of phosphatidylcholine and deoxycholate injections on subcutaneous fat: a randomized, controlled trial. Aesthetic Surg J. 2013;33(3):400–8. 10.1177/1090820X13478630 PMC3667691 23439063

[70] Kuang Y-S, Lu JH, Li SH et al. Connections between the human gut microbiome and gestational diabetes mellitus. Gigascience. 2017;6(8):1–12. 10.1093/gigascience/gix058 PMC5597849 28873967

[71] Yang YJ, Kim YJ, Yang YK et al. Dietary flavan-3-ols intake and metabolic syndrome risk in Korean adults. Nutr Res Pract. 2012;6(1):68–77. 22413043 10.4162/nrp.2012.6.1.68 PMC3296925

[72] Merx MW, Weber C. Sepsis and the heart. Circulation. 2007;116(7):793–802. 17698745 10.1161/CIRCULATIONAHA.106.678359

[73] Ma L, Zhang H, Yin YL, et al. Role of interleukin-6 to differentiate sepsis from non-infectious systemic inflammatory response syndrome. Cytokine. 2016;88:126–35. 27599258 10.1016/j.cyto.2016.08.033

[74] Mitaka C. Clinical laboratory differentiation of infectious versus non-infectious systemic inflammatory response syndrome. Clin Chim Acta. 2005;351(1):17–29. 15563869 10.1016/j.cccn.2004.08.018

[75] Nakanishi K . Unique action of interleukin-18 on T cells and other immune cells. Front Immunol. 2018;9:763. 29731751 10.3389/fimmu.2018.00763 PMC5920033

[76] Schoenborn JR, Wilson CB. Regulation of interferon-gamma during innate and adaptive immune responses. Adv Immunol. 2007;96:41–101. 17981204 10.1016/S0065-2776(07)96002-2

[77] Stanley AC, Lacy P. Pathways for cytokine secretion. Physiology. 2010;25(4):218–29. 20699468 10.1152/physiol.00017.2010

[78] Leonard WJ, Lin J-X. Cytokine receptor signaling pathways. J Allergy Clin Immunol. 2000;105(5):877–88. 10808165 10.1067/mai.2000.106899

[79] Tate W, Walker M, Sweetman E, et al. Molecular mechanisms of neuroinflammation in ME/CFS and long COVID to sustain disease and promote relapses. Front Neurol. 2022;13:877772. 35693009 10.3389/fneur.2022.877772 PMC9174654

[80] Zhao L, Gao Y, Guo S et al. Sepsis-associated encephalopathy: insight into injury and pathogenesis. CNS Neurol Disord Drug Targets. 2021;20(2):112–24. 33208082 10.2174/1871527319999201117122158

[81] Weber GF, Schlautkötter S, Kaiser-Moore S, et al. Inhibition of interleukin-22 attenuates bacterial load and organ failure during acute polymicrobial sepsis. Infect Immun. 2007;75(4):1690–7. 17261606 10.1128/IAI.01564-06 PMC1865721

[82] Manicka S, Johnson K, Levin M, et al. Biological regulatory networks are less nonlinear than expected by chance. bioRxiv. 2021; doi:10.1186/s13054-022-03977-3.

[83] Kapitaniak T, Jafari S. Nonlinear effects in life sciences. Eur Phys J Spec Top. 2018;227(7):693–6.

[84] Stoof R, Goñi-Moreno Á. Modelling co-translational dimerization for programmable nonlinearity in synthetic biology. J R Soc Interface. 2020;17(172):20200561. 33143595 10.1098/rsif.2020.0561 PMC7729047

[85] Wu GD, Chen J, Hoffmann C et al. Linking long-term dietary patterns with gut microbial enterotypes. Science. 2011;334(6052):105–8. 21885731 10.1126/science.1208344 PMC3368382

[86] Koboldt DC, Fulton RS, McLellan MD, et al. Comprehensive molecular portraits of human breast tumours. Nature. 2012;490(7418):61–70. 23000897 10.1038/nature11412 PMC3465532

[87] Pascual T, Martin M, Fernández-Martínez A et al. A pathology-based combined model to identify PAM50 non-luminal intrinsic disease in hormone receptor-positive HER2-negative breast cancer. Front Oncol. 2019;9:303. 31106144 10.3389/fonc.2019.00303 PMC6498671

[88] Nielsen TO, Parker JS, Leung S et al. A comparison of PAM50 intrinsic subtyping with immunohistochemistry and clinical prognostic factors in tamoxifen-treated estrogen receptor–positive breast cancer. Clin Cancer Res. 2010;16(21):5222–32. 20837693 10.1158/1078-0432.CCR-10-1282 PMC2970720

[89] Rossing M, Pedersen CB, Tvedskov T et al. Clinical implications of intrinsic molecular subtypes of breast cancer for sentinel node status. Sci Rep. 2021;11(1):2259. 33500440 10.1038/s41598-021-81538-4 PMC7838175

[90] Mittendorf EA, Bartlett JMS, Lichtensztajn DL, et al. Incorporating biology into breast cancer staging: American Joint Committee on Cancer, eighth edition, revisions and beyond. Am Soc Clin Oncol Educ Book. 2018;38:38–46. 30231409 10.1200/EDBK_200981

[91] Onitilo AA, Engel JM, Greenlee RT et al. Breast cancer subtypes based on ER/PR and Her2 expression: comparison of clinicopathologic features and survival. Clin Med Res. 2009;7(1–2):4–13. 19574486 10.3121/cmr.2009.825 PMC2705275

[92] Dai X, Chen A, Bai Z. Integrative investigation on breast cancer in ER, PR and HER2-defined subgroups using mRNA and miRNA expression profiling. Sci Rep. 2014;4(1):6566. 25338681 10.1038/srep06566 PMC4206873

[93] Brooks AJ, Wooh JW, Tunny KA et al. Growth hormone receptor; mechanism of action. Int J Biochem Cell Biol. 2008;40(10):1984–9. 17888716 10.1016/j.biocel.2007.07.008

[94] Harden K, Klump KL. Introduction to the special issue on gene-hormone interplay. Behav Genet. 2015;45(3):263–7. 25903987 10.1007/s10519-015-9717-7 PMC4445642

[95] Coolen MW, Stirzaker C, Song JZ et al. Consolidation of the cancer genome into domains of repressive chromatin by long-range epigenetic silencing (LRES) reduces transcriptional plasticity. Nat Cell Biol. 2010;12(3):235–46. 20173741 10.1038/ncb2023 PMC3058354

[96] Ashida S, Orloff MS, Bebek G, et al. Integrated analysis reveals critical genomic regions in prostate tumor microenvironment associated with clinicopathologic phenotypes. Clin Cancer Res. 2012;18(6):1578–87. 22275508 10.1158/1078-0432.CCR-11-2535

[97] Flaherty P, Wiratchotisatian, Lee JA, et al. MAP clustering under the gaussian mixture model via mixed integer nonlinear optimization. 2019. Available from: 10.48550/arXiv.1911.04285.

[98] Park HJ, Ji P, Kim S, et al. 3′ UTR shortening represses tumor-suppressor genes in trans by disrupting ceRNA crosstalk. Nat Genet. 2018;50:783–9. 29785014 10.1038/s41588-018-0118-8 PMC6689271

[99] Kim S, Forno E, Zhang R, et al. Expression quantitative trait methylation analysis reveals methylomic associations with gene expression in childhood asthma. Chest. 2020;158:1841–56. 32569636 10.1016/j.chest.2020.05.601 PMC7674990

[100] Karki R, Sharma BR, Tuladhar S, et al. Synergism of TNF-α and IFN-γ triggers inflammatory cell death, tissue damage, and mortality in SARS-CoV-2 infection and cytokine shock syndromes. Cell. 2021;184(1):149–168.e17. 33278357 10.1016/j.cell.2020.11.025 PMC7674074

[101] Gomes RN, Teixeira-Cunha MG, Figueiredo RT et al. Bacterial clearance in septic mice is modulated by MCP-1/CCL2 and nitric oxide. Shock. 2013;39(1):63–69. 23247123 10.1097/SHK.0b013e31827802b5 PMC3592381

[102] Kormaksson M, Kelly LJ, Zhu X et al. Sequential knockoffs for continuous and categorical predictors: with application to a large psoriatic arthritis clinical trial pool. Stat Med. 2021;40(14):3313–28. 33899260 10.1002/sim.8955

[103] “DOME-ML.” https://dome-ml.org/ (last accessed on 9th May 2023).

[104] Yazdani A, Yazdani A, Samiei A et al. Identification, analysis, and interpretation of a human serum metabolomics causal network in an observational study. J Biomed Inform. 2016;63:337–43. 27592308 10.1016/j.jbi.2016.08.017

[105] Agarap AF . Deep learning using rectified linear units (ReLU). 2018.Available from: 10.48550/arXiv.1803.08375.

[106] Glorot X, Bengio Y. Understanding the difficulty of training deep feedforward neural networks. Proc 13th Int Conf Artificial Intell Stat. 2010;9:249–56.

[107] Glymour C, Zhang K, Spirtes P. Review of causal discovery methods based on graphical models. Front Genet. 2019;10:524. 31214249 10.3389/fgene.2019.00524 PMC6558187

[108] Candès E, Fan Y, Janson L et al. Panning for gold: ‘model-X’ knockoffs for high dimensional controlled variable selection. J R Stat Soc Ser B Stat Methodol. 2018;80(3):551–77.

[109] Barber RF, Candes EJ. Controlling the false discovery rate via knockoffs. Ann Statist. 2015;43(5):2055–85.

[110] Hardle W, Stoker TM. Investigating smooth multiple regression by the method of average derivatives. J Am Stat Assoc. 1989;84(408):986–95.

[111] Ichimura H . Semiparametric least squares (SLS) and weighted SLS estimation of single-index models. J Econometrics. 1993;58(1):71–120.

[112] Carroll RJ, Fan J, Gijbels I et al. Generalized partially linear single-index models. J Am Stat Assoc. 1997;92(438):477–89.

[113] Wang L, Yang L. Spline estimation of single-index models. Stat Sin. 2009;19(2):765–83.

[114] DAG-deepVASE. *GitHub*. 2023. https://github.com/ZhenjiangFan/DAG-deepVASE

[115] “USCS Xena.” https://xena.ucsc.edu/ (last accessed date 5th June 2019).

[116] “deepPINK resource site.” https://noble.gs.washington.edu/proj/DeepPINK/ (last accessed date 2nd April 2021).

[117] Slamon DJ, Godolphin W, Jones LA et al. Studies of the HER-2/neu proto-oncogene in human breast and ovarian cancer. Science. 1989;244(4905):707–12. 2470152 10.1126/science.2470152

[118] Chen J, Li H. Variable selection for sparse Dirichlet-multinomial regression with an application to microbiome data analysis. Ann Appl Stat. 2013;7(1):418–42. 10.1214/12-AOAS592 PMC3846354 24312162

[119] Carcillo JA, Berg RA, Wessel D et al. A multicenter network assessment of three inflammation phenotypes in pediatric sepsis-induced multiple organ failure. Pediatr Crit Care Med. 2019;20:1137–46. 31568246 10.1097/PCC.0000000000002105 PMC8121153

[120] Goldstein B, Giroir B, Randolph A. International pediatric sepsis consensus conference: definitions for sepsis and organ dysfunction in pediatrics. Pediatr Crit Care Med. 2005;6(1):2–8. 15636651 10.1097/01.PCC.0000149131.72248.E6

[121] Villeneuve A, Joyal J-S, Proulx F et al. Multiple organ dysfunction syndrome in critically ill children: clinical value of two lists of diagnostic criteria. Ann Intensive Care. 2016;6(1):40. 27130424 10.1186/s13613-016-0144-6 PMC4851677

[122] Fan Z, Kernan K-F, Sriram A, et al. Supporting data for “Deep Neural Networks with Knockoff Features Identify Nonlinear Causal Relations and Estimate Effect Sizes in Complex Biological Systems.” GigaScience Database. 2023.Available from: 10.5524/102387. PMC10316696 37395630

