# Supplementary material for: Deep neural networks with knockoff features identify nonlinear causal relations and estimate effect sizes in complex biological systems
Source: Gigascience. 2023 Jul 3;12:giad044. doi: 10.1093/gigascience/giad044 (PMC10316696; doi:10.1093/gigascience/giad044)
Supplement: giad044_GIGA-D-22-00234_Original_Submission [file giad044_giga-d-22-00234_original_submission.pdf]

GigaScience

Deep neural networks with knockoff features identify nonlinear causal relations and estimate effect sizes in complex biological systems

--Manuscript Draft--

**Manuscript Number:**	GIGA-D-22-00234
**Full Title:**	Deep neural networks with knockoff features identify nonlinear causal relations and estimate effect sizes in complex biological systems
**Article Type:**	Technical Note
**Funding Information:**	National Institute of Health (R01GM108618) — Dr. Kate Kernan, Dr. Joseph Carcillo<br>National Institue of Health (R01GM108618) — Dr Hyun Jung Park<br>National Institute of Health (HL153792) — Dr. Soyeon Kim<br>National Institute of Health (AI123366) — Dr. Scott Canna
**Abstract:**	Background: Learning the causal structure helps identify risk factors, disease mechanisms, and candidate therapeutics for complex diseases [1]–[3]. However, although complex biological systems are characterized by non-linear associations, existing bioinformatic methods of causal inference cannot identify the nonlinear relationships and estimate their effect size [4]–[8]. Results: To overcome these limitations, we developed the first computational method that learns both linear and nonlinear causal relations and estimates the effect size using a deep-neural network approach coupled with the knockoff framework [9], named causal Directed Acyclic Graphs using deep-learning VAriable SElection (DAG-deepVASE). Using simulation data of diverse scenarios and identifying known and novel causal relations in molecular and clinical data of various diseases, we demonstrated that DAG-deepVASE consistently outperforms existing methods in identifying true and known causal relations. In the analyses, we also illustrate how identifying nonlinear causal relations and estimating their effect size help understand the complex disease pathobiology, which is not possible using other methods. Conclusions: With these advantages , the application of DAG-deepVASE can help identify driver genes and therapeutic agents in biomedical studies and clinical trials.
**Corresponding Author:**	Hyun Jung Park<br>University of Pittsburgh<br>Pittsburgh, PA UNITED STATES
**Corresponding Author Secondary Information:**	
**Corresponding Author's Institution:**	University of Pittsburgh
**Corresponding Author's Secondary Institution:**	
**First Author:**	Zhenjiang Fan
**First Author Secondary Information:**	
**Order of Authors:**	Zhenjiang Fan
	Kate Kernan
	Panayiotis Benos
	Scott Canna
	Joseph Carcillo
	Soyeon Kim
	Hyun Jung Park

Powered by Editorial Manager® and ProduXion Manager® from Aries Systems Corporation

Order of Authors Secondary Information:	
Additional Information:	
Question	Response
Are you submitting this manuscript to a special series or article collection?	No
**Experimental design and statistics**<br><br>Full details of the experimental design and statistical methods used should be given in the Methods section, as detailed in our Minimum Standards Reporting Checklist. Information essential to interpreting the data presented should be made available in the figure legends.<br><br>Have you included all the information requested in your manuscript?	Yes
**Resources**<br><br>A description of all resources used, including antibodies, cell lines, animals and software tools, with enough information to allow them to be uniquely identified, should be included in the Methods section. Authors are strongly encouraged to cite Research Resource Identifiers (RRIDs) for antibodies, model organisms and tools, where possible.<br><br>Have you included the information requested as detailed in our Minimum Standards Reporting Checklist?	Yes
**Availability of data and materials**<br><br>All datasets and code on which the conclusions of the paper rely must be either included in your submission or deposited in publicly available repositories (where available and ethically appropriate), referencing such data using a unique identifier in the references and in the "Availability of Data and Materials" section of your manuscript.	Yes

Have you have met the above requirement as detailed in our Minimum Standards Reporting Checklist?	

Manuscript
Click here to access/download;Manuscript;ver25_GIGA_updated.docx

Deep neural networks with knockoff features identify nonlinear causal relations and estimate effect sizes in complex biological systems.

Zhenjiang Fan[1], Kate F. Kernan[2], Panayiotis V. Benos[3], Scott W. Canna[4], Joseph A. Carcillo[2], Soyeon Kim[5,6,*], and Hyun Jung Park[7*]

[1] Department of Computer Science, University of Pittsburgh, Pittsburgh, Pennsylvania, 15213, United States

[2] Division of Pediatric Critical Care Medicine, Department of Critical Care Medicine, Children's Hospital of Pittsburgh, Center for Critical Care Nephrology and Clinical Research Investigation and Systems Modeling of Acute Illness Center, University of Pittsburgh, Pittsburgh, Pennsylvania, 15260, United States

[3] Department of Computational and Systems Biology, University of Pittsburgh, Pittsburgh, Pennsylvania, 15213, United States

[4] Pediatric Rheumatology, The Children's Hospital of Philadelphia, Philadelphia, Pennsylvania, 19104, United States

[5] Division of Pediatric Pulmonary Medicine, Children's Hospital of Pittsburgh, Pittsburgh, Pennsylvania, 15224, United States

[6] Department of Pediatrics, School of Medicine, University of Pittsburgh, Pittsburgh, PA, United States

[7] Department of Human Genetics, University of Pittsburgh, Pittsburgh, Pennsylvania, 15213, United States

zhf16@pitt.edu; kate.kernan@chp.edu; benosapp@pitt.edu; cannas@chop.edu; carcilloja@ccm.upmc.edu; soyeon.kim21@chp.edu; hyp15@pitt.edu

*Correspondence address. Hyun Jung Park, Department of Human Genetics, University of Pittsburgh, Pittsburgh, Pennsylvania, 15213, United States; Email: hyp15@pitt.edu

Abstract

Background: Learning the causal structure helps identify risk factors, disease mechanisms, and candidate therapeutics for complex diseases [1]–[3]. However, although complex biological systems are characterized by non-linear associations, existing bioinformatic methods of causal inference cannot identify the nonlinear relationships and estimate their effect size [4]–[8].

Results: To overcome these limitations, we developed the first computational method that learns both linear and nonlinear causal relations and estimates the effect size using a deep-neural network approach coupled with the knockoff framework [9], named causal Directed Acyclic Graphs using deep-learning VAriable SElection (DAG-deepVASE). Using simulation data of diverse scenarios and identifying known and novel causal relations in molecular and clinical data

of various diseases, we demonstrated that DAG-deepVASE consistently outperforms existing methods in identifying true and known causal relations. In the analyses, we also illustrate how identifying nonlinear causal relations and estimating their effect size help understand the complex disease pathobiology, which is not possible using other methods. **Conclusions:** With these advantages, the application of DAG-deepVASE can help identify driver genes and therapeutic agents in biomedical studies and clinical trials.

Keywords

Causal inference, deep neural networks, effect size estimation.

Background

Since molecular and clinical variables interact for the development of complex diseases such as cancer, asthma, and sepsis [1]–[3], learning the causal structure among the variables helps identify risk factors, disease mechanisms, and candidate therapeutics for the complex diseases for future evaluation. For example, if an abnormal expression of a certain gene modifies the expression level of other genes and contributes to the development of a disease, then controlling this gene can lead to the effective treatment of the disease.

A popular statistical model for causal inference is the causal directed acyclic graph (DAG), which learns conditional dependence among variables[8], [10], [11] because the conditional dependence can further imply the causal relationships under three causal assumptions: Markov, faithfulness, and sufficiency. The causal Markov condition states that causal relationships among the set of variables in their probability distributions (e.g. Bayesian network) are conditionally independent of their non-descendants given their parents[12]. The causal faithfulness condition states that all independence relations in the data are consequences of the Causal Markov condition. The causal sufficiency condition states that input data measured all the common causes of the measured variables, thus no latent (unobserved) confounder exists. Since the

assumptions are not usually met in data, statistical causal inference is limited to identifying causal relationships that are Markov equivalent, which hold the same adjacencies and imply the same independence and conditional independence relationships on the same variables (v-structure). Under the assumptions, bioinformatic methods have incorporated two main approaches to building DAGs: score-based or constraint-based[13]–[20]. Score-based algorithms generally formulate the causal learning problem as optimizing a score function such as Bayesian Information Criteria (BIC) score[14], [21]. Recently, the degenerate Gaussian score (DG) was proposed [22] by extending the BIC score for mixed types of data. Specifically, by embedding discrete variables into a continuous space using one-hot vector representations, DG demonstrates a near-perfect performance under certain simulation scenarios of high-dimensional data. The other approach is constraint-based algorithms to learn constraints that restrict the set of possible causal graphs by testing conditional independence in the input data. Peter and Clark (PC) [13] is among the most popular algorithms under this category that has been applied to diverse biomedical research questions with various extensions [23]–[28]. While PC was initially developed to infer causalities given associations, a recent development, causalMGM (causal mixed graphical model)[29], first identifies associations using MGM and then infers causality of the associations through PC. This two-stage approach showed good scalability and accuracy for high-dimensional simulated and biological data of mixed types [29].

In developing statistical and computational methods, the following properties are critical to identifying causality among high-dimensional molecular and clinical variables in complex diseases. First, a method should identify both linear and non-linear associations. While linear associations may exist, complex biological systems are characterized by non-linear associations [30], [31]. For example, the effects of hormone receptor status on breast cancer biology are often nonlinear due to their complex interactions with other molecular complexes in multiple regulation processes[32]–[34]. Second, a method should estimate the effect sizes of each association. This is critical to facilitating a translatable understanding of the causal relationships since it is important to select a limited number of the most significant causal relationships for downstream

experiments or clinical trials due to both technical and practical limitations. However, current methods in both approaches cannot identify nonlinear associations and identify only linear associations, e.g., a likelihood function only with linear interaction terms[4]–[8]. To address these limitations and enable a more realistic and translatable causal structure learning for complex diseases, we developed the first computational method that learns both linear and nonlinear causal relationships, named causal Directed Acyclic Graphs using deep-learning VAriable SElection (DAG-deepVASE). To identify nonlinear causal relationships in high-dimensional data, DAG-deepVASE incorporated a two-step approach: 1) identify associations and estimate their effect sizes and 2) infer the causality among the associations. In the first step, to identify nonlinear associations, DAG-deepVASE incorporates a deep neural network (DNN) model that employs multiple layers of nonlinear activators. Since a regular DNN model cannot estimate the nonlinear effect size due to its black-box property, DAG-deepVASE incorporated the knockoff framework into the DNN model to estimate the effect sizes. While the knockoff framework has been recently coupled with a deep-learning approach to estimate the effect size for a variable selection context[9], [35], DAG-deepVASE extends this approach for causal inference for the first time. Further, to learn the causal direction for the identified nonlinear associations, DAG-deepVASE extends a score-based approach, DG. While it was not known which causal inference approach would learn the causal direction of nonlinear associations, we conducted extensive studies to find that its asymptotic properties make the inference tractable and flexible enough to learn nonlinear causalities.

DAG-deepVASE consistently outperforms other methods in identifying true causal relations in simulation data of diverse scenarios and identifying known and novel causal relations in molecular and clinical data of various diseases (pediatric sepsis, gut bacteria/nutrient intake and BMI, and breast cancer), facilitating a systematic understanding of the complex disease pathobiology. In the analyses, we also illustrate how identifying nonlinear causal relations and

estimating their effect size help understand the complex disease pathobiology, which is not possible using other methods.

Findings

<u>D</u>irected <u>a</u>cyclic <u>g</u>raph using <u>deep</u>-learning-based <u>va</u>riable <u>se</u>lection (DAG-deepVASE)

We provide here a brief overview of DAG-deepVASE that aims to identify linearly and nonlinearly associated variables while estimating their effect sizes (**Figure 1B, C, respectively**) and learn their causal directions (**Figure 1D**) to produce a DAG from data matrix *X* consisting of *M* input variables (**Figure 1A**). In the first step, to identify linearly associated variables, DAG-deepVASE develops a penalized regression function with the interaction terms connecting the variables and maximizes the likelihood score with sparsity penalties (Methods, **Figure 1B**). While the linear associations have been the main focus of previous causal inference methods[5], DAG-deepVASE further identifies nonlinearly associated variables using a deep neural network (DNN) approach. Although a DNN approach enables to identify nonlinear associations by putting multiple perceptron layers between input variables and the outcome variable, its black-box property makes it difficult to estimate the effect size. Since estimating the effect size is important to ensure a clinical relevance of the associations, DAG-deepVASE successfully estimates the effect size of the nonlinear associations by embedding the knockoff variables in the DNN model (**Figure 1B**). Knockoff variables are a synthetic and noisy copy of the input variables, which resemble the correlation structure of the input variables, but are conditionally independent of the outcome, given the input variables. This property of knockoff variables allows us to estimate how important the original association is in reference to the knockoff variables, leading to the effect size estimation. Although this idea has recently been employed in a variable selection framework, Deep feature selection

using Paired-Input Nonlinear Knockoffs (deepPINK) [36], DAG-deepVASE extends this approach for causal inference for the first time.

In the second step, after identifying both linear and non-linear associations, DAG-deepVASE determines their causal direction using a single metric to ensure causal inference consistency between linear and nonlinear causalities. Since this is one of the first methods that identify nonlinear causal directions, it is unknown whether PC or DG would work better to identify nonlinear causal directions. Among various measures, we chose to use DG because it is accurate, decomposable, and flexible. While its accuracy, which was demonstrated in simulations[22], is clearly beneficial to learning accurate causal directions, we separately conducted an extensive study to find that its decomposability and flexibility were critical to identifying nonlinear causal directions. DG decomposes the task of identifying the optimal causal structure into determining the causal direction of each association. Whereas PC determines the optimal causal structure by considering all associations simultaneously, decomposability allows us to determine the causal direction of each nonlinear association without referring to other associations, making each causal inference tractable. DG also shows flexibility in learning the causal structure generated outside of its model class (conditional Gaussian model). This flexibility allows us to extend DG to learn nonlinear causal directions. In simulation data of diverse scenarios and biological data of various contexts, we demonstrate that DAG-deepVASE can learn causal relations up to the Markov equivalence classes of the true causal relationship.

DAG-deepVASE improves power in identifying nonlinear causal relations in simulation data

To evaluate the performance of DAG-deepVASE in the presence of multiple causal variables, we compared DAG-deepVASE with competing methods on simulation data. We compared DAG-deepVASE with two established methods, causalMGM and DG that employ the two-step strategy as DAG-deepVASE: identifying variable associations and then learning the causal direction of the associations. While causalMGM was originally developed with the two-step strategy, DG does not have the first step because DG is developed to learn causality based on given associations. To be

fair to DG, we developed the first step for DG: in the first step, we applied MGM to identify associations and in the second step, we used the original DG to learn their causalities. We will refer to this model as linear-DG model since the MGM implementation identifies variable associations based on the linear interaction terms. Also, note that whereas DAG-deepVASE identifies both linear and nonlinear associations and uses DG to learn their causal directions, linear-DG identifies linear associations and uses DG to learn their causal directions, and causalMGM identifies only linear associations and applies PC to learn their causal directions. We ran the methods using default parameters or those suggested by the authors throughout this paper (**Table 2**).

To compare the methods in sensitivity and specificity simultaneously, we simulated 10 data sets of 40 or 100 variables where half (20 or 50, respectively) of the variables collectively determine the outcome (true associations) and the other half are not associated with the outcome (false associations, see Methods). Each data set was simulated for 10,000 samples. To mimic biological variables that would interact in various degrees of nonlinearity, simulations were conducted under two scenarios: complete-nonlinear or partial-nonlinear scenarios. We ran DAG-deepVASE and causalMGM on the datasets. We did not run linear-DG since it identifies the same association pairs as causalMGM. In both complete- and partial-nonlinear scenarios, DAG-deepVASE consistently outperformed causalMGM in AUC (area under the receiver operating characteristic curve). Specifically, for the simulations with 40 and 100 associations under the complete-nonlinear scenario, DAG-deepVASE achieves an average of 0.84 and 0.82 AUC, respectively, outperforming causalMGM which achieves an average of 0.71 and 0.68 AUC (**Fig. 2A** and **Fig. 2B**, respectively). The same trend is observed under the partial-nonlinear scenario where DAG-deepVASE achieves an average of 0.84 and 0.83 AUC and causalMGM achieves an average of 0.73 and 0.71 AUC for the simulations with 40 and 100 associations (**S. Fig. 2A** and **S. Fig. 2B**, respectively).

To further mimic biological situations where true associations would be relatively rare among all pairwise combinations of biological variables, we simulated different numbers of variables (M = 50, 100, 200, 400, 600, 800, 1000, 1500, 2000, 2500, and 3000) with various sample sizes (N=200, 600, 1000), where ten variables collectively determine the outcome (true

associations). For each combination of variable number and sample size, we conducted the simulation experiment 10 times. In the complete-nonlinear simulation scenario, we first compared the number of true associations identified by each method before assessing the causal directions. DAG-deepVASE outperforms the other methods by identifying more than 90% of the true associations in most simulation scenarios, whereas causalMGM and linear-DG identified less than half of the associations (**Fig. 2C**). Second, we compared the number of true and false causal directions learned from the identified associations (**Fig. 2D,** respectively**, S. Table 1**). In all experiments under the complete-nonlinear scenario, DAG-deepVASE consistently outperforms the other methods in identifying true causalities. Especially, for larger sample sizes (n=600 and 1,000), DAG-deepVASE identified more than 97% of the true causalities. causalMGM returned bidirectional causal directions for all identified associations, which are counted as both true and false positives. On the other hand, although linear-DG identified less than half of the true associations as mentioned above, it learned the true causalities on the small number of the identified associations (**Fig. 2D**), demonstrating that DG can be used to learn nonlinear causalities. Together with such high true positive rates, DAG-deepVASE also outperforms the other methods by not identifying any false casualties in any of the scenarios, whereas causalMGM returns 3~5 false-positive causalities by returning bidirectional causalities (**Fig. 2E, S. Fig. 1D**). In the partial-nonlinear scenario, a very similar result is returned for power (**S. Fig. 1C)**, true positive causalities (**S. Fig. 1D)**, and false positive causalities (**S. Fig. 1E**).

Altogether, DAG-deepVASE outperforms the other methods by identifying the highest number of true nonlinear associations and by learning the highest number of true causalities across various simulation scenarios without false positives, while competing methods could identify less than half of true nonlinear causalities with several false positives.

DAG-deepVASE identifies both linear and nonlinear associations among clinical features with high sensitivity in pediatric sepsis data.

To demonstrate the importance of identifying nonlinear variable associations for sensitive causal inference, we first focus on identifying associations among diverse types of variables in clinical data. The data consists of clinical and biomarker variables (laboratory parameters, cytokines, and chemokine measurements) from 404 children with severe sepsis[37]. We compared DAG-deepVASE and causalMGM in this section. We excluded linear-DG because they identify the same set of associations with causalMGM. Since DAG-deepVASE assumes that the variables follow the Gaussian distribution, we consider 45 continuous or ordinal categorical variables excluding one binary/nominal variable in the data set. Among the variables, DAG-deepVASE identifies 118 associations (**Figure 3A**), whereas causalMGM identifies 42 associations (49.5%, **S. Table 2**) of the associations. Since causalMGM is only able to identify linear associations, the 42 associations are likely linear. Many of the identified linear associations are already clinically and biologically verified. For example, the serum level of soluble CD163 (sCD163), a macrophage activator[38], only has linear associations (**Figure 3B**) with biomarkers known to activate macrophages, such as M-CSF (macrophage colony-stimulating factor)[39], MCP-1 (monocyte chemoattractant protein-1)[40], Il-1b[41], TNF-a[42], [43] and other key drivers of macrophage response including its ligand hemoglobin[44]. Also, age is another variable only linearly associated with other variables, including heart rate, creatinine, and lymphocyte count (**Figure 3B**). Since each of them changes monotonically with age in pediatric subjects [45]–[47], it is reasonable that they are identified as linear associations.

In addition to the 42 linear associations that are identified by both DAG-deepVASE and causalMGM, DAG-deepVASE uniquely identifies 76 nonlinear associations. Multiple nonlinear associations have been validated in previous clinical and biological studies with an implication for nonlinearity. An example is an association between systemic inflammatory response syndrome (SIRS) status and heart rate (**Figure 3B**). This association is expected as nonlinear, as the SIRS status is diagnosed by a nonlinear combination, which is the presence of any two of the four clinical

criteria, including tachycardia (elevated heart rate) [48]. Also, as the SIRS response is defined as a result of systemic immunological activation, DAG-deepVASE uniquely found nonlinear associations between SIRS status and pro-inflammatory cytokines including CRP[49], [50], IL-1β [51], and IFN-γ [52] (**Figure 3B**), corroborating their collective roles in inflammation. Since cytokines are produced involving different combinations of signal transduction pathways[53], [54], their associations with SIRS are expected to be nonlinear rather than linear.

While the method identified validated associations, DAG-deepVASE also identified novel nonlinear relationships of clinical potential for future validation. For example, it identified the nonlinear associations between central nervous system (CNS) dysfunction and SIRS and between IL-22 and SIRS (**Figure 3B**). The former is validated: critically ill patients with SIRS are known to have a measurable risk for organ dysfunction such as CNS dysfunction [55], [56]. This validation also confirms our causal inference that found the causal direction from SIRS status to CNS dysfunction (**S. Table 2**). As it is imperative to elucidate how SIRS interacts with modifiable cytokines for clinical potential, our causal inference also suggests the novel clinical potential of IL-22 to treat CNS dysfunction through modifying SIRS status. While IL-22 plays a key role in immunoregulation and has been linked to the development of organ failure in mouse models of abdominal sepsis [57], DAG-deepVASE revealed the causal relationship from IL-22 to SIRS status in children with sepsis. Especially, it identified this relationship by strong effect size (top 19th out of 118, **S. Table 2**), suggesting a strong reproducibility and thus clinical utility. After more experimental validations, this result can help design future clinical trials to treat organ dysfunctions with IL-22 for pediatric sepsis. Altogether, DAG-deepVASE identifies both validated and novel findings by identifying linear and nonlinear associations with high sensitivity.

DAG-deepVASE accurately identifies nonlinear causalities and estimates their effect sizes in the nutrients/gut bacteria and body-mass index (BMI) data

Variables in complex biological systems interact in varying degrees of nonlinearity [58]–[60]. To examine the sensitivity of DAG-deepVASE in the presence of various degrees of nonlinearity, we compared DAG-deepVASE with causalMGM and linear-DG on a cross-sectional data set consisting of 214 nutrient intakes, 87 gastrointestinal (GI) bacteria genera and body-mass index (BMI) collected from 90 healthy volunteers[61]. Note that nutrient intakes would affect GI bacteria before affecting BMI, suggesting generally a more nonlinear relationship between the nutrient intakes and BMI than between them and GI bacteria. For a balanced assessment, we selected the same number (8) of nutrient intakes and bacteria genera that are known to affect BMI in animal experiments or clinical trials out of the 214 nutrient intakes and 87 bacteria genera data (**Table 3**). DAG-deepVASE identified all 16 associations, while causalMGM and linear-DG identified only 5 associations (31.3%): all these 5 associations are between specific GI bacteria and BMI (**Figure 4A**). Note that causalMGM and linear-DG fail to identify any association between nutrient intakes and BMI, while DAG-deepVASE could identify all 8 of them. Since nutrient intakes likely affect BMI more nonlinearly than between GI bacteria and BMI, this result reaffirms that DAG-deepVASE uniquely identifies nonlinear relationships. To characterize the nonlinear associations, we examined how the 8 nutrient intake and 8 bacteria genera levels change against the BMI value. The 5 associations between GI bacteria and BMI identified by all three methods show a single linear association throughout the BMI region (**Figure 4B** (p-value for linear fit: 0.001)**, S. Figure 2K-O**). On the other hand, the other 8 associations between nutrient intakes and BMI and 3 associations between GI bacteria and BMI, which are identified by only DAG-deepVASE, show nonlinear relationships (**Figure** 4C (p-value for linear fit: 0.58), **S. Figure 2A-J** (p-value for linear fit on average 0.42)), characterized by multiple sub-trends across the BMI ranges. For example,

choline and phosphatidylcholine w/o suppl. intake (**Figure 4C**) shows an increasing trend from BMI 1~3, a decreasing trend from BMI 3~4, then another increasing trend from BMI 4~5.

In the second step of determining causalities from the identified associations, we deemed true the causal directions from nutrient intake/ bacteria genera to BMI based on literature in **Table 2**. DAG-deepVASE identified all 16 true causal directions. On the other hand, as causalMGM uses PC to learn the causal directions of the associations, PC removed 2 of the 5 associations in its step of testing the conditional independence relationship and identified the other 3 associations as bidirectional causalities that we considered to be both false positive and false negative (**Figure 4D**). While linear-DG identified 5 true causal directions on the five identified associations, it still could not learn 11 causalities because of its inability to identify nonlinear causality. Altogether, DAG-deepVASE outperforms other methods due to its ability to identify nonlinear associations combined with the excellent performance of DG in learning nonlinear causalities among the identified associations.

Further, to demonstrate how the effect size DAG-deepVASE estimates leads to the unbiased discovery of causal relations, we estimated the effect size of all 301 potential associations between nutrient intake/bacteria genera and BMI using DAG-deepVASE including non-validated ones (**S. Table 3**). The top 5 associations that have the largest effect sizes estimated by DAG-deepVASE's nonlinear module are all validated: Meganomas[62], Phenylalanine[63], Mitsuokella[64], Parvimonas[63] and Sporobacter [64]. Since these findings were independent, it is difficult to prioritize their importance. To conduct further experimental validations or clinical trials that target a limited number of strong associations, estimating effect sizes via DAG-deepVASE

enables to prioritize important variables. Also, while the 5 top features are already validated, it would be interesting to validate other novel features with large effect sizes.

DAG-deepVASE identifies causal relations among molecular and clinical variables in breast cancer data

Among various types of variable interactions in a complex disease, identifying causal relationships between molecular variables (e.g., gene expression) and clinical variables (e.g., cytokine measurements in the serum) are particularly interesting because the findings can help identify molecular therapeutics. To evaluate the performance of DAG-deepVASE in learning the complex molecular pathogenic mechanisms, we compared DAG-deepVASE and the other methods on the TCGA breast cancer of gene expression and clinical variables, such as PAM50 (n=601 tumor samples). PAM50 is an important clinical feature to categorize breast tumors, which is defined by the tumor's expression of 50 genes [65]. Therefore, we consider the causal directions from the genes to the PAM50 status as true positives. For our analysis, we chose 10 of the 50 genes (PAM50-defining genes) that are also included in the top 500 genes that have the highest variance across the samples (high variance gene set). In addition to the 10 genes, we also considered 5 clinical variables, which are known to characterize breast tumors with the PAM50 status: estrogen receptor (ER)[66], progesterone receptor (PER)[66], human epidermal growth factor receptor (HER)[67], lymph node status[68], and tumor staging code[69].

In the first step of identifying associations between the 10 PAM50-defining genes with the highest variances and PAM50 status, DAG-deepVASE identified 9 associations out of 10 associations, while causalMGM and linear-DG identified only 5 of them, attributing the 40% power increase of DAG-deepVASE to the identification of nonlinear associations. Between the 5 clinical variables and PAM50, DAG-deepVASE identified all 5 associations while causalMGM and linear-DG identified only one association (20%) (**Figure 5A, S. Figure 3A, B**), suggesting that 80% (4 of 5) of the associations are nonlinear. In the second step of learning causal directions, DAG-deepVASE outperforms both causalMGM and linear-DG, identifying true causalities from all 9

identified associations between the genes and PAM50 (**Fig. 5B**). On the other hand, causalMGM identified bidirectional causalities for 3 associations after the PC step removed the other two associations based on the conditional independence relationships. And linear-DG identified the correct causalities on all 5 identified associations but still missing causalities for the other 5 associations that linear-DG could not identify. We did not assess the causal directions between the 5 clinical variables and PAM50 since the true causal directions are not clear between them. We tried different parameter settings of the methods to find that this trend holds true across the parameter settings (**S. Fig. 3C, S. Table 4**).

To demonstrate how DAG-deepVASE enables us to understand complex pathogenic mechanisms across multiple regulatory layers in breast cancer, we expanded our analysis by investigating causalities among the 10 genes and the 6 clinical features, including the PAM50 status. Specifically, we inspected whether it is linear or nonlinear causalities in the following categories: causalities between a gene and a clinical feature and causalities between clinical features. First, while only a few causalities between genes were identified as non-linear interactions (2 of 8 (25%)), most of the causalities between clinical features and between a clinical feature and a gene were identified as nonlinear causalities (13 out of 15 (86.7%) and 34 out of 43 (79.1%) respectively). While many of them are previously validated in clinical trials or biological experiments, e.g., from ERBB2 (HER2) to PR (progesterone receptor), ERBB2 to ER (estrogen receptor), and ERBB2 to PAM50 [70]; KRT5 (keratin5) to PR and KRT5 to ER[71] (**Figure. 5D**), the prevalence of nonlinear causality is consistent with the expectation since the clinical features, mostly hormone receptor status, are regulated through multiple biochemical pathways[72], and thus these relations are likely nonlinear. Other studies also advocate the nonlinear interactions of the clinical features by showing that incorporating nonlinearity in statistical models improves the prediction accuracy of their effects on breast tumor biology, e.g., in the transcriptional profile and survival analysis[32]–[34]. Second, between a gene and a clinical feature, the method found that all 43 causal directions are from genes to clinical features (**Figure. 5C**). Since the clinical features in this data are mainly hormone receptor status, this result conforms to the expectation that genes

code for the hormone receptor activity[73]. Incorporating all linear and nonlinear causalities under the categories sheds mechanistic insight into the complex tumorigenic process underlying breast cancer. For example, although the keratin genes (KRT5, KRT14, and KRT17) were found to interact in cancer genome studies[74]–[76], it was not clear how the cluster affects clinical features for cancer. Our result suggests that it is because the gene cluster is formed in linear interactions but its effect on clinical features is mostly nonlinear. Altogether, DAG-deepVASE could identify nonlinear causalities, consisting of 74.2% of all causalities in this data, which would be missed by other existing methods. Identifying nonlinear causal relationships sheds insights into not only genetic interactions but also their interactions with clinical features of tumor biology.

Discussion

We developed the first method, DAG-deepVASE, to learn both linear and nonlinear causal relationships in complex biological systems in high-dimensional molecular and clinical data. In complex biological systems, multiple regulatory layers, e.g., transcriptome and methylation layers, extensively interact[1], [3], [77], [78] and render variable interactions highly nonlinear. In the simulated data of diverse scenarios and biological data of various contexts (pediatric sepsis, TCGA breast cancer, BMI with nutrients and gut bacteria), DAG-deepVASE consistently outperforms existing methods in identifying known and new nonlinear causal relations. In the first step to identify associations, while DAG-deepVASE identifies all the linear associations that are identified by causalMGM and linear-DG, the method identifies non-linear associations through DNN, which shows the power ranging from 87 % to 100 % in identifying associations. In the second step to identify causalities from the identified associations, DAG-deepVASE infers causalities with almost perfect accuracy (ranging from 88 % to 100 %) while causalMGM learned bidirectional causalities on most of the associations and linear-DG learned only a small number of identified associations correctly. The reason why causalMGM learned bidirectional causalities in

our analyses is that it returns a bidirectional causality between variables mediated or confounded by latent variables [8], which very likely exist in molecular and clinical data sets. In contrast, the second step in DAG-deepVASE imposes a model on input variables (x_j) instead of on the conditional distribution of the association (distribution of $x_j|x_i$). This imposition guarantees to identify nonlinear associations even when the model for the association is mis-specified due to absence of all latent mediating variables.

Another advantage of DAG-deepVASE is the knockoff framework to estimate effect size for nonlinear associations that prioritizes causal relations over simple correlation based on the exchangeability property of the knockoff framework (see Method). The estimated effect size is significantly larger for validated causal relationships than for non-validated ones in both the BMI data (P-value=0.02, **S. Fig. 2P**) and the breast cancer data (P-value=0.03, **S. Fig. 3D**). Based on the rationale that the effect sizes of validated associations are more apparent and thus stronger, these results suggest that the effect sizes estimated by DAG-deepVASE make sense, and these can facilitate translatable findings of the causal relationships by selecting strong causal relations, either linear or nonlinear, to test in downstream experiments or clinical trials.

DAG-deepVASE enables a further translatable understanding of complex diseases by putting linear and nonlinear associations together. In the subnetwork of pediatric sepsis data presented above, IFN-γ and TNFα are connected through linear and nonlinear associations (gray nodes in **Fig. 3B**). Mouse experiments showed that the interaction between IFN-γ and TNFα triggers inflammatory cell death, tissue damage, and mortality in acute immune diseases characterized by “cytokine storm” including lipopolysaccharide (LPS)-mediated sepsis [79]. While it is difficult to identify multiple cytokines involved in the complex interactions, DAG-deepVASE could identify the interactions between IFN-γ and TNFα via multiple associations of both linear and nonlinear ones, including MCP-1/CCL2. Since MCP-1/CCL2 shows a protective role in a similar mouse model (a polymicrobial sepsis model with LPS) [80], DAG-deepVASE suggests a therapeutic potential to the detrimental interaction between IFN-γ and TNFα.

Despite the clear advantages, DAG-deepVASE has some limitations in improving the clinical relevance of the findings. The first is that DAG-deepVASE cannot take nonordinal categorical variables and take only continuous and ordinal categorical variables that approximately follow Gaussian distribution since model-X knockoff assumes Gaussian distribution. In this paper, this condition did not pose any problem as all variables of our interest were either continuous or ordinal categorical. However, in the future, we will generate the knockoff variables for nonordinal categorical variables based on a regression model for nonordinal categorical variables [81]. Second, while DAG-deepVASE can estimate the effect size, it does not estimate statistical significance. Thus, to identify significant causal relations in the future, we will estimate statistical significance of the likelihood ratio test we derived to determine the causal direction in (1). Third, as with other methods of learning causalities from observational data, the validity of the learned causalities depends on how well the data comply with the three causal assumptions: Markov, faithfulness, and sufficiency. However, biological data could violate these assumptions and weaken the applicability of the inference results. For example, since multiple biological layers, such as genomic, transcriptomic, and epigenetic layers, often interact to render a phenotype in humans, confounders can occur in any of the layers. However, it is not always feasible to measure all variables from all the layers due to technical and practical reasons, indicating that the causal sufficiency assumption of no latent confounder would be hardly met for biological data. Thus, it is necessary to conduct further experiments or clinical trials to validate the causal relationship learned through DAG-deepVASE.

In summary, we developed DAG-deepVASE, which learns causal relationships in complex biological systems. DAG-deepVASE is the first method that uses a DNN approach to identify linear and nonlinear associations and learn their causal directions. DAG-deepVASE outperforms existing methods, causalMGM, and linear-DG, in identifying known causal relations in various simulation scenarios and molecular and clinical data sets. In addition to known causalities, DAG-deepVASE identifies novel complex pathobiological interactions involving nonlinear causal relations, which is not possible using other methods. By applying the knockoff

framework to DNN, DAG-deepVASE estimates effect size for nonlinear associations that prioritizes causal relations, which allows to prioritize future clinical and experimental validations. With these advantages, the application of DAG-deepVASE can help identify driver genes and therapeutic agents in biomedical studies and clinical trials.

Methods

In developing our method, we followed the DOME (Data, Optimization, Model, and Evaluation) guidelines stated in https://dome-ml.org/. Especially, we selected testing data that is representative of the domain (TCGA breast cancer for molecular data, gut microbiome and obesity data for metagenomics, and pediatric sepsis data for clinical data) per the Data guidelines. Their accessions are further detailed in **Availability of Supporting Data** section below. Per the Optimization guidelines, we reported all hyperparameters (**Table 2**) and optimization protocol under **Running parameters of DAG-deepVASE** section below. Per the Model guidelines, we dockerized our method to make it easier for people to test and deploy. Lastly, per the Evaluation guidelines, we compare our method both with public method (causalMGM) and simple (baseline) method (linear-DG) on the same dataset.

Availability of Supporting Data

We used a simulation data set, two public data, and one access-controlled data. Our simulation data are downloadable from our project website (https://github.com/ZhenjiangFan/DAG-deepVASE). TCGA breast invasive carcinoma (BRCA) data were downloaded from https://tcga.xenahubs.net, available under BRCA cohort, under gene expression RNAseq section, on IlluminaHiSeq (n=1,218) TCGA Hub. It consists of the gene expression RNAseq dataset (dataset ID: TCGA.BRCA.sampleMap/HiSeqV2) and the clinical phenotype dataset (dataset ID: TCGA.BRCA.sampleMap/BRCA_clinicalMatrix). To investigate the dietary effect of the human gut microbiome, we downloaded a cross-sectional data of 98 healthy volunteers from https://noble.gs.washington.edu/proj/DeepPINK/ that combined data sets from three studies[82]–

[84]. We also used an access-controlled data of pediatric sepsis. Data are available upon request and after taking due steps for the rights and welfare of human research subjects involved in the study (regarding the Institutional Review Board review). Details of each data are given below.

Breast cancer data

For the gene expression dataset, we selected 500 expressed genes based on their variances and added ERBB2 (also known as HER2 or *neu*) to the selected gene set. ERBB2 was included due to its important role in human malignancies, especially for human breast cancers[85]. For the clinical dataset, we used 10 well-known clinical status features: PAM50 status (PAM50Call_RNAseq), HER2 status (HER2_Final_Status_nature2012), tumor stage (Converted_Stage_nature2012), tumor node status (Node_nature2012), the progesterone receptor status (breast_carcinoma_progesterone_receptor_status), the estrogen receptor status (breast_carcinoma_estrogen_receptor_status), the number of lymph nodes (lymph_node_examined_count), neoplasm cancer status (person_neoplasm_cancer_status), pathologic stage information (pathologic_stage). Our analysis was based on 601 samples without missing values in the 10 selected features.

Gut microbiome data

This data has 214 micronutrients and 87 genera from 90 healthy donors. The BMI data were evaluated based on the donors' information and the bacteria data are extracted using 16S rRNA sequencing from the stool samples. For a consistent result with previous analyses, we used the same data pre-processing procedure as previous computational work on the data[36]. In particular, the nutrient values are normalized using the residual method to adjust for caloric intake and then standardized [82]. Then, this data is log-ratio transformed to get rid of the sum-to-one constraint and then centralized. Following [83], 0s are replaced with 0.5 before converting the data to a compositional form. With both the nutrient intake and genera composition as predictors, we treat BMI as the response.

Pediatric sepsis data

The pediatric sepsis data were collected from 9 PICUs in the Eunice Kennedy Shriver National Institutes of Child Health and Human Development Collaborative Pediatric Critical Care Research Network (including Children's Hospital of Pittsburgh, Children's Hospital of Philadelphia, Children's National Medical Center, Children's Hospital of Michigan, Nationwide Children's Hospital, Children's Hospital of Los Angeles, St. Louis Children's Hospital, C. S. Mott Children's Hospital, and Mattel Children's Hospital at the University of California Los Angeles) [86]. Briefly, we collected blood samples and clinical data obtained from our previously published PHENOMS study[86]. Approval was obtained from The University of Utah Institutional Review Board, Central IRB # 70976. Written informed consent was obtained from one or more parents/guardians for each child. Assent was garnered when the child was able. Patients were enrolled from 2015 to 2017. The CONSORT diagram and details of the clinical study protocol have been previously published [86]. In brief, children qualified for enrollment in PHENOMS if they 1) were between the ages of 44 weeks gestation to 18 years of age; 2) were suspected of having infection meeting two or more of four systemic inflammatory response criteria [87], and 3) had one or more organ failures [88]. Three consented and enrolled children who were excluded from reporting in the parent study manuscript due to a maximum per site enrollment of 81 patients to evenly distribute enrollment among the centers, are additionally included in this analysis. Another work investigating this data is in progress and thus this data is currently not deposited in the public domain yet. There originally were 55 candidate clinical features and 33 cytokine features measured from 404 children admitted. We removed features with a missing rate higher than 20% as well as highly correlated features (Pearson's correlation coefficient > 0.6). Finally, we dropped samples with any missing data. As a result, this dataset provides 56 features (**Table 1)** from 281 samples with low correlations (< 0.3 and > -0.44 in Pearson's correlation coefficient, **Figure R1**). In our analyses, some clinical terms were reported with abbreviations; GCS: Glasgow Coma Scale; CRPH: C-reactive protein; SIRS: Systemic Inflammatory Response Syndrome: sCD163:

soluble CD163; M-CSF: Macrophage colony-stimulating factor. **Table 1** has all the variables with full names.

Availability of supporting source code and requirements

Project name: DAG-deepVASE

Project home page: https://github.com/ZhenjiangFan/DAG-deepVASE

Operating system(s): Platform independent

Programming language: Python, Java, C, and R

Other requirements: e.g., Java 1.3.1 or higher, Tomcat 4.0 or higher

License: MIT license

Pre- and post-processing

To reduce false-positive discoveries, DAG-deepVASE carries out several pre- and post-processing steps. As a pre-processing step, DAG-deepVASE filters out variable pairs that are conditionally independent on all the other variables based on inverse covariance (< 0.0001), using a python function in the package for machine-learning optimization (scipy.linalg.inv). Although it's a common practice for computational causal inference under certain assumptions and the filtered-out nodes may not change the rest of the network, we made it optional since they can be important nodes for downstream analysis [89]. As another optional postprocessing step, DAG-deepVASE can detect a cycle (a non-empty tail in which the first and the last nodes are equal) in the network connecting the causal relations. Further, users can remove the cycle components by removing edges with their prior knowledge or DAG-deepVASE can automatically remove the edges with the least effect size it estimates.

Running parameters of DAG-deepVASE

To identify linear and nonlinear associations in each data set, we first performed pre-processing steps described in the "**Pre- and post-processing**" section below. To identify linear associations

after the steps, we ran Lee and Hastie's log-likelihood model [6] for all possible variable pairs (x, y in **Equation 1**) with the penalty to select important variables (**Equation 2**). We set the sparsity penalty values of the likelihood function to 0.3 unless specified otherwise. Variable pairs remained after applying the penalty are significant linear associations.

To identify nonlinear associations, we first built a deep neural network model consisting of the input layer, two hidden layers, and the output layer (Step 1-1 in **Fig. 1)**. Assume that the input data has *p* input variables, then we set the input layer with 2**p* neurons, since we generated the knockoff variable for each input variable and combine them in a pair-wise fashion in the input layer (**Equation 3, 4)**. The combined input-knockoff neurons are fully connected to the hidden layers. For the case of *p* input variables, each hidden layer has *p* neurons, further transformed using the rectified linear unit activation (ReLU) function[90]. The initial weights for the hidden layer are generated using the Glorot normal initializer[91], which uses $L1-regularization$ with the regularization parameter set to $O(\sqrt{\frac{2logp}{n}})$. To train this model, mean of squares of errors (MSE) is used to calculate the loss in comparison with the response on the output layer. To train the model's parameters with respect to the loss function, we used a stochastic gradient descent method called "Adam optimization". Then, we ran it to identify variables that predict the outcome variable with a high effect size estimated in **Equation 5, 6.** Equations are described in the section of **Algorithm of DAG-deepVASE**. All running parameters for the nonlinear module is summarized in **Table 2**. To identify nonlinear associations between all pairs, we ran this procedure repeatedly with each variable as outcome and all the rest as input. While the procedure was previously developed to identify input variables that can predict the outcome [36], DAG-deepVASE identify these prediction pairs as associated variables based on a widely accepted notion that a predictor and the outcome is statistically an associated pair. For theoretical understanding of the Equations, readers are referred to the following section.

Algorithm of DAG-deepVASE

Let X be the data matrix of interest with variables measured over N observations. $x_i \in X$ is the M -dimensional feature vector observed for sample i, consisting of C continuous variable set X_C and D ordinal categorical variable set X_D $(C + D = M)$. To systematically construct a DAG from both linear and nonlinear associations among variables, DAG-deepVASE leverages a well-established computational framework where variable associations are first identified, and their causal directions are then learned [5], [7], [92].

In the first step, DAG-deepVASE selects linearly associated variables based on Lee and Hastie's log-likelihood [6] as follows.

Equation 1. $logp(X_C, X_D, \Theta) = \sum_k^C \sum_l^C \left(-\frac{1}{2}\beta_{kl} X_{Ck} X_{Cl}\right) + \sum_k^C \alpha_k X_{Ck} + \sum_k^C \sum_l^D v_{kl}(X_{Dl}) X_{Ck} + \sum_k^D \sum_l^D \Phi_{\mathrm{kl}}(X_{Dk}, X_{Dl}) - \log(Z)$,

where Θ represents all of the model parameters, β_{kl} is the interaction coefficient between two continuous variables, X_{Ck} and X_{Cl}, α_k is the potential of continuous variable X_{Ck}, v_{kl} is the interaction parameter between continuous variable X_{Ck} with each index of the categorical variable X_{Dl}, Φ_{kl} is a matrix of interaction parameters between discrete variable X_{Dk} and X_{Dl} (indexed by their levels) [6]. If the data consists only of continuous variables, this model reduces to a multivariate Gaussian model with β_{kl} coefficient as entries in the precision matrix. If only with categorical variables, this model is the popular pairwise Markov random field with potentials given Φ_{kl}. While calculating the partition function Z can be expensive, it is possible to optimize the log-likelihood edge by edge[8] under the faithfulness and causal Markov assumptions. Overall, this equation models the log-likelihood of interactions of continuous variables and categorical variables as a multinomial linear regression. To ensure sparsity and select associated variables in the regression model, Sedgewick et al. introduced sparsity penalties for associations between continuous variables, between a continuous and a categorical variable, and between categorical variables ($\lambda_{cc}, \lambda_{cd}, \lambda_{dd}$, respectively) as follows [8].

Equation 2. $minimize_{\Theta} \tilde{l}(\Theta) + \lambda_{cc} \sum_{i<j} |\beta_{ij}| + \lambda_{cd} \sum_{i,j} \left|\left|v_{ij}\right|\right|_2 + \lambda_{dd} \sum_{i<j} \left|\left|\Phi_{\mathrm{ij}}\right|\right|_F$

For balance estimation of the associations, DAG-deepVASE uses the same sparsity penalty (0.3 for all three interactions) and set FDR level q as 0.05. After selecting the interactions, we report as effect size the coefficients in the model (β_{ij}, v_{ij}, or, Φ_{ij}, corresponding to the type of the selected variables).

In the second step, DAG-deepVASE selects non-linearly associated variables as follows. To identify nonlinear associations with x_i, DAG-deepVASE sets multiple perceptron layers between $X_{\backslash i} = \{x_1, x_2, \ldots, x_{i-1}, x_{i+1}, \ldots, x_M\}$ and x_i (Step 1. Nonlinear association in **Figure 1**) and estimate the effect size of the association between ($x_j \in X_{\backslash i}$, x_i). To estimate the effect size, DAG-deepVASE generates model-X knockoff[93]. For input variables x_i and x_j, the exchangeability property ensures that $(x_i, x_j, \widetilde{x_j}) =^d (x_i, \widetilde{x_j}, x_j)$, where " $=^d$ " denotes equality in distribution. This exchangeability properties help prioritize causal relations with x_i over simple correlations. For example, suppose (x_i, x_k) is a correlation without causal relation. Then, the feature exchangeability $(x_i, x_k, \widetilde{x_k}) =^d (x_i, \widetilde{x_k}, x_k)$ will hold and make their relationship measure $|RI_{ik}|$ and $|\widetilde{RI_{ik}}|$ exchangeable, which will make $S_{ik} = |RI_{ik}| - |\widetilde{RI_{ik}}|$ to follow a distribution symmetric around 0. On the other hand, suppose (x_i, x_j) is a causal relation. Then, S_{ij} will indicate how deviated the relationship of (x_i, x_j) is compared to the null hypothesis, leading to estimation of the effect size. The idea is that knockoff matrix $\tilde{X}$ is generated to mimic the correlation structure within X but minimises the cross-correlation with outcome variable[93]. Specifically, model-X knockoff variables for the set of random variables $X = (x_1, \ldots, x_p)^T$ of our interest are a new family of random variables $\tilde{X} = (\widetilde{x_1}, \ldots, \widetilde{x_p})^T$ that satisfies two properties: (1) $(X, \tilde{X})_{swap(s)} =^d (X, \tilde{X})$ for any subset $S \subset \{1, \ldots, M\}$, where $swap(s)$ means swapping x_j and $\widetilde{x_j}$ for each $j \in S$ and $=^d$ denotes equal in distribution, and (2) $\tilde{X} \perp\!\!\!\perp Y|X$, that is, $\tilde{X}$ is independent of X given outcome Y. Suppose $x_j \sim N(0, \Sigma)$ with $\Sigma \in \mathbb{R}^{M \times M}$ the covariance matrix. A valid construction of $\widetilde{x_j}$ is

Equation 3. $\widetilde{x_j}|x_j \sim N(x_j - diag\{S\}\Sigma^{-1}x_j, 2diag\{S\} - diag\{S\}\Sigma^{-1}diag\{S\})$.

Model-X knockoffs can be sampled from the conditional distribution of $\widetilde{x_i}|x_i$ as follows.

Equation 4. $(x_j, \widetilde{x_j}) \sim N\left(\binom{0}{0}, \begin{pmatrix} \Sigma & \Sigma - diag\{S\} \\ \Sigma - diag\{S\} & \Sigma \end{pmatrix}\right)$

In this sampling, the sensitivity of identifying x_j can increase with a larger S since it will make the knockoffs more different from S, subjected to another constraint that S should make $\Sigma - diag\{s\} \geq 0$. By pairing knockoff variable $\widetilde{x_j}$ with the corresponding input variable x_j and optimizing together for x_i, one can quantify the importance of x_j in reference to $\widetilde{x_j}$. Specifically, let $W_i^{(0)} \in \mathbb{R}^{M\times 1}, W_i^{(1)} \in \mathbb{R}^{M\times M}, W_i^{(2)} \in \mathbb{R}^{M\times M}, and\ W_i^{(3)} \in \mathbb{R}^{M\times 1}$ be the weight matrices connecting the input vector to the first hidden layer, the first hidden layer to the second hidden layer, the second hidden layer to the third hidden layer, and the third hidden layer to x_i, respectively. The weight estimates can be summarized into $w_i = W_i^{(0)} \otimes (W_i^{(1)} W_i^{(2)} W_i^{(3)})$, where $\otimes$ denotes the element-wise matrix operation. Also, let ri_{ji} and $\widetilde{ri_{ji}}$ be the filter weight for x_j and its knockoff counterpart $\widetilde{x_j}$. Then, variable importance values can be estimated for input and knockoff variables as follows.

Equation 5. $RI_{ji} = ri_{ji} \times w_i\ and\ \widetilde{RI_{ji}} = \widetilde{ri_{ji}} \times \widetilde{w_i}$.

We use Adam to train this deep learning model with respect to the mean squared error loss, using an initial learning rate of 0.001 and batch size 10. With $S_{ji} = |RI_{ji}| - |\widetilde{RI_{ji}}|$, DAG-deepVASE estimates effect size on ($x_j \in X_{\setminus i}$, x_i) adopted from [9], [35], which can be described in the following two options:

Equation 6. $T = min\left\{t \in S, \frac{|\{j:S_{ji} \leq -t\}|}{|\{j:S_{ji} \geq t\}|} \leq q\right\} or\ T_+ = min\left\{t \in S, \frac{1+|\{j:S_{ji} \leq -t\}|}{1 \vee |\{j:S_{ji} \geq t\}|} \leq q\right\}$

where q is a user-defined nominal false discovery rate and T or T_+ is a threshold value for determining which features should be selected. We controlled FDR $q = 0.05$ based on S_{ji}. While this setting has previously been used for a variable selection problem with respect to outcome [31], we extend this problem to estimate the nonlinear effect size for associated variables in this manuscript. Since model-X knockoff assumes to follow Gaussian distribution, we will include only continuous or ordinal categorical variables that approximately follow Gaussian distribution (using Q-Q plot). We set parameters of DAG-deepVASE according to a guideline that utilized the knockoff framework for variable selection [36] (**Table 2**).

In the third step, for each identified variable association (x_i, x_j), whether linear or nonlinear, DAG-deepVASE determines the causal direction as extended from the DG framework as follows. calculated as:

Equation 7. $DG(G,Z) = \sum_{j=1}^{p} dg\left(Z_j|Z_{Pa_j^G}\right)$,

where

$dg\left(Z_j|Z_{Pa_j^G}\right) = \ell\left(\hat{\theta}_{mle}|Z_{\{j\}\cup Pa_j^G}\right) - \ell\left(\hat{\theta}_{mle}\middle|Z_{Pa_j^G}\right) - \frac{c}{2}|Z_j|\left|Z_{Pa_j^G}\right|\log(n)$,

where c is a penalty discount used to tune the density of the resulting graph. Also, $\ell(\hat{\theta}_{mle}|Z_{sub})$, which is the log-likelihood of a subset of Z, is computed using the Gaussian log-likelihood function in reference to $\hat{\sum}_{sub}$, the partial covariance matrix for the input variables. Note $dg\left(Z_j|Z_{Pa_j^G}\right) = logP\left(X_j|X_{Pa_j^G}\right)$ if the data has only continuous variables[14]. By maximum likelihood, the DG framework determines x_j as causal and x_i as effect if $dg(x_i|x_j) > dg(x_j|x_i)$ or $dg(x_i|x_j) - dg(x_j|x_i) > 0$. Due to multiplication commutativity,

Equation 8. $dg(x_i|x_j) - dg(x_j|x_i)$

$$= \ell(\hat{\theta}_{mle}|x_{\{i,j\}}) - \ell(\hat{\theta}_{mle}|x_j) - \frac{c}{2}|x_i||x_j|\log(N) - \left(\ell(\hat{\theta}_{mle}|x_{\{j,i\}}) - \ell(\hat{\theta}_{mle}|x_i) - \frac{c}{2}|x_j||x_i|\log(\mathrm{N})\right)$$

$=\ell(\hat{\theta}_{mle}|x_i) - \ell(\hat{\theta}_{mle}|x_j)$

$= l(\frac{\hat{\theta}_{mle}|x_i}{\hat{\theta}_{mle}|x_j})$. (1)

After running this likelihood ratio test, we algorithmically remove the causal relations that create a cycle (a non-empty tail in which the first and the last nodes are equal) to ensure acyclicity by removing the one association with the least effect size (S_{ji}).

Simulation for nonlinear associations

The nonlinear simulation datasets were generated using a single index model [94]–[97]. Each simulation dataset consists of three parts: outcome variable $y = (Y_1, \dots, Y_n)^T \in \mathbb{R}^{N\times 1}$; a set of independently and identically distributed random variables $X \in \mathbb{R}^{N\times M}$ which have a different

degree of nonlinear association with y; and a set of independently and identically distributed random variables $Z \in \mathbb{R}^{Q \times M}$ which have no association with Y. The following model was used to generate associated variable pairs x_i and Y:

Equation 9. $Y_i = \alpha g(x_i^T \beta) + (1 - \alpha) x_i^T \gamma + \varepsilon_i$

where g is a nonlinear link function which we set to be a cube (X^3) function, Y_i is the outcome value and ε_i is noise added to the ith outcome. α determines the proportion of the (non)linearity of the simulation where $\alpha = 1$ determines the association of X_i and Y_i only with the nonlinear link function (complete-nonlinear) and $\alpha = 0.5$ determines the association half by the nonlinear function and half by the linear function (partial-nonlinear). The distribution for noise ε was simulated from $\mathcal{N}(0, \sigma^2 \mathrm{I}_N)$, where σ is set as 1. The rows of X was simulated independently from a distribution $\mathcal{N}(0, \sum)$ with a precision matrix $\sum^{-1} = (\rho^{|j-k|})_{1 \le j,\ k \le (q+p)}$ with $\rho = 0.5$. A similar strategy has been used to assess the performance of deep-learning methods developed for variable selection and causal inference, deepPINK[36], and DAG-GNN[98], respectively. In this manuscript, we extended their methods by diversifying the degree of nonlinearity by adding the proportion of linearity α. Also, note that this simulation satisfies the essential condition for causal inference, causal sufficiency. Specifically, Y is the direct product of X without a mediator (**Equation 9**). Since this means no latent confounder in the causal relationship from X to Y, it satisfies causal sufficiency. The other two essential causal assumptions, causal Markov and faithfulness are not relevant to the simulation since there is no other variable in the simulation that is conditionally dependent or independent of X and Y. Altogether, this simulation experiment is designed to evaluate the performance of causal inference methods in a straightforward setting. For each parameter combination (number of features and samples, complete- or partial-nonlinear), we ran various numbers of repetitions (50, 100, and 150), but report the results of 50 repetitions as different numbers of repetitions returned very similar results.

Competing Interest:

The authors declare that they have no competing interests.

Authors' contributions:

S.K. and H.J.P. conceived the project, designed the experiments, and wrote the manuscript. Z. F. implemented the method and interpreted the results. K.F.K., S.W.C, J.A.C. interpreted the results. P.V.B. discussed and designed the experiments.

Acknowledgments

We thank William Stafford Noble, Ph.D., Professor in Department of Genome Sciences and Department of Computer Science and Engineering, University of Washington for valuable discussion and their simulated and biological data. We thank Sonja Swanson, Ph.D. Visiting Associate Professor in the Department of Epidemiology, Gregory Cooper, MD, Ph.D., Professor in Department of Biomedical Informatics, University of Pittsburgh for valuable discussion on causal relationship learning. This research was supported in part by the University of Pittsburgh Center for Research Computing through the resources provided.

References

[1] S. Kim, H. J. Park, X. Cui, and D. Zhi, "Collective effects of long-range DNA methylations predict gene expressions and estimate phenotypes in cancer," *Sci. Rep.*, vol. 10, no. 1, p. 3920, 2020, doi: 10.1038/s41598-020-60845-2.

[2] S. Kim, Y. Bai, Z. Fan, B. Diergaarde, G. C. Tseng, and H. J. Park, "The microRNA target site landscape is a novel molecular feature associating alternative polyadenylation with immune evasion activity in breast cancer," *Brief. Bioinform.*, vol. 00, no. July, pp. 1–10, 2020, doi: 10.1093/bib/bbaa191.

[3] Z. Fan, S. Kim, Y. Bai, B. Diergaarde, and H. J. Park, "3′-UTR Shortening Contributes to

Subtype-Specific Cancer Growth by Breaking Stable ceRNA Crosstalk of Housekeeping Genes,” *Frontiers in Bioengineering and Biotechnology*, vol. 8. p. 334, 2020.

[4] A. J. Sedgewick, J. D. Ramsey, P. Spirtes, C. Glymour, and P. V. Benos, “Mixed Graphical Models for Causal Analysis of Multi-modal Variables,” *CoRR*, vol. abs/1704.0. 2017.

[5] P.-L. Loh and P. Bühlmann, “High-Dimensional Learning of Linear Causal Networks via Inverse Covariance Estimation,” *J. Mach. Learn. Res.*, vol. 15, no. 1, pp. 3065–3105, Jan. 2014.

[6] J. D. Lee and T. J. Hastie, “Structure learning of mixed graphical models,” *Journal of Machine Learning Research*, vol. 31. PMLR, pp. 388–396, 2013.

[7] R. Cui, P. Groot, and T. Heskes, *Copula PC Algorithm for Causal Discovery from Mixed Data*, vol. 9852. 2016.

[8] A. J. Sedgewick, I. Shi, R. M. Donovan, and P. V. Benos, “Learning mixed graphical models with separate sparsity parameters and stability-based model selection,” *BMC Bioinformatics*, vol. 17, no. 5, p. S175, 2016, doi: 10.1186/s12859-016-1039-0.

[9] R. F. Barber and E. J. Candes, “Controlling the false discovery rate via knockoffs,” *Ann. Stat.*, vol. 43, no. 5, pp. 2055–2085, 2015, doi: 10.1214/15-AOS1337.

[10] S. G. Bottcher and C. Dethlefsen, “Learning Bayesian Networks with Mixed Variables,” *Proceedings of the Eighth International Workshop on Artificial Intelligence and Statistics*, vol. R3. PMLR, pp. 13–20, 2011.

[11] V. Romero, R. Rumí, and A. Salmerón, “Learning hybrid Bayesian networks using mixtures of truncated exponentials,” *Int. J. Approx. Reason.*, vol. 42, no. 1, pp. 54–68, 2006, doi: https://doi.org/10.1016/j.ijar.2005.10.004.

[12] H. E. Kyburg, “Probabilistic Reasoning in Intelligent Systems: Networks of Plausible Inference by Judea Pearl,” *Journal of Philosophy*, vol. 88, no. 8. Morgan kaufmann, pp. 434–437, 1991, doi: 10.5840/jphil199188844.

[13] P. Spirtes, C. Glymour, and R. Scheines, *Causation, Prediction, and Search, 2nd Edition*, vol. 81. 2000.

[14] D. M. Chickering, “Optimal Structure Identification With Greedy Search.,” *J. Mach. Learn. Res.*, vol. 3, no. null, pp. 507–554, Jan. 2003, doi: 10.1162/153244303321897717.

[15] M. Koivisto and K. Sood, “Exact Bayesian Structure Discovery in Bayesian Networks,” *J. Mach. Learn. Res.*, vol. 5, pp. 549–573, Dec. 2004.

[16] T. Silander and P. Myllymäki, “A Simple Approach for Finding the Globally Optimal Bayesian Network Structure,” *ArXiv*, vol. abs/1206.6, Jun. 2006.

[17] T. Jaakkola, D. Sontag, A. Globerson, and M. M. B. T.-P. of the T. I. C. on A. I. and Statistics, “Learning Bayesian Network Structure using LP Relaxations,” vol. 9. PMLR, pp. 358–365.

[18] J. Cussens, “Bayesian Network Learning with Cutting Planes,” in *Proceedings of the Twenty-Seventh Conference on Uncertainty in Artificial Intelligence*, 2011, pp. 153–160.

[19] C. Yuan, B. Malone, and X. Wu, “Learning optimal Bayesian networks using A*search,” *IJCAI International Joint Conference on Artificial Intelligence*. pp. 2186–2191, Jul. 16, 2011, doi: 10.5591/978-1-57735-516-8/IJCAI11-364.

[20] T. Gao and D. Wei, “Parallel {B}ayesian Network Structure Learning,” in *Proceedings of the 35th International Conference on Machine Learning*, 2018, vol. 80, pp. 1685–1694, [Online]. Available: https://proceedings.mlr.press/v80/gao18b.html.

[21] G. Schwarz, “Estimating the Dimension of a Model,” *Ann. Stat.*, vol. 6, no. 2, pp. 461–464,

May 1978, [Online]. Available: http://www.jstor.org/stable/2958889.

[22] B. Andrews, J. Ramsey, and G. F. Cooper, “Learning High-dimensional Directed Acyclic Graphs with Mixed Data-types,” in *Proceedings of Machine Learning Research*, 2019, vol. 104, pp. 4–21, [Online]. Available: http://proceedings.mlr.press/v104/andrews19a.html.

[23] X. Zhang *et al.*, “Inferring gene regulatory networks from gene expression data by path consistency algorithm based on conditional mutual information,” *Bioinformatics*, vol. 28, no. 1, pp. 98–104, Jan. 2012, doi: 10.1093/bioinformatics/btr626.

[24] M. H. Maathuis, D. Colombo, M. Kalisch, and P. Bühlmann, “Predicting causal effects in large-scale systems from observational data,” *Nat. Methods*, vol. 7, no. 4, pp. 247–248, 2010, doi: 10.1038/nmeth0410-247.

[25] T. D. Le *et al.*, “Inferring microRNA–mRNA causal regulatory relationships from expression data,” *Bioinformatics*, vol. 29, no. 6, pp. 765–771, Mar. 2013, doi: 10.1093/bioinformatics/btt048.

[26] J. Zhang *et al.*, “Inferring condition-specific miRNA activity from matched miRNA and mRNA expression data,” *Bioinformatics*, vol. 30, no. 21, pp. 3070–3077, Nov. 2014, doi: 10.1093/bioinformatics/btu489.

[27] J. Zhang *et al.*, “Identifying direct miRNA–mRNA causal regulatory relationships in heterogeneous data,” *J. Biomed. Inform.*, vol. 52, pp. 438–447, 2014, doi: https://doi.org/10.1016/j.jbi.2014.08.005.

[28] C. Silverstein, S. Brin, R. Motwani, and J. Ullman, “Scalable Techniques for Mining Causal Structures,” *Data Min. Knowl. Discov.*, vol. 4, no. 2, pp. 163–192, 2000, doi: 10.1023/A:1009891813863.

[29] A. J. Sedgewick *et al.*, “Mixed graphical models for integrative causal analysis with application to chronic lung disease diagnosis and prognosis.,” *Bioinformatics*, vol. 35, no.

7, pp. 1204–1212, Apr. 2019, doi: 10.1093/bioinformatics/bty769.

[30] J. P. Higgins, “Nonlinear systems in medicine.,” *Yale J. Biol. Med.*, vol. 75, pp. 247–260, 2002.

[31] C. Trefois, P. M. A. Antony, J. Goncalves, A. Skupin, and R. Balling, “Critical transitions in chronic disease: transferring concepts from ecology to systems medicine,” *Curr. Opin. Biotechnol.*, vol. 34, pp. 48–55, 2015, doi: https://doi.org/10.1016/j.copbio.2014.11.020.

[32] N. Naik *et al.*, “Deep learning-enabled breast cancer hormonal receptor status determination from base-level H&E stains,” *Nat. Commun.*, vol. 11, no. 1, p. 5727, 2020, doi: 10.1038/s41467-020-19334-3.

[33] G. Lebedeva, A. Yamaguchi, S. P. Langdon, K. Macleod, and D. J. Harrison, “A model of estrogen-related gene expression reveals non-linear effects in transcriptional response to tamoxifen,” *BMC Syst. Biol.*, vol. 6, no. 1, p. 138, 2012, doi: 10.1186/1752-0509-6-138.

[34] M. Perera and C. Tsokos, “A Statistical Model with Non-Linear Effects and Non-Proportional Hazards for Breast Cancer Survival Analysis,” *Adv. Breast Cancer Res.*, vol. 07, pp. 65–89, Jan. 2018, doi: 10.4236/abcr.2018.71005.

[35] E. Candès, Y. Fan, L. Janson, and J. Lv, “Panning for gold: ‘model-X’ knockoffs for high dimensional controlled variable selection,” *J. R. Stat. Soc. Ser. B (Statistical Methodol.*, vol. 80, no. 3, pp. 551–577, Jun. 2018, doi: https://doi.org/10.1111/rssb.12265.

[36] Y. Y. Lu, Y. Fan, J. Lv, and W. S. Noble, “Deeppink: Reproducible feature selection in deep neural networks,” *Advances in Neural Information Processing Systems*, vol. 2018-Decem. pp. 8676–8686, Sep. 04, 2018.

[37] Y. Qin *et al.*, “Four computable 24-hour pediatric sepsis phenotypes have different inflammation profiles and heterogeneous outcome with anti-inflammatory therapies,” *Crit. Care*, 2022.

[38] C. B. Crayne, S. Albeituni, K. E. Nichols, and R. Q. Cron, “The Immunology of Macrophage Activation Syndrome.,” *Front. Immunol.*, vol. 10, p. 119, 2019, doi: 10.3389/fimmu.2019.00119.

[39] I. Ushach and A. Zlotnik, “Biological role of granulocyte macrophage colony-stimulating factor (GM-CSF) and macrophage colony-stimulating factor (M-CSF) on cells of the myeloid lineage,” *J. Leukoc. Biol.*, vol. 100, no. 3, pp. 481–489, Sep. 2016, doi: 10.1189/jlb.3RU0316-144R.

[40] S. L. Deshmane, S. Kremlev, S. Amini, and B. E. Sawaya, “Monocyte Chemoattractant Protein-1 (MCP-1): An Overview,” *J. Interf. Cytokine Res.*, vol. 29, no. 6, pp. 313–326, May 2009, doi: 10.1089/jir.2008.0027.

[41] L. Zhu, Q. Zhao, T. Yang, W. Ding, and Y. Zhao, “Cellular metabolism and macrophage functional polarization.,” *Int. Rev. Immunol.*, vol. 34, no. 1, pp. 82–100, Jan. 2015, doi: 10.3109/08830185.2014.969421.

[42] A. Dige *et al.*, “Soluble CD163, a Specific Macrophage Activation Marker, is Decreased by Anti-TNF-α Antibody Treatment in Active Inflammatory Bowel Disease,” *Scand. J. Immunol.*, vol. 80, no. 6, pp. 417–423, Dec. 2014, doi: https://doi.org/10.1111/sji.12222.

[43] N. Rittig, M. Svart, N. Jessen, N. Møller, H. J. Møller, and H. Grønbæk, “Macrophage activation marker sCD163 correlates with accelerated lipolysis following LPS exposure: a human-randomised clinical trial,” *Endocr. Connect.*, vol. 7, no. 1, pp. 107–114, 2018, doi: 10.1530/EC-17-0296.

[44] A. V Finn *et al.*, “Hemoglobin directs macrophage differentiation and prevents foam cell formation in human atherosclerotic plaques,” *J. Am. Coll. Cardiol.*, vol. 59, no. 2, pp. 166–177, Jan. 2012, doi: 10.1016/j.jacc.2011.10.852.

[45] S. Fleming *et al.*, “Normal ranges of heart rate and respiratory rate in children from birth to

18 years of age: a systematic review of observational studies," *Lancet*, vol. 377, no. 9770, pp. 1011–1018, 2011, doi: https://doi.org/10.1016/S0140-6736(10)62226-X.

[46] D. R. Jury, "Serum creatinine concentration in children: normal values for sex and age," *N. Z. Med. J.*, vol. 90, no. 649, pp. 453–456, 1979, [Online]. Available: http://europepmc.org/abstract/MED/294520.

[47] W. T. Shearer *et al.*, "Lymphocyte subsets in healthy children from birth through 18 years of age: The pediatric AIDS clinical trials group P1009 study," *J. Allergy Clin. Immunol.*, vol. 112, no. 5, pp. 973–980, 2003, doi: https://doi.org/10.1016/j.jaci.2003.07.003.

[48] M. W. Merx and C. Weber, "Sepsis and the Heart," *Circulation*, vol. 116, no. 7, pp. 793–802, Aug. 2007, doi: 10.1161/CIRCULATIONAHA.106.678359.

[49] L. Ma *et al.*, "Role of interleukin-6 to differentiate sepsis from non-infectious systemic inflammatory response syndrome," *Cytokine*, vol. 88, pp. 126–135, 2016, doi: https://doi.org/10.1016/j.cyto.2016.08.033.

[50] C. Mitaka, "Clinical laboratory differentiation of infectious versus non-infectious systemic inflammatory response syndrome," *Clin. Chim. Acta*, vol. 351, no. 1, pp. 17–29, 2005, doi: https://doi.org/10.1016/j.cccn.2004.08.018.

[51] K. Nakanishi, "Unique Action of Interleukin-18 on T Cells and Other Immune Cells," *Frontiers in Immunology*, vol. 9. p. 763, 2018.

[52] J. R. Schoenborn and C. B. Wilson, "Regulation of interferon-gamma during innate and adaptive immune responses.," *Adv. Immunol.*, vol. 96, pp. 41–101, 2007, doi: 10.1016/S0065-2776(07)96002-2.

[53] A. C. Stanley and P. Lacy, "Pathways for Cytokine Secretion," *Physiology*, vol. 25, no. 4, pp. 218–229, Aug. 2010, doi: 10.1152/physiol.00017.2010.

[54] W. J. Leonard and J.-X. Lin, “Cytokine receptor signaling pathways,” *J. Allergy Clin. Immunol.*, vol. 105, no. 5, pp. 877–888, 2000, doi: https://doi.org/10.1067/mai.2000.106899.

[55] W. Tate *et al.*, “Molecular Mechanisms of Neuroinflammation in ME/CFS and Long COVID to Sustain Disease and Promote Relapses ,” *Frontiers in Neurology* , vol. 13. 2022, [Online]. Available: https://www.frontiersin.org/article/10.3389/fneur.2022.877772.

[56] L. Zhao *et al.*, “Sepsis-Associated Encephalopathy: Insight into Injury and Pathogenesis,” *CNS & Neurological Disorders - Drug Targets*, vol. 20, no. 2. pp. 112–124, 2021, doi: http://dx.doi.org/10.2174/1871527319999201117122158.

[57] G. F. Weber, S. Schlautkötter, S. Kaiser-Moore, F. Altmayr, B. Holzmann, and H. Weighardt, “Inhibition of interleukin-22 attenuates bacterial load and organ failure during acute polymicrobial sepsis.,” *Infect. Immun.*, vol. 75, no. 4, pp. 1690–1697, Apr. 2007, doi: 10.1128/IAI.01564-06.

[58] S. Manicka, K. Johnson, M. Levin, and D. Murrugarra, “Biological regulatory networks are less nonlinear than expected by chance,” *bioRxiv*, p. 2021.12.22.473903, Jan. 2021, doi: 10.1101/2021.12.22.473903.

[59] T. Kapitaniak and S. Jafari, “Nonlinear effects in life sciences,” *Eur. Phys. J. Spec. Top.*, vol. 227, no. 7, pp. 693–696, 2018, doi: 10.1140/epjst/e2018-800104-6.

[60] R. Stoof and Á. Goñi-Moreno, “Modelling co-translational dimerization for programmable nonlinearity in synthetic biology,” *J. R. Soc. Interface*, vol. 17, no. 172, p. 20200561, Nov. 2020, doi: 10.1098/rsif.2020.0561.

[61] G. D. Wu *et al.*, “Linking Long-Term Dietary Patterns with Gut Microbial Enterotypes,” *Science (80-.).*, vol. 334, no. 6052, pp. 105–108, May 2011, [Online]. Available: http://www.jstor.org/stable/23059312.

[62] Y.-S. Kuang *et al.*, "Connections between the human gut microbiome and gestational diabetes mellitus.," *Gigascience*, vol. 6, no. 8, pp. 1–12, Aug. 2017, doi: 10.1093/gigascience/gix058.

[63] Q. Yang, "Gain weight by 'going diet?' Artificial sweeteners and the neurobiology of sugar cravings: Neuroscience 2010," *Yale J. Biol. Med.*, vol. 83, no. 2, pp. 101–108, Jun. 2010, [Online]. Available: https://pubmed.ncbi.nlm.nih.gov/20589192.

[64] Y. Yun *et al.*, "Comparative analysis of gut microbiota associated with body mass index in a large Korean cohort.," *BMC Microbiol.*, vol. 17, no. 1, p. 151, Jul. 2017, doi: 10.1186/s12866-017-1052-0.

[65] D. C. Koboldt *et al.*, "Comprehensive molecular portraits of human breast tumours," *Nature*, vol. 490, no. 7418, pp. 61–70, 2012, doi: 10.1038/nature11412.

[66] T. Pascual *et al.*, "A Pathology-Based Combined Model to Identify PAM50 Non-luminal Intrinsic Disease in Hormone Receptor-Positive HER2-Negative Breast Cancer ," *Frontiers in Oncology* , vol. 9. 2019, [Online]. Available: https://www.frontiersin.org/article/10.3389/fonc.2019.00303.

[67] T. O. Nielsen *et al.*, "A Comparison of PAM50 Intrinsic Subtyping with Immunohistochemistry and Clinical Prognostic Factors in Tamoxifen-Treated Estrogen Receptor–Positive Breast Cancer," *Clin. Cancer Res.*, vol. 16, no. 21, pp. 5222–5232, Oct. 2010, doi: 10.1158/1078-0432.CCR-10-1282.

[68] M. Rossing *et al.*, "Clinical implications of intrinsic molecular subtypes of breast cancer for sentinel node status," *Sci. Rep.*, vol. 11, no. 1, p. 2259, 2021, doi: 10.1038/s41598-021-81538-4.

[69] E. A. Mittendorf, J. M. S. Bartlett, D. L. Lichtensztajn, and S. Chandarlapaty, "Incorporating Biology Into Breast Cancer Staging: American Joint Committee on Cancer,

Eighth Edition, Revisions and Beyond," *Am. Soc. Clin. Oncol. Educ. B.*, no. 38, pp. 38–46, May 2018, doi: 10.1200/EDBK_200981.

[70] A. A. Onitilo, J. M. Engel, R. T. Greenlee, and B. N. Mukesh, "Breast cancer subtypes based on ER/PR and Her2 expression: comparison of clinicopathologic features and survival," *Clin. Med. Res.*, vol. 7, no. 1–2, pp. 4–13, Jun. 2009, doi: 10.3121/cmr.2009.825.

[71] X. Dai, A. Chen, and Z. Bai, "Integrative investigation on breast cancer in ER, PR and HER2-defined subgroups using mRNA and miRNA expression profiling," *Sci. Rep.*, vol. 4, no. 1, p. 6566, 2014, doi: 10.1038/srep06566.

[72] A. J. Brooks, J. W. Wooh, K. A. Tunny, and M. J. Waters, "Growth hormone receptor; mechanism of action," *Int. J. Biochem. Cell Biol.*, vol. 40, no. 10, pp. 1984–1989, 2008, doi: https://doi.org/10.1016/j.biocel.2007.07.008.

[73] K. P. Harden and K. L. Klump, "Introduction to the Special Issue on Gene-Hormone Interplay," *Behav. Genet.*, vol. 45, no. 3, pp. 263–267, 2015, doi: 10.1007/s10519-015-9717-7.

[74] M. W. Coolen *et al.*, "Consolidation of the cancer genome into domains of repressive chromatin by long-range epigenetic silencing (LRES) reduces transcriptional plasticity.," *Nat. Cell Biol.*, vol. 12, no. 3, pp. 235–246, Mar. 2010, doi: 10.1038/ncb2023.

[75] S. Ashida *et al.*, "Integrated analysis reveals critical genomic regions in prostate tumor microenvironment associated with clinicopathologic phenotypes.," *Clin. cancer Res. an Off. J. Am. Assoc. Cancer Res.*, vol. 18, no. 6, pp. 1578–1587, Mar. 2012, doi: 10.1158/1078-0432.CCR-11-2535.

[76] P. Flaherty, P. Wiratchotisatian, J. A. Lee, Z. Tang, and A. C. Trapp, "MAP Clustering under the Gaussian Mixture Model via Mixed Integer Nonlinear Optimization." arXiv, 2019,

doi: 10.48550/ARXIV.1911.04285.

[77] H. J. Park *et al.*, “3′ UTR shortening represses tumor-suppressor genes in trans by disrupting ceRNA crosstalk,” *Nat. Genet.*, vol. 50, pp. 783–789, 2018, doi: 10.1038/s41588-018-0118-8.

[78] S. Kim *et al.*, “Expression Quantitative Trait Methylation Analysis Reveals Methylomic Associations With Gene Expression in Childhood Asthma,” *Chest*, vol. 158, no. 5, pp. 1841–1856, Nov. 2020, doi: 10.1016/j.chest.2020.05.601.

[79] R. Karki *et al.*, “Synergism of TNF-α and IFN-γ Triggers Inflammatory Cell Death, Tissue Damage, and Mortality in SARS-CoV-2 Infection and Cytokine Shock Syndromes,” *Cell*, vol. 184, no. 1, pp. 149-168.e17, 2021, doi: https://doi.org/10.1016/j.cell.2020.11.025.

[80] R. N. Gomes *et al.*, “Bacterial clearance in septic mice is modulated by MCP-1/CCL2 and nitric oxide.,” *Shock*, vol. 39, no. 1, pp. 63–69, Jan. 2013, doi: 10.1097/SHK.0b013e31827802b5.

[81] M. Kormaksson, L. J. Kelly, X. Zhu, S. Haemmerle, L. Pricop, and D. Ohlssen, “Sequential knockoffs for continuous and categorical predictors: With application to a large psoriatic arthritis clinical trial pool,” *Stat. Med.*, vol. 40, no. 14, pp. 3313–3328, 2021.

[82] J. Chen and H. Li, “Variable selection for sparse Dirichlet-multinomial regression with an application to microbiome data analysis,” *Ann. Appl. Stat.*, vol. 7, no. 1, pp. 418–442, Mar. 2013, doi: 10.1214/12-AOAS592.

[83] W. Lin, P. Shi, R. Feng, and H. Li, “Variable selection in regression with compositional covariates,” *Biometrika*, vol. 101, no. 4, pp. 785–797, Dec. 2014, doi: 10.1093/biomet/asu031.

[84] Z. Zheng, J. Lv, and W. Lin, “Nonsparse learning with latent variables.” arXiv, 2017, doi: 10.48550/ARXIV.1710.02704.

[85] D. J. Slamon *et al.*, “Studies of the HER-2/neu Proto-Oncogene in Human Breast and Ovarian Cancer,” *Science (80-.).*, vol. 244, no. 4905, pp. 707–712, May 1989, [Online]. Available: http://www.jstor.org/stable/1703358.

[86] J. A. Carcillo *et al.*, “A Multicenter Network Assessment of Three Inflammation Phenotypes in Pediatric Sepsis-Induced Multiple Organ Failure.,” *Pediatr. Crit. care Med. a J. Soc. Crit. Care Med. World Fed. Pediatr. Intensive Crit. Care Soc.*, vol. 20, no. 12, pp. 1137–1146, Dec. 2019, doi: 10.1097/PCC.0000000000002105.

[87] B. Goldstein, B. Giroir, and A. Randolph, “International pediatric sepsis consensus conference: definitions for sepsis and organ dysfunction in pediatrics.,” *Pediatr. Crit. care Med. a J. Soc. Crit. Care Med. World Fed. Pediatr. Intensive Crit. Care Soc.*, vol. 6, no. 1, pp. 2–8, Jan. 2005, doi: 10.1097/01.PCC.0000149131.72248.E6.

[88] A. Villeneuve, J.-S. Joyal, F. Proulx, T. Ducruet, N. Poitras, and J. Lacroix, “Multiple organ dysfunction syndrome in critically ill children: clinical value of two lists of diagnostic criteria.,” *Ann. Intensive Care*, vol. 6, no. 1, p. 40, Dec. 2016, doi: 10.1186/s13613-016-0144-6.

[89] A. Yazdani, A. Yazdani, A. Samiei, and E. Boerwinkle, “Identification, analysis, and interpretation of a human serum metabolomics causal network in an observational study.,” *J. Biomed. Inform.*, vol. 63, pp. 337–343, Oct. 2016, doi: 10.1016/j.jbi.2016.08.017.

[90] A. F. Agarap, “Deep Learning using Rectified Linear Units (ReLU).” arXiv, 2018, doi: 10.48550/ARXIV.1803.08375.

[91] X. Glorot and Y. Bengio, “Understanding the difficulty of training deep feedforward neural networks,” in *Proceedings of the Thirteenth International Conference on Artificial Intelligence and Statistics*, 2010, vol. 9, pp. 249–256, [Online]. Available: https://proceedings.mlr.press/v9/glorot10a.html.

[92] C. Glymour, K. Zhang, and P. Spirtes, “Review of Causal Discovery Methods Based on Graphical Models ,” *Frontiers in Genetics* , vol. 10. p. 524, 2019.

[93] E. Candes, Y. Fan, L. Janson, and J. Lv, “Panning for Gold: Model-free Knockoffs for High-dimensional Controlled Variable Selection,” *J. R. Stat. Soc. Ser. B (Statistical Methodol.*, vol. 80, Oct. 2016, doi: 10.1111/rssb.12265.

[94] W. Hardle and T. M. Stoker, “Investigating Smooth Multiple Regression by the Method of Average Derivatives,” *J. Am. Stat. Assoc.*, vol. 84, no. 408, pp. 986–995, Mar. 1989, doi: 10.2307/2290074.

[95] H. Ichimura, “Semiparametric least squares (SLS) and weighted SLS estimation of single-index models,” *J. Econom.*, vol. 58, no. 1, pp. 71–120, 1993, doi: https://doi.org/10.1016/0304-4076(93)90114-K.

[96] R. J. Carroll, J. Fan, I. Gijbels, and M. P. Wand, “Generalized Partially Linear Single-Index Models,” *J. Am. Stat. Assoc.*, vol. 92, no. 438, pp. 477–489, Mar. 1997, doi: 10.2307/2965697.

[97] L. Wang and L. Yang, “SPLINE ESTIMATION OF SINGLE-INDEX MODELS,” *Stat. Sin.*, vol. 19, no. 2, pp. 765–783, Mar. 2009, [Online]. Available: http://www.jstor.org/stable/24308855.

[98] Y. Yu, J. Chen, T. Gao, and M. Yu, “DAG-GNN: DAG structure learning with graph neural networks,” *36th Int. Conf. Mach. Learn. ICML 2019*, vol. 2019-June, pp. 12395–12406, 2019.

[99] H. Blankson, J. A. Stakkestad, H. Fagertun, E. Thom, J. Wadstein, and O. Gudmundsen, “Conjugated linoleic acid reduces body fat mass in overweight and obese humans.,” *J. Nutr.*, vol. 130, no. 12, pp. 2943–2948, Dec. 2000, doi: 10.1093/jn/130.12.2943.

[100] C.-M. Chiu *et al.*, “Systematic analysis of the association between gut flora and obesity

through high-throughput sequencing and bioinformatics approaches.," *Biomed Res. Int.*, vol. 2014, p. 906168, 2014, doi: 10.1155/2014/906168.

[101] M. Vanhala *et al.*, "Serum omega-6 polyunsaturated fatty acids and the metabolic syndrome: a longitudinal population-based cohort study.," *Am. J. Epidemiol.*, vol. 176, no. 3, pp. 253–260, Aug. 2012, doi: 10.1093/aje/kwr504.

[102] L. Pimpin, S. Jebb, L. Johnson, J. Wardle, and G. L. Ambrosini, "Dietary protein intake is associated with body mass index and weight up to 5 y of age in a prospective cohort of twins.," *Am. J. Clin. Nutr.*, vol. 103, no. 2, pp. 389–397, Feb. 2016, doi: 10.3945/ajcn.115.118612.

[103] S. Rabot *et al.*, "High fat diet drives obesity regardless the composition of gut microbiota in mice," *Sci. Rep.*, vol. 6, no. 1, p. 32484, 2016, doi: 10.1038/srep32484.

[104] D. N. Reeds, B. S. Mohammed, S. Klein, C. B. Boswell, and V. L. Young, "Metabolic and structural effects of phosphatidylcholine and deoxycholate injections on subcutaneous fat: a randomized, controlled trial.," *Aesthetic Surg. J.*, vol. 33, no. 3, pp. 400–408, Mar. 2013, doi: 10.1177/1090820X13478630.

[105] Y. J. Yang, Y. J. Kim, Y. K. Yang, J. Y. Kim, and O. Kwon, "Dietary flavan-3-ols intake and metabolic syndrome risk in Korean adults.," *Nutr. Res. Pract.*, vol. 6, no. 1, pp. 68–77, Feb. 2012, doi: 10.4162/nrp.2012.6.1.68.

Figures and Tables

Figure 1. Overview of DAG-deepVASE. (A) An input data matrix consisting of *M* variables (V_1, V_2, ..., V_M), either continuous or ordinal categorical, collected from *N* samples. (B) Left: An example of the identified linear associations using a statistical graphical model (MGM). Right: identifying nonlinear associations using a deep neural network (deep-learning) model. After the first run sets V_1 as response and identifies its association with other variables, DAG-deepVASE will run this model with each of the other variables (V_2, V_3, ..., V_M) as response and with all the other variables as input. (C) Left: estimating the effect size of linear associations in the statistical graphical model. Right: estimating the effect size of nonlinear associations in reference to knockoff filter implemented in the deep-learning model. (D) Learning the causalities by running the degenerate Gaussian (DG) separately on the identified associations, either linear or nonlinear.

Figure 2. Performance assessment of causal inference methods on the simulated data AUC estimated for DAG-deepVASE and causalMGM on **(A)** 20 true and false associations and **(B)** 40 true and false associations, both under complete-nonlinear scenarios. **(C)** Average number, and standard error (error bar), of true associations in the complete-nonlinear scenario identified by DAG-deepVASE (red), causalMGM (gray), or linear-DG (yellow) over 50 runs in various simulation scenarios, varying the number of features and sample sizes. Average number, and standard error (error bar), of **(D)** true causalities and **(E)** false causalities identified by DAG-deepVASE (red), causalMGM (gray), or linear-DG (yellow) over 50 runs. DAG-deepVASE and linear-DG did not identify any false causalities.

Figure 3. Linear and nonlinear associations in pediatric sepsis data (A) Number of linear (blue) and nonlinear (red) associations involving each of the 45 variables. **(B)** A subnetwork of linear (blue) or nonlinear (red) variable associations involving SIRS (associated only non-linearly)

and sCD183 (associated only linearly) and with normalized effect size. Gray nodes connect between IFN-γ and TNFα. For full names of the variables, readers are referred to Methods.

Figure 4. Performance assessment of four causal inference methods on various degrees of nonlinear associations in BMI/bacteria/gut microbiome data (A) Number of associations the methods (causalMGM, linear-DG, and DAG-deepVASE) identified between the BMI status and 8 nutrient intake (blue) and 8 bacteria genera in the gut (red) that are validated associated with the BMI status. **(B)** The relationship between BMI and Firmicutes-Allisonella identified with confidence interval (gray intervals). Red line represents the estimated linear regression and p-value for linear fit is calculated from a permutation test with R^2 (Methods). **(C)** The relationship between BMI and Choline, Phosphatidylcholine w/o suppl identified with confidence interval (gray intervals). Blue line connects the middle point of the BMI values 1 to 5. **(D)** Number of true positive (dark blue) and false positive (red) causalities identified by causalMGM, linear-DG, and DAG-deepVASE. DAG-deepVASE and linear-DG did not identify any false causalities.

Figure 5. DAG-deepVASE on TCGA breast cancer data. (A) Number of validated associations from molecular (blue) and clinical (orange) variables to PAM50 identified by causalMGM, linear-DG, and DAG-deepVASE. **(B)** Number of causalities identified by causalMGM, linear-DG, and DAG-deepVASE. **(C)** Number of linear and nonlinear causalities DAG-deepVASE learned between two of the 10 genes, between a gene and a clinical variable, or between two of the 6 clinical variables. **(D)** Causalities inferred by DAG-deepVASE over 10 molecular variables, 5 clinical variables, and the PAM50 status as linear (purple) and nonlinear (red) by DAG-deepVASE. ‘person_neoplasm_cancer_status’ refers to the state or condition of an individual's neoplasm. ‘PR_status’, ‘ER_status’, and ‘HER2_status’ refer to the status of progesterone receptor, estrogen receptor, and human epidermal growth factor 2 receptor in the tumor sample.

Table 1. Variables in the pediatric sepsis data.

Variable	Description of variable
**Demographic**	
Age	
**PRISM[a]**	
Low SBP	Lowest Systolic Blood Pressure
High Heart Rate	Highest Heart Rate
Low Temp	Lowest Temperature
High Temp	Highest Temperature
GCS	The lowest GCS score
Lower Platelet	Lowest Platelets
**Labs**	
Higher Creatinine	Highest value from PRISM High Creatinine and High Creatinine
Low Lymphocyte	Absolute lymphocyte count
Low Hemoglobin	Hemoglobin
Low Platelet	Platelet count
ex vivo TNF-α	Blood endotoxin-stimulated TNF-α
SFASLigand	sFas Ligand

sCD163	Soluble CD163
ADAMTS13	A disintegrin and metalloproteinase with a thrombospondin type 1 motif, member 13
**Organ failure**	
SIRS	Systemic Inflammatory Response Syndrome criteria
**Cytokine**	
CRP	C-reactive protein
IFN-β	Interferon-β
IL-22	Interleukin-22
IL-18	Interleukin-18
IL-18BP	Interleukin-18-binding protein
MIG-CXCL9	Chemokine (C-X-C motif) ligand 9 (CXCL9) or monokine induced by interferon gamma (MIG)
IL-1β	Interleukin 1β
IL-4	Interleukin-4
IL-6	Interleukin-6
IL-8	Interleukin-8
IL-10	Interleukin-10
IL-13	Interleukin-13

IL-17A	Interleukin-17A
IFN-γ	Interferon-γ
IP-10/CXCL10	C–X–C motif chemokine 10 (CXCL10) or interferon γ-induced protein 10 kDa (IP-10)
MCP-1/CCL2	Chemokine (C-C motif) ligand 2 (CCL2) or monocyte chemoattractant protein 1 (MCP1)
MIP-1α	Macrophage inflammatory protein-1 alpha
MIP-1β	Macrophage inflammatory protein-1β
TNF-α	Tumor necrosis factor α
MCP-3	Monocyte chemotactic protein-3
IFN.α2	Interferon α-2
IL-1α	Interleukin 1α
IL-2Ra	Interleukin-2 receptor antagonists
IL-3	Interleukin-3
IL-16	Interleukin-16
M-CSF	Macrophage colony-stimulating factor
SCF	Stem cell factor
Trail	Trial
Ferritin	Ferritin

Table 2. Parameter settings for the deep-learning component of DAG-deepVASE.

	Parameters	Value
DNN	Activation function	Rectified linear unit (ReLU)
	Initial weight values	Glorot normal intializer
	Regularization	$L1\text{−}regularization$
	Optimization	Adam optimization
	Loss function	Mean of squares of errors (MSE)
FDR	FDR control rate	0.05

Table 3. 16 nonlinear associations (8 nutrient intakes and 8 bacteria genera) that were validated in literature.

	Nutrient intake		Bacteria genera		
	Micronutrient	Reference	Phylum	Genus	Reference
1	Linoleic	[99]	Proteobacteria	Sutterella	[100]
2	Omega 6	[101]	Firmicutes	Allisonella	[83]
3	Dairy Protein	[102]	Firmicutes	Holdemania	[103]
4	Aspartic Acid, Aspartame	[63]	Firmicutes	Mitsuokella	[64]
5	Phenylalanine, Aspartame	[63]	Firmicutes	Clostridium	[83]
6	Choline, Phosphatidylcholine	[104]	Firmicutes	Megamonas	[62]
7	Theaflavin 3-gallate, flavan-3-ol(2)	[105]	Firmicutes	Megasphaera	[64]
8	Choline, Phosphatidylcholine w/o suppl.	[104]	Firmicutes	Acidaminococcus	[83]

Figure
Click here to access/download;Figure;Fig1.pdf

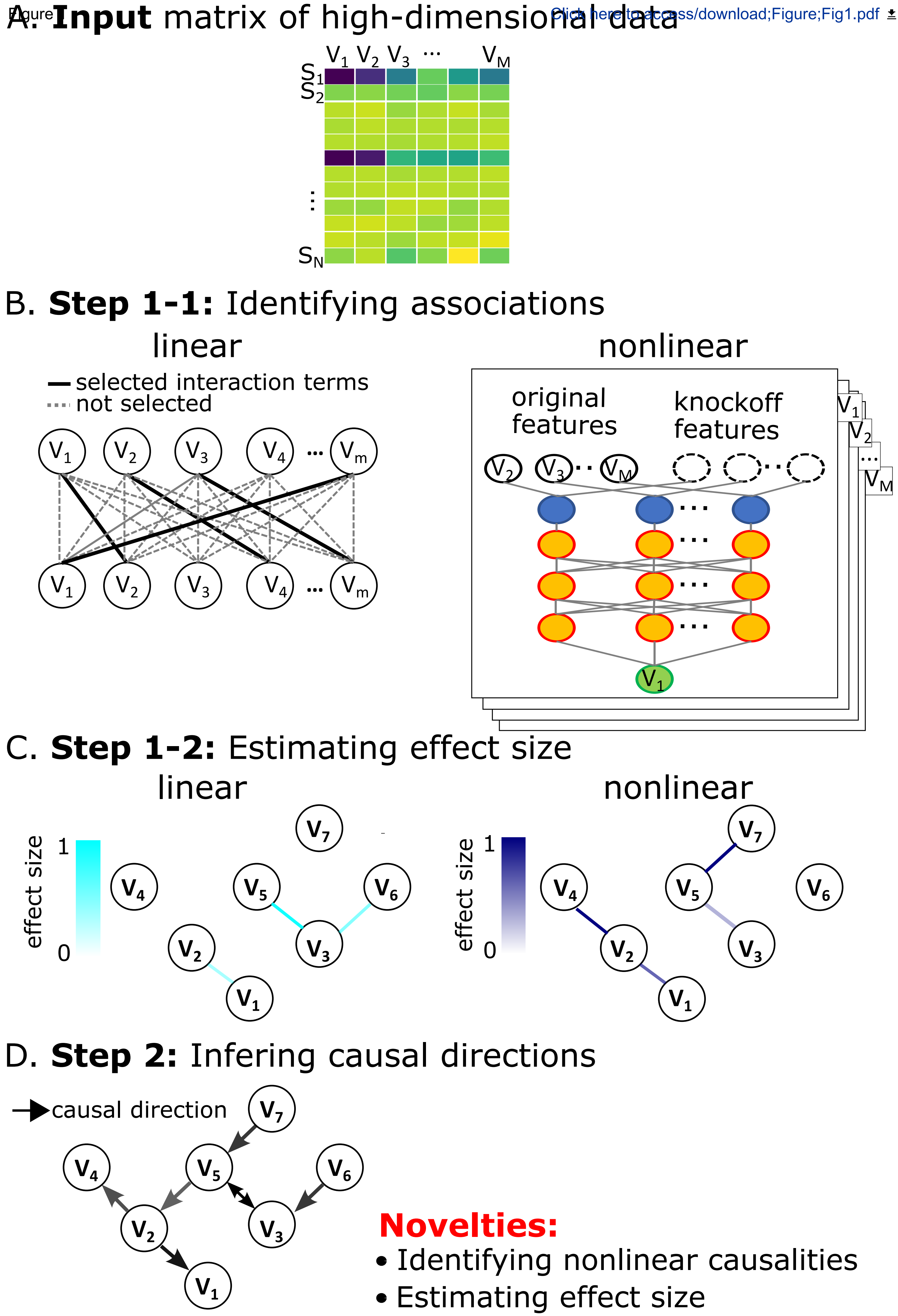

Figure 2
Click here to access/download;Figure;Fig2.pdf

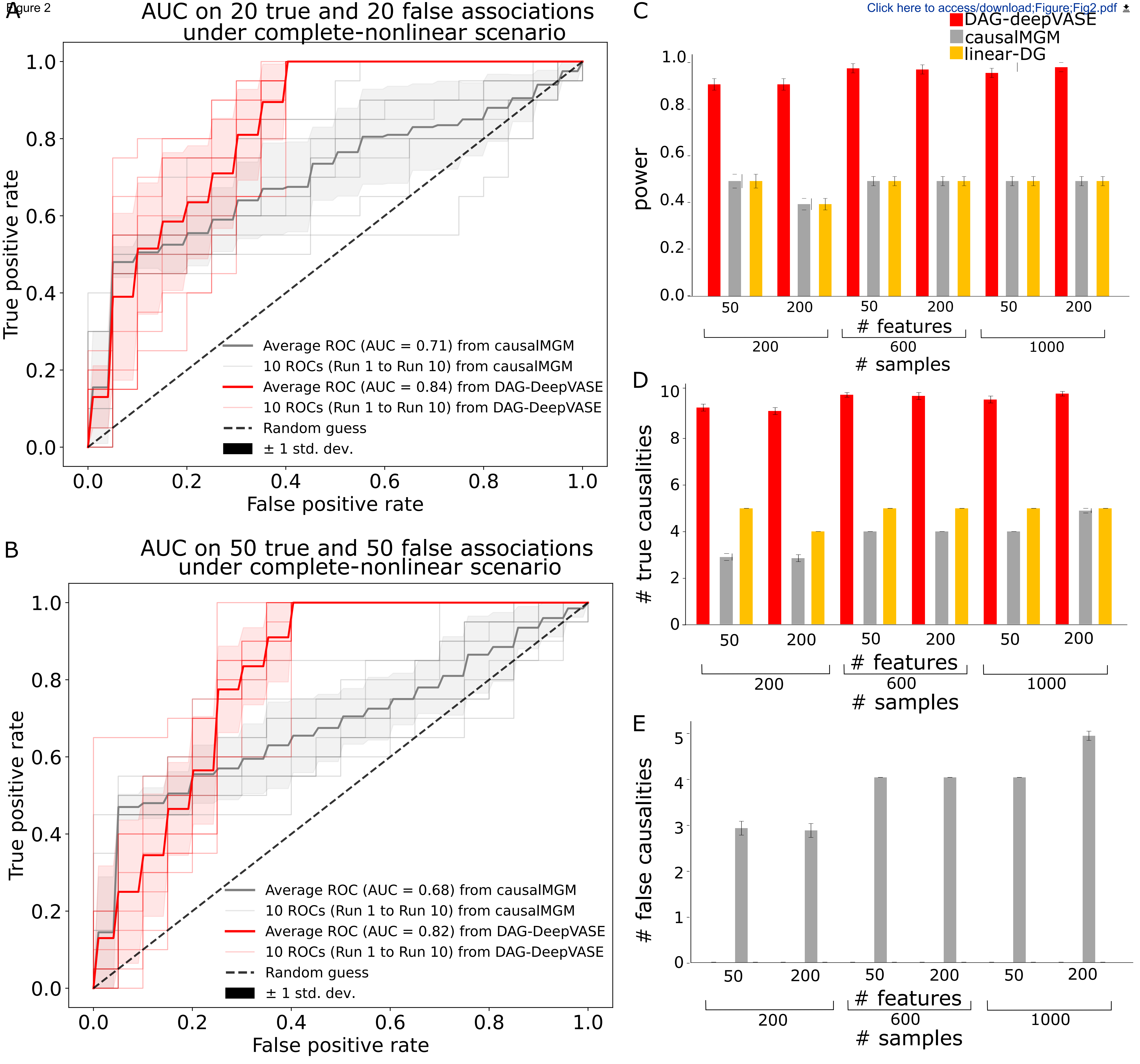

Figure 3

Click here to access/download;Figure;Fig3.pdf

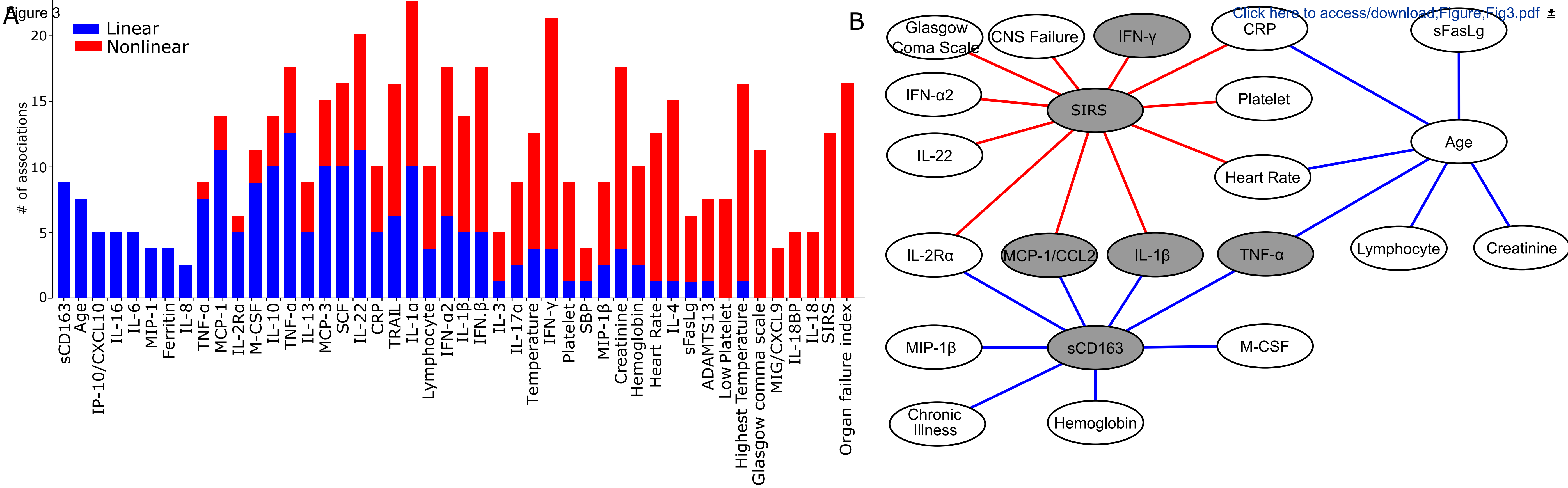

Figure 4

Click here to access/download;Figure;Fig4.pdf

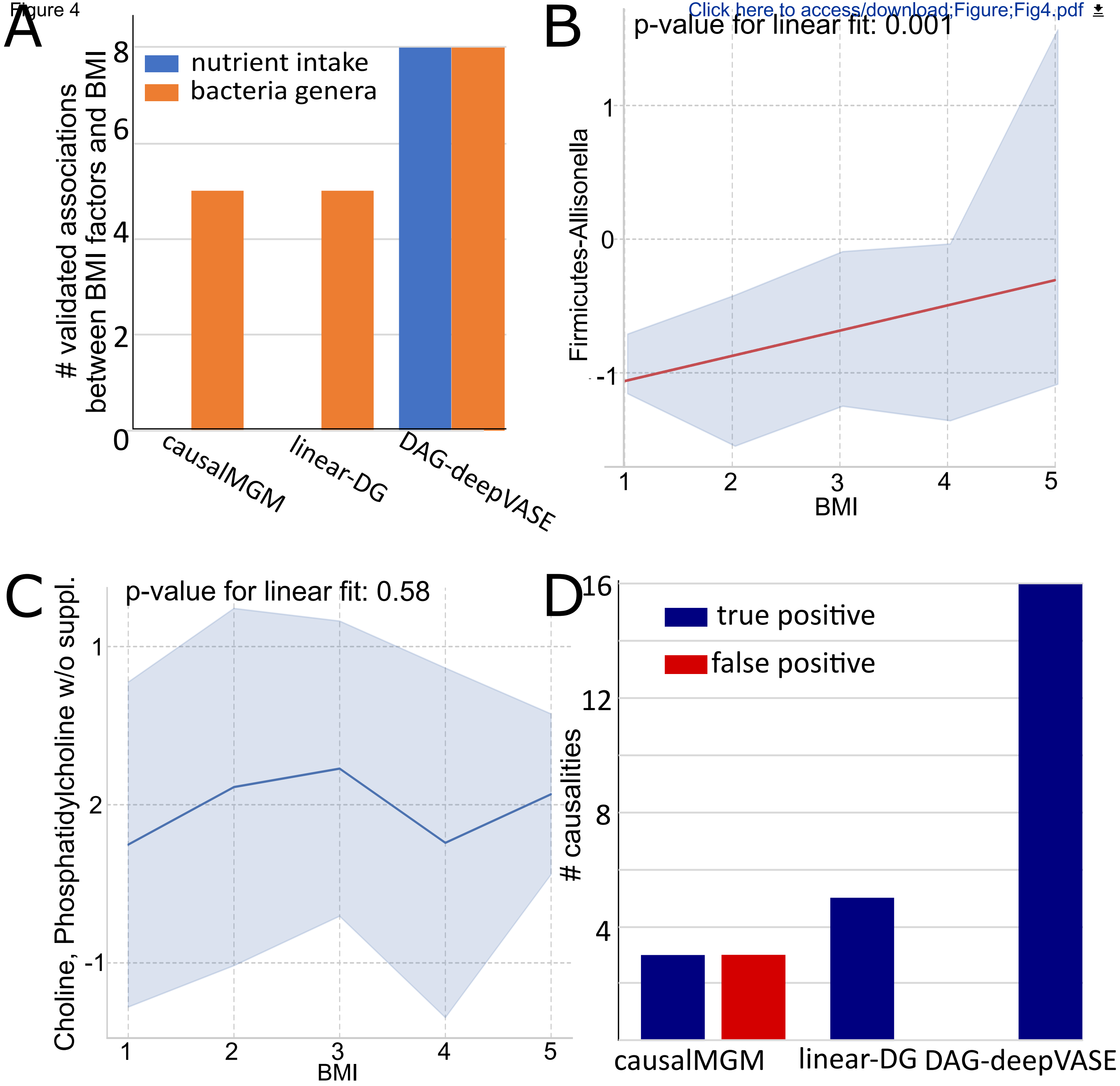

Figure 5

Click here to access/download;Figure;Fig5.pdf

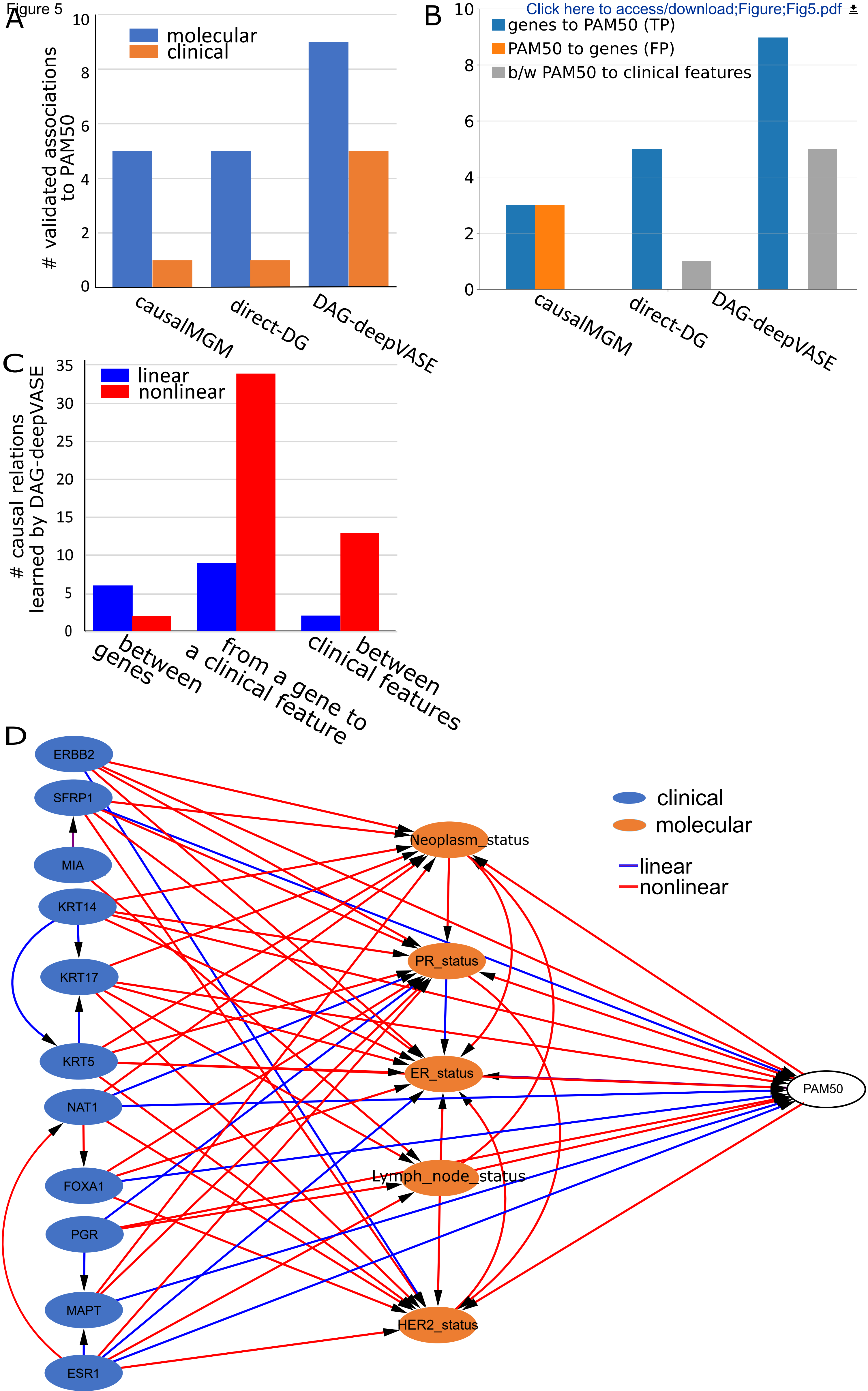

Supplementary Figures

Click here to access/download
Supplementary Material
Supplemental Figures_Supplementary Material.docx

Supplementary Tables

Click here to access/download
Supplementary Material
Supplementary Data.xls

cover letter Click here to access/download;Personal Cover;cover_letter.pdf

University of Pittsburgh

Graduate School of Public Health

Department of Human Genetics

Hyun Jung Park, Ph.D.
Assistant Professor
Department of Human Genetics
Department of Biostatistics
School of Public Health
Department of Biomedical Informatics
University of Pittsburgh
E-mail: hyp15@pitt.edu
http://www.parklab.pitt.edu/

September 4th, 2022

Dear Editors in GigaScience,

I would like to submit our manuscript, "**Deep neural networks with knockoff features identify nonlinear causal relations and estimate effect sizes in complex biological systems**" for publication. Causal inference is at the core of making an actionable understanding of complex systems, including complex diseases. In complex systems typically of multiple regulatory layers, features are expected to interact through multiple layers, some unobserved or latent, rendering variable interactions highly nonlinear. However, existing causal inference methods are not designed to identify nonlinear causal relationships. In this manuscript, we developed the first computational method that identifies and incorporates both linear and nonlinear causalities, named causal directed acyclic graph using deep-learning variable-selection (DAG-deepVASE). Using simulated and biological data sets of complex diseases, we demonstrated that DAG-deepVASE outperforms existing methods in identifying expected or validated causal relationships. Further, we illustrated how causalDeepVASE enables researchers to build insights into complex systems, which is not possible in existing methods.

The Journal of GigaScience would be an outstanding journal for this paper due to its intention of featuring machine learning research works that advance the understanding of complex biological systems.

I confirm that

- none of the material has been published or is under consideration for publication elsewhere
- no potential conflict of interest
- all authors have approved the manuscript for submission

We hope that you will decide favorably to send it out for peer review.

Best Regards,

Hyun Jung Park, Ph.D.
