# Supplementary material for: Deep neural networks with knockoff features identify nonlinear causal relations and estimate effect sizes in complex biological systems
Source: Gigascience. 2023 Jul 3;12:giad044. doi: 10.1093/gigascience/giad044 (PMC10316696; doi:10.1093/gigascience/giad044)
Supplement: giad044_Supplemental_Files [file giad044_supplemental_files.zip › Supplemental Figures_Supplementary Material.docx]

Supplemental Figure 1_ Supplementary Material.

**
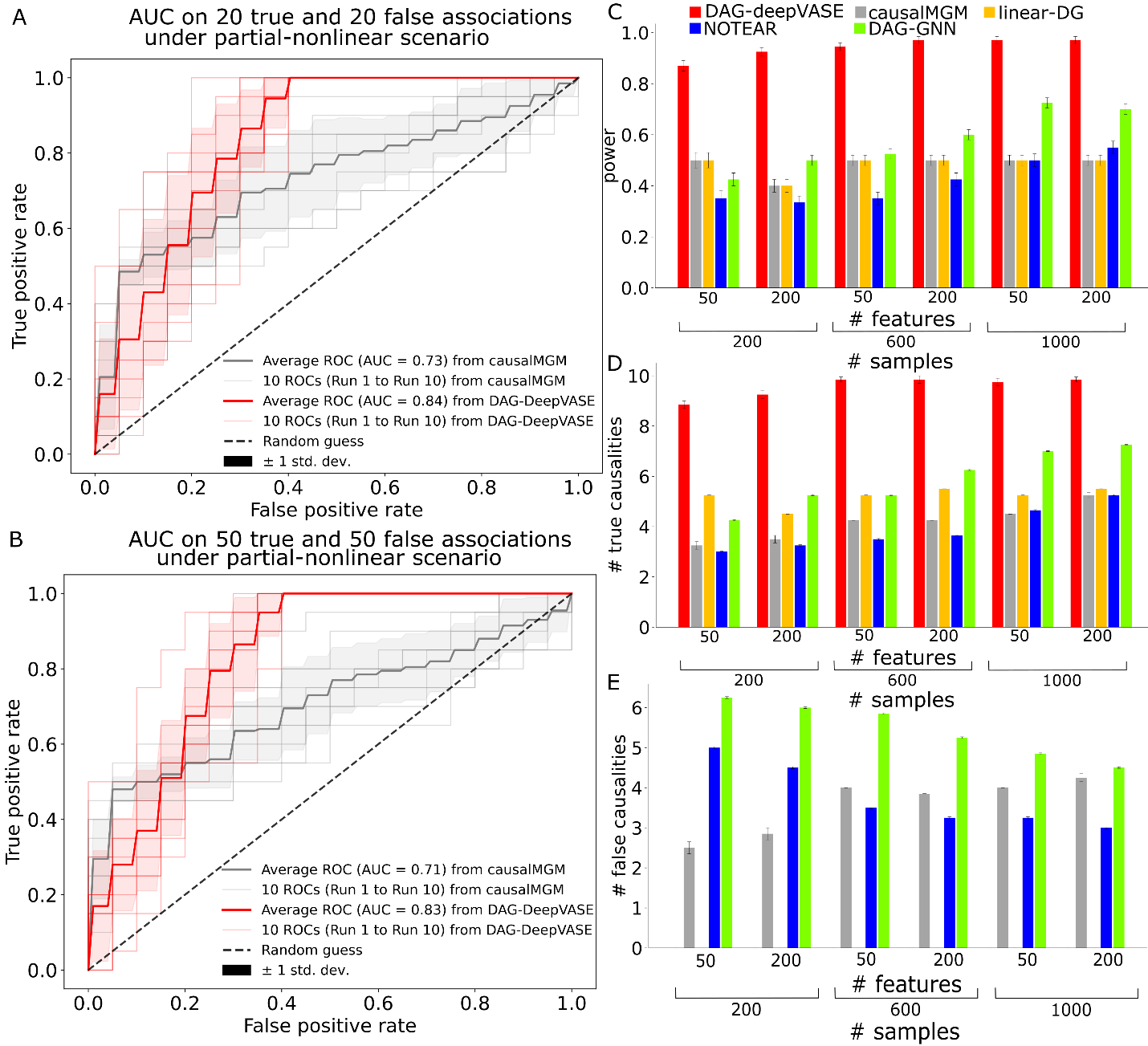
**

AUC estimated for DAG-deepVASE and causalMGM on **(A)** 20 true and false associations and **(B)** 40 true and false associations, both under complete-nonlinear scenarios. **(C)** Average number, and standard error (error bar), of true associations in the partial-nonlinear scenario identified by DAG-deepVASE (red), causalMGM (gray), linear-DG (yellow), NOTEAR (blue), and DAG-GNN (green) over 50 runs in various simulation scenarios, varying the number of features and sample sizes. Average number, and standard error (error bar), of **(D)** true causalities and **(E)** false causalities over 50 runs. DAG-deepVASE and linear-DG did not identify any false causalities.

Supplemental Figure 2_ Supplementary Material.


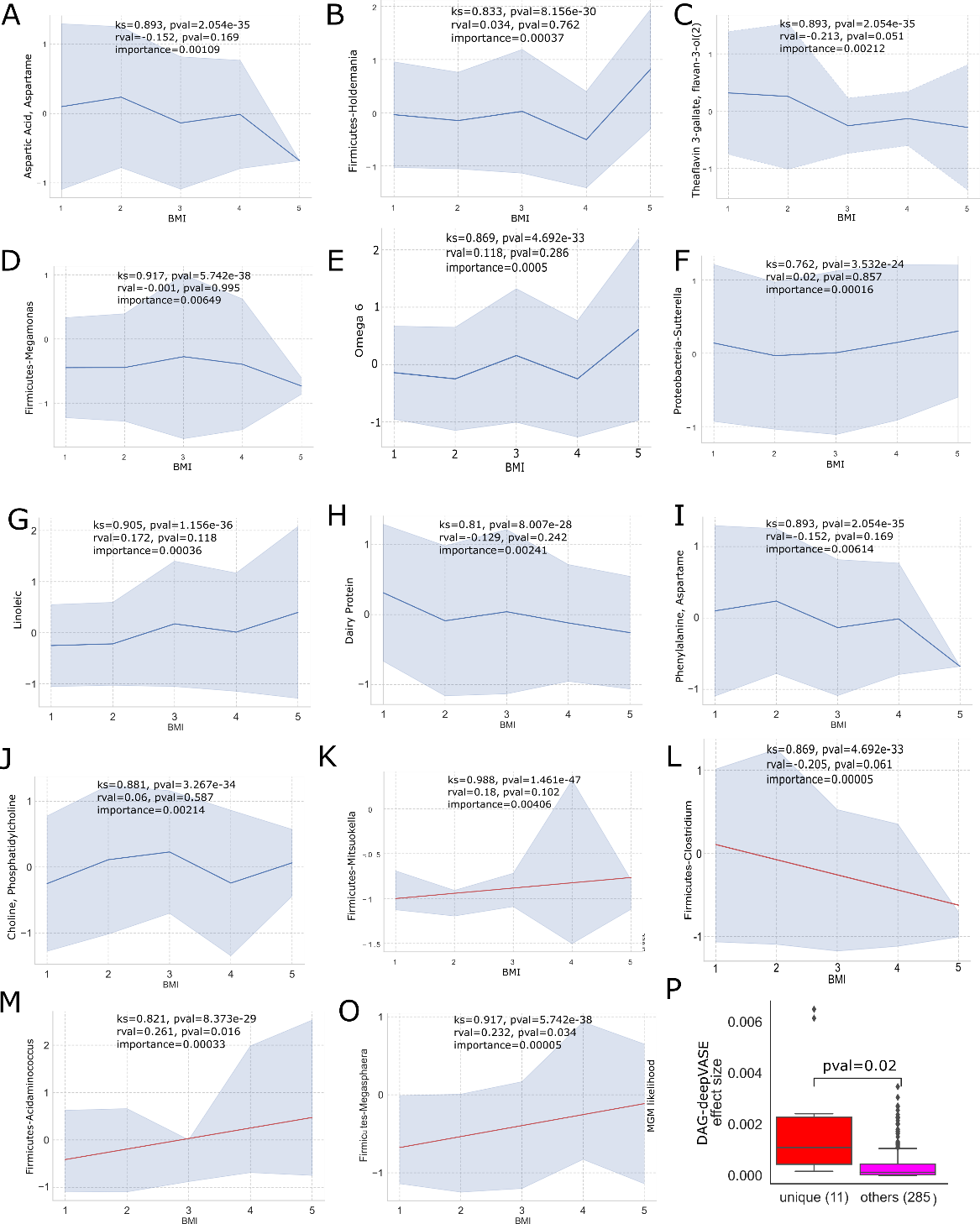


**(A-J)** Variable values against the BMI value window (1~5) that are identified as a nonlinear association to BMI **(K-O)** Variable values against the BMI value window (1~5) that are identified as a linear association to BMI. In the figures, KS is Kolmogorov-Smirnov (KS) test statistic, p-value is estimated from the KS test, rval is from a linear regression model, pval is from the linear regression, and importance is measured in DAG-deepVASE. The gray area indicates 95% confidence intervals, the blue line indicates median values, and the red line represents a linearly regressed line. P-value for linear fit is calculated from a permutation test with R^2^ (Methods) **(P)** Effect size estimated by DAG-deepVASE for 16 validated factors and 285 other factors to BMI.

Supplemental Figure 3_Supplementary Material.

**
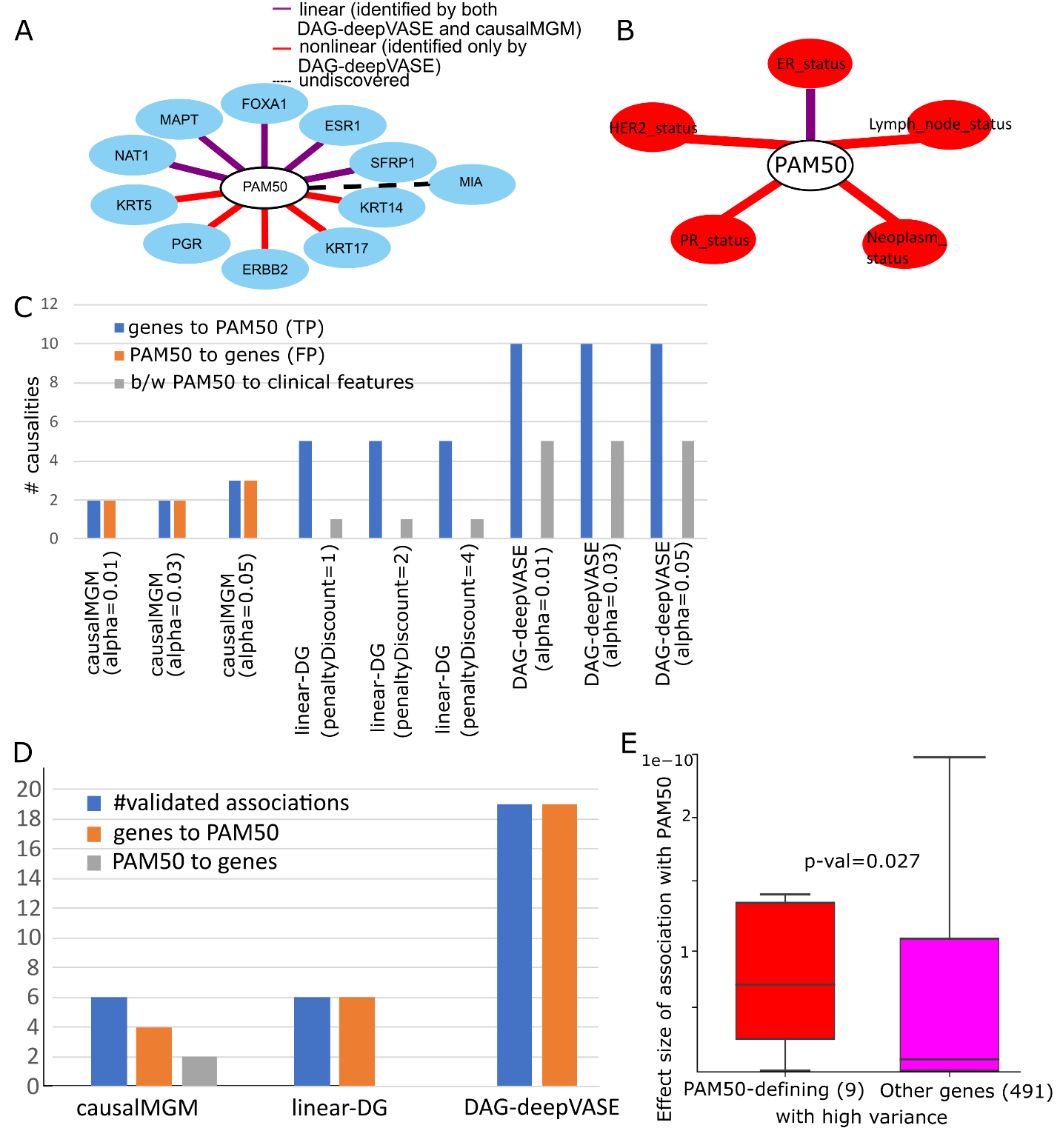
**

**(A)** PAM50-defining genes associated with the PAM50 status of patients identified by both DAG-deepVASE and causalMGM (purple) or uniquely by DAG-deepVASE (red). Both DAG-deepVASE and causalMGM could not identify a PAM50-defining gene (MIA) in a dotted line. **(B)** clinical features (e.g., hormone status) of the breast cancer samples associated with PAM50 that are identified by both DAG-deepVASE and causalMGM (purple) or uniquely by DAG-deepVASE (red). **(C)** Number of causalities identified by causalMGM, linear-DG, and DAG-deepVASE run with various parameter settings. **(D)** Number of validated associations (blue), causalities identified from genes to PAM50 status (orange) or from PAM50 status to genes (gray) when 20 PAM50 genes are run on DAG-deepVASE. **(E)** Effect size estimated by DAG-deepVASE for 9 PAM50-defining genes and 491 other genes in the 500 genes with highest expression variance.

Supplemental Figure 4_Supplementary Material.


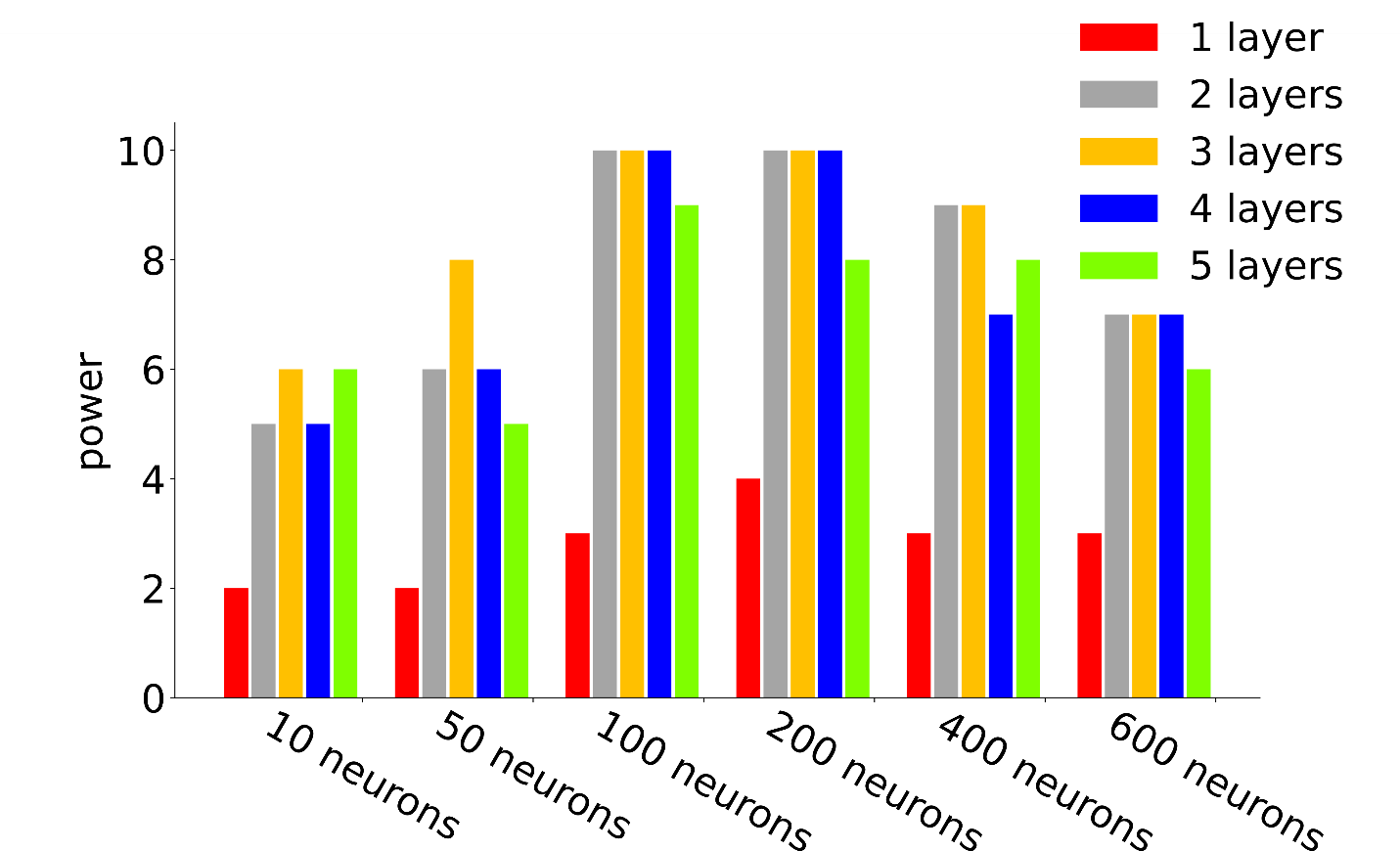


Average number, and standard error (error bar) of DAG-deepVASE for 10 true associations generated in the complete-nonlinear scenario for 1,000 samples with 190 false associations. To evaluate the model sufficiency, DAG-deepVASE was implemented with various numbers of neuron layers (1~5 layers) and various numbers of neurons (10, 50, 100, 200, 400, and 600 neurons) in each layer.
