# Supplementary material for: Deep neural networks with knockoff features identify nonlinear causal relations and estimate effect sizes in complex biological systems
Source: Gigascience. 2023 Jul 3;12:giad044. doi: 10.1093/gigascience/giad044 (PMC10316696; doi:10.1093/gigascience/giad044)
Supplement: giad044_Response_to_Reviewer_Comments_Original_Submission [file giad044_response_to_reviewer_comments_original_submission.pdf]

Author's Response To Reviewer Comments

Close

Summary: First, we would like to appreciate that Reviewers acknowledged the significance and novelty of our findings. For example:
“a topic that has been of interest to the scientific community working in various fields of systems biology for the past two decades” (Reviewer #1)
“The article is therefore certainly of interest, and also presents good results” (Reviewer #1)
“I think the proposed method is innovative” (Reviewer #2)
“the experiment results were convincing” (Reviewer #2)

Second, the Editor and both Reviewers appeared to have read our manuscript carefully to have constructive suggestions. Please see below for a detailed point-by-point response.

Editor’s comments
In addition, prior to review we had discussed the point that the sepsis data is not available for other scientists, which is problematic regarding our open science principles.
You indicated that you would be able to show a kind of limited example, and I encourage you to do this,

Response:
We agreed that a limited example data set should be provided to ensure reproducibility of our findings. We added the description of what we did regarding this point under Availability of Supporting Data as follows.
“The entire data are available upon request and after taking due steps for the rights and welfare of human research subjects involved in the study (regarding the Institutional Review Board review). However, to ensure reproducibility of our findings, we uploaded a down-sampled (70%) version of our data sets for the interactions of SIRS on the data repository site described below. The interactions of SIRS form the basis of our novel findings and include SIRS with heart rate, CRP (C-reactive protein), IFN-γ (interferon gamma), CNS (central nervous system) dysfunction, and IL (interleukin)-22. We ensured that our findings are reproduced using this data set.”

and to also provide as much detail as possible to clarify the data processing steps and analyses.
Response:
Thanks to your suggestions, we added more details of the preprocessing steps for each data. For the sepsis data, we added “to address the right-skewness of the clinical features, we adapted the log transformation (log10) on the values.” For the gut microbiome data, we realized that, although we cited a previous study that describes the data processing steps we took, we did not explain about the experiment in our manuscript. Thus, we added “They (healthy donors) were between the ages of 18 and 40 and required to be free from any chronic gastrointestinal disease, cardiac disease, diabetes mellitus or immunodeficiency diseases, to have a normal bowel frequency (between once every 2 days and 3 times per day), to have body mass index (BMI) between 18.5 and 35. They had not taken antibiotics within 6 months prior to enrollment, proton pump inhibitors, H2 receptor antagonists, tricyclic antidepressants, narcotics, anticholinergic medications, laxatives, or antidiarrhea medications within 4 weeks of enrollment, or NSAIDs, dietary supplements, or antacids within 2 weeks prior to enrollment.” For the breast cancer data, we added the details of the normalization scheme for the gene expression values, which is the following.
“The gene expression RNAseq section in the Xena website is the level 3 data estimates in log2(x+1) transformed RSEM normalized count obtained from the TCGA data coordination center. The University of North Carolina TCGA genome characterization center experimentally measured the gene expression profile using the Illumina HiSeq 2000 RNA Sequencing platform. Since we selected genes based on the expression variation, we did not use gene expression data with further normalization in the Xena website, such as pancan normalization or percentile normalization.”

Reviewer #1

1. The paper proposes DAG-deepVASE for the inference of causal networks, a topic that has been of interest to the scientific community working in various fields of systems biology for the past two decades. The literature on the subject is vast, and the several current software performs more than well, although there are still several unresolved problems, including the accuracy in identifying causal relationships from non-linear dynamics, and, especially, in certain types of biological network (e.g. gene regulatory networks) the presence of a high rate of false positives.

The article is therefore certainly of interest, and also presents good results. However, it requires some revision in the "Introduction" and some additions in the "Algorithms" that prove the validity and/or justify the mathematical tools used.

Here below, my comments and questions.

1) For the identification of causality directions in non-linear interaction it proposes the use of artificial intelligence tools and concepts such as DNNs. These methodologies are now of increasingly wide and ubiquitous application. In particular, as far as I know there are other proposals and software in this direction (in published papers, preprints, software repositories, etc.), such as:

- https://www.nature.com/articles/s42256-020-0218-x
- https://arxiv.org/abs/2011.12508
- https://www.arxiv-vanity.com/papers/2011.12508/
- https://arxiv.org/pdf/2109.10817.pdf
- https://towardsdatascience.com/fantastic-activation-functions-and-when-to-use-them-481fe2bb2bde
- https://github.com/kochbj/Deep-Learning-for-Causal-Inference
- https://www.deepmind.com/tags/causal-inference

In the light of this the authors should better justify their sentence

"… we developed the first computational method that learns both linear and nonlinear causal relationships, named causal Directed Acyclic Graphs using deep-learning VAriable SElection (DAG-deepVASE)."
Response:
Thank you for suggesting this important update. Now with an extensive review that includes your suggested papers above, we updated our claim as “we developed the first computational method that explicitly learns nonlinear causal relationships”. To explain why it is the first one to explicitly learn nonlinear causal relationships, we updated texts in Introduction as below, 1) introducing other methods including DNN methods, 2) their common limitations, and 3) how we approached to address the limitations. Then, in Result, we added the performance evaluation of existing DNN methods. Finally, in Discussion, we discussed why we think DAG-deepVASE’s approach outperforms the other DNN methods.

First, the following texts are added to introduce other methods, including the DNN methods. “Score-based algorithms generally formulate the causal learning problem as a search problem to optimize a certain score function with respect to an unknown directed acyclic graph (DAG) and the input data. To solve this problem and identify the DAG, a challenge can be the intractable search space that increases with a complexity super exponential to the number of the input variables. Thus, a group of methods have been developed to efficiently navigate the search space. Previously, this problem was addressed with additional structure assumptions, e.g., in terms of tree width [28], number of variables [29], or ancestral constraints [30]. While they were designed to shrink the intractable search space with the assumptions, methods can also be developed to expand the search space and efficiently navigate it. In that regard, a recent breakthrough formulates the problem as a continuous optimization with a structural constraint that ensures acyclicity [31] and spurs further development of deep neural network (DNN) models. For example, Yu et al. proposed a deep generative model and apply a variant of the structural constraint to learn the DAG [32] and Zheng et al. generalized this framework so various approximations can be used for search (NOTEARS) [33] , including neural networks.”

Second, the following texts are added to identify their common limitation. “Some of the nonlinear associations may be revealed in the existing approaches of navigating the expanded search space. However, since the search space connects DAGs of different combination of causal relationships, they are

designed to identify a particular combination of causal relationships by an optimization criterion and thus does not explicitly identify each causal relationship as nonlinear.”

Third, to clarify the novelty of DAG-deepVASE addressing the limitation, we added the following text in the first Result section. “DAG-deepVASE further identifies nonlinearly associated variables by developing a set of deep neural network (DNN) models, each with one of the input variables as the outcome and all the others as the dependent variables (Figure 1B). Note that DAG-deepVASE places the DNN component between the outcome and the dependent variables while other methods place the DNN component between the whole input data and the DAG to search for. Thus, DAG-deepVASE is designed to explicitly identify each variable relationship as nonlinear.”

In the Result section, we performed our simulation experiments with the other DNN-based causal inference methods. The results demonstrate that DAG-deepVASE consistently outperforms the other DNN methods (Fig. 2C, D, E and S. Fig. 1C, D, E).

As to why we think DAG-deepVASE outperforms other DNN-based methods, we added our thoughts in Discussion as follows. “In this manuscript, to identify individual associations as nonlinear, we took a different approach than other DNN methods by putting the DNN component between variables and ensuring acyclicity separately. The results showed that this approach performs better at identifying the causal DAG. For example, in our simulation experiments, NOTEARS and DAG-GNN showed generally less than 50% of power compared to DAG-deepVASE. In addition, our experiments suggest that, the approach of navigating the search space, may perform better when more samples are collected to construct a more comprehensive search space. For example, NOTEARS and DAG-GNN identified more true causalities when more samples are input (Fig. 2C, 2D and S. Fig.1C, 1D), though the improvement seems to come at the expense of high false positives (Fig. 2E and S. Fig.1E).”

2. A few lines later, the authors state that their contribution is an extension of already existing contributions and ideas:

"While the knockoff framework has been recently coupled with a deep-learning approach to estimate the effect size for a variable selection context [9], [35], DAG-deepVASE extends this approach for causal inference for the first time."

The reader is somewhat confused by these statements. I therefore suggest that the authors better restructure the part of the introduction that contains them.
Response:
Thanks to the reviewer for pointing out this potentially misleading text. We updated the text as follows. “Previously, this architecture was used to control false positive rate in the context of variable selection [36]. In this work, we incorporate this architecture for the context of causal inference to measure the effect size for the first time.”

3. Also in the introduction, this sentence appears to be of rather obscure meaning:

"Since a regular DNN model cannot estimate the nonlinear effect size due to its black-box property."

I suggest the author to expand this sentence upon.
Response:
We thank the reviewer for helping us clarify our text. We updated the text into “Although estimating the effect size is important to design further clinical trials and/or experimental validations with strong drivers, it is not straightforward to estimate the effect size in a regular DNN approach since it entailed to summarize the edge weights across multiple layers.”

4. 2) Secondly, I remain quite doubtful that the approach proposed by the authors really distinguishes the concept of linear or non-linear association from the concept of causality.
The contributions made by the authors seem to be

a) the introduction of knockoff variables for the estimation of effect size.

The authors say that knockoff variable " …resemble the correlation structure of the input variables, but are conditionally independent of the outcome, given the input variables."

Then they explain: "This property of knockoff variables allows us to estimate how important the original association is in reference to the knockoff variables, leading to the effect size estimation."

b) Then, "after identifying both linear and non-linear associations, DAGdeepVASE determines their causal direction using a single metric to ensure causal inference
consistency between linear and nonlinear causalities."

The step at point a) is necessary to determine the strength of causal relations, but not causal directions, so that the methods at point b) has to be implemented to find the direction of causality. If so, I am doubtful about the appropriateness of step a). Conditional independence is formulated by conditional probability (as it can be understood on what is reported on pages 25, and 26). It is well known, that in general, conditional independence does not mean absence of causality. So, the question is if knockoff variables are actually used for the purpose to identify causal relationship from non-linear association.

I believe that these doubts can be dispelled if the authors revise and integrate the presentation of the mathematical part on this aspect, and, highlight also its limitations on this same aspect.

Response:
Thank you for pointing this out. We acknowledge that our previous description could be confusing. Our answer is that knockoff variables are used not to identify causal relationships, but to estimate the effect size of non-linear associations. We showed this in Figure 1B and discussed it in the first result section. For the goal of identifying causal relationship from non-linear association, we used DG as shown in Figure 1D. However, we agree with the reviewer that our mathematical part can be misleading. Particularly, under the section of Algorithm of DAG-deepVASE, "causal relation" was incorrectly used so we replaced it in the following text with "association". "suppose (x_i,x_k) is a correlation without causal relation. Then, the feature exchangeability (x_i,x_k,(x_k)̃) =^d (x_i,(x_k)̃,x_k) will hold and make their relationship measure | 〖RI〗 _ik | and |(〖RI〗 _ik)̃ | exchangeable, which will make S_ik= | 〖RI〗 _ik |-|(〖RI〗 _ik)̃ | to follow a distribution symmetric around 0. On the other hand, suppose (x_i,x_j) is a causal relation. Then, S_ij will indicate how deviated the relationship of (x_i, x_j) is compared to the null hypothesis, leading to estimation of the effect size."
The above paragraph is incorrect as the knockoff-based effect size does not indicate a causal relation. The updated text is as follows. "For example, suppose (x_i,x_k) is a pair without an association. Then, the feature exchangeability (x_i,x_k,(x_k)̃) =^d (x_i,(x_k)̃,x_k) will hold and make their relationship measure | 〖RI〗 _ik | and |(〖RI〗 _ik)̃ | exchangeable, which will make S_ik= | 〖RI〗 _ik |-|(〖RI〗 _ik)̃ | to follow a distribution symmetric around 0. On the other hand, suppose (x_i,x_j) is an associated pair. Then, S_ij will indicate how deviated the relationship of (x_i, x_j) is compared to the null hypothesis, leading to estimation of the effect size. It is important to note that S_ji indicates the nonlinear effect size, not of the strength or direction of the causal relation."
Also, we added the following sentence in Discussion as a limitation. "care needs to be taken in interpreting the nonlinear effect size as it does not indicate strength or direction of causal relations."

Reviewer #2
1. Learning causal structure is a long-standing and challenging problem in biomedicine. This paper proposed a deep learning method named DAG-deepVASE to learn non-linear causal relationships for complex biological systems. This method is a two-step approach: 1) identify linear and nonlinear variable associations and estimate the effect sizes and 2) infer causal relationships among the identified associations. The major innovation of this work lies in step 1: it used a deep neural network to capture nonlinear associations and incorporated a knockoff framework (developed by others in refs 9 and 35) to estimate the effect sizes. For step 2, this work adopted a score-based method called degenerate Gaussian score (DG) to learn causal directions. The authors compared their method to two baseline methods, causalMGM and DG, which can only identify linear associations. In evaluation experiments, the proposed methods outperformed the two baselines on one synthetic and three real datasets: it identified more true associations and causal relationships than the baselines. The authors also demonstrated that top

associations that have the largest effect sizes estimated by DAG-deepVASE are almost validated, with supporting evidence in the literature.

Overall, I think the proposed method is innovative and the experiment results were convincing, especially the results on real data. The paper is well-written and easy to follow. I have the following comments.

- Review of related work. In several places in the manuscript, the authors stated that the proposed method is the "first computational method that learns both linear and nonlinear causal relationships", but I am not entirely sure whether this is accurate. For example, in the machine learning community, deep learning methods for learning directed acyclic graph (DAG) structure of causal relationships have been widely studied in the last 3-4 years. I list a few notable papers and reviews here:

* Zheng, X., Aragam, B., Ravikumar, P. K., & Xing, E. P. (2018). Dags with no tears: Continuous optimization for structure learning. Advances in Neural Information Processing Systems, 31.
* Yu, Y., Chen, J., Gao, T., & Yu, M. (2019, May). DAG-GNN: DAG structure learning with graph neural networks. In International Conference on Machine Learning (pp. 7154-7163). PMLR.
* Kyono, T., Zhang, Y., & van der Schaar, M. (2020). CASTLE: regularization via auxiliary causal graph discovery. Advances in Neural Information Processing Systems, 33, 1501-1512.
* Luo, Y., Peng, J., & Ma, J. (2020). When causal inference meets deep learning. Nature Machine Intelligence, 2(8), 426-427.
* Vowels, M. J., Camgoz, N. C., & Bowden, R. (2021). D'ya like DAGs? A survey on structure learning and causal discovery. ACM Computing Surveys (CSUR).

Several deep learning-based methods for DAG learning were proposed in the above papers and they are able to learn nonlinear associations/causalities. The authors can review those papers and discuss whether they are applicable to the problem studied in this work.
Response:
Thank you for suggesting this important update. Now with an extensive review that includes your suggested papers above, we updated our claim as “we developed the first computational method that explicitly learns nonlinear causal relationships”. To explain why it is the first one to explicitly learn nonlinear causal relationships, we updated texts in Introduction as below, 1) introducing other methods including DNN methods, 2) their common limitations, and 3) how we approached to address the limitations. Then, in Result, we added the performance evaluation of existing DNN methods. Finally, in Discussion, we discussed why we think DAG-deepVASE’s approach outperforms the other DNN methods.

First, the following texts are added to introduce other methods, including the DNN methods. “Score-based algorithms generally formulate the causal learning problem as a search problem to optimize a certain score function with respect to an unknown directed acyclic graph (DAG) and the input data. To solve this problem and identify the DAG, a challenge can be the intractable search space that increases with a complexity super exponential to the number of the input variables. Thus, a group of methods have been developed to efficiently navigate the search space. Previously, this problem was addressed with additional structure assumptions, e.g., in terms of tree width [28], number of variables [29], or ancestral constraints [30]. While they were designed to shrink the intractable search space with the assumptions, methods can also be developed to expand the search space and efficiently navigate it. In that regard, a recent breakthrough formulates the problem as a continuous optimization with a structural constraint that ensures acyclicity [31] and spurs further development of deep neural network (DNN) models. For example, Yu et al. proposed a deep generative model and apply a variant of the structural constraint to learn the DAG [32] and Zheng et al. generalized this framework so various approximations can be used for search (NOTEARS) [33] , including neural networks.”

Second, the following texts are added to identify their common limitation. “Some of the nonlinear associations may be revealed in the existing approaches of navigating the expanded search space. However, since the search space connects DAGs of different combination of causal relationships, they are designed to identify a particular combination of causal relationships by an optimization criterion and thus does not explicitly identify each causal relationship as nonlinear.”

Third, to clarify the novelty of DAG-deepVASE addressing the limitation, we added the following text in the

first Result section. “DAG-deepVASE further identifies nonlinearly associated variables by developing a set of deep neural network (DNN) models, each with one of the input variables as the outcome and all the others as the dependent variables (Figure 1B). Note that DAG-deepVASE places the DNN component between the outcome and the dependent variables while other methods place the DNN component between the whole input data and the DAG to search for. Thus, DAG-deepVASE is designed to explicitly identify each variable relationship as nonlinear.”

In the Result section, we performed our simulation experiments with the other DNN-based causal inference methods. The results demonstrate that DAG-deepVASE consistently outperforms the other DNN methods (Fig. 2C, D, E and S. Fig. 1C, D, E). We will discuss this in detail below.

As to why we think DAG-deepVASE outperforms other DNN-based methods, we added our thoughts in Discussion as follows. “In this manuscript, to identify individual associations as nonlinear, we took a different approach than other DNN methods by putting the DNN component between variables and ensuring acyclicity separately. The results showed that this approach performs better at identifying the causal DAG. For example, in our simulation experiments, NOTEARS and DAG-GNN showed generally less than 50% of power compared to DAG-deepVASE. In addition, our experiments suggest that, the approach of navigating the search space, may perform better when more samples are collected to construct a more comprehensive search space. For example, NOTEARS and DAG-GNN identified more true causalities when more samples are input (Fig. 2C, 2D and S. Fig.1C, 1D), though the improvement seems to come at the expense of high false positives (Fig. 2E and S. Fig.1E).”

2. - Performance evaluation. The proposed method has achieved impressive results compared to the baselines in the evaluation experiments. Presumably, the major reason was that the proposed method was able to identify nonlinear associations but the two baselines were only able to identify linear associations. I was curious about the possibility of comparing the proposed method to baselines that can identify nonlinear associations (e.g., some methods from the papers listed above).
Response:

Thanks for this suggestion, we extended the simulation experiments to include two established DNN methods, NOTEARS and DAG-GNN, which you mentioned above, and updated several places of our text with the result as follows. In terms of power, “DAG-deepVASE shows a two-fold higher power than the other methods by identifying more than 90% of the true associations in most simulation scenarios (Fig. 2C). Interestingly, while DAG-GNN performs slightly better than linear approaches, causalMGM and linear-DG, in terms of power and sensitivity, NOTEARS performs the worst in all scenarios in general.”

In terms of the number of true causalities, “Further, the other DNN methods also identified less than 50% of the true causalities than DAG-deepVASE in most scenarios.”

And, in terms of the number of false causalities, “both DNN methods, NOTEARS and DAG-GNN, suffer from the highest number of false causalities.”

We excluded NOTEAR and DAG-GNN in analyzing real datasets since they identified high rates of false positive causalities in simulation studies.

3. - Reproducibility. In the BMI task, 8 nutrient intakes and bacteria genera that are known to affect BMI were selected to evaluate the methods. Similarly, in the breast cancer task, the top 10 genes with the largest variance from PAM50 genes were selected to evaluate the methods. I would like to see some analyses or discussions regarding the reproducibility of those experiments. For example, would we observe similar results if we switch to another 8 nutrient intakes and bacteria genera for the BMI task or consider the top 20 genes in the breast cancer task?
Response:
To respond to this insightful comment, we chose to extend our breast cancer data analysis instead of the BMI data analysis since the extension would be more systematic as written as follows. “To ensure reproducibility of this finding, we further selected the top 20 genes from PAM50 genes with the largest variance in the data and evaluated the methods on the genes. In identifying true associations, DAG-deepVASE identified 19 associations out of 20 (95.5%) while both causalMGM and linear-DG identified 6 of

them (30%) (S. Fig. 3D). And, in learning the causal directions, DAG-deepVASE identified 19 true causalities from all the identified associations, while causalMGM identified 4 true and 2 false causalities out of the 6 associations and direct-DG identified 6 true causalities from all the identified associations (S. Fig. 3D). Altogether, DAG-deepVASE outperforms causalMGM and linear-DG in identifying true associations, learning true causalities, and differentiating false causalities in the breast cancer data.”

4. - Ablation analyses. The proposed method relies on the nonlinear deep neural network (DNN) to capture the nonlinear associations. A natural question is how nonlinear or complex the DNN should be to capture the nonlinear associations. For example, how would the results change as a function of the number of layers or hidden units in each layer?
Response:
Thanks to this helpful suggestion, we studied the nonlinearity and complexity of our DNN method and added the following text under Methods “we performed experiments with various numbers of neuron layers (1~5 layers) and various numbers of neurons (10, 50, 100, 200, 400, and 600 neurons) in each layer on the simulation data (10 and 190 features in true and false causal relation to the outcome, respectively, generated for 1,000 samples) (S. Fig. 4). Our experiments demonstrate that, to run on the simulation data, which consist of 100 features, DNN models of multiple layers of 100 neurons, which is our current setting, perform the best.”

Minor points:
5. - Please unify the citation format. For example, on page 15, ref 64 was labeled as [64] but ref 63 was labeled as a superscript.
Response:

Thank you for catching this oversight. I believe we have corrected this issue in the updated manuscript.

Close
