# Supplementary material for: Deep neural networks with knockoff features identify nonlinear causal relations and estimate effect sizes in complex biological systems
Source: Gigascience. 2023 Jul 3;12:giad044. doi: 10.1093/gigascience/giad044 (PMC10316696; doi:10.1093/gigascience/giad044)
Supplement: giad044_Response_to_Reviewer_Comments_Revision_1 [file giad044_response_to_reviewer_comments_revision_1.pdf]

Author's Response To Reviewer Comments

Close

Below discussion, revision figures and tables are also in reviewersComments_ver2.docx submitted under Personal Cover.

Summary: First, we would like to appreciate that Reviewers are satisfied with our comments in the revision and now have a more positive view regarding the merits of our manuscript. Second, we also appreciate the follow-up comments. Please see below for detailed point-by-point responses.

Editor's comments
C1: please pay particular attention to the reproducibility issue mentioned by reviewer 2.
Response:
To the comment, we explained why the experiment to predict BMI with other gut microbiota would be not as systematic as the breast cancer experiment. Though not as systematic, we also performed the experiment with other gut microbiota (R. Table 1 and R. Fig. 1), which consistently shows the outperformance of DAG-deepVASE to other methods. Please see our response below for details.

C2: Regarding reviewer 1's remark that more context is needed, please have a look at the literature mentioned by the referee and if there is previous work that is directly related and relevant to your manuscript please cite and discuss it.
Response:
Thanks to the suggestion, we added another section in the Background that reviews most of the literature the referee mentioned. Please see our response below for details.

Reviewer 1:
C1: I appreciate the efforts of the authors in making more rigourous and clear the mathematical methods.

I indicate a further necessary additions since the topic of causal inference is a very large and long-standing problem, which a large body of literature recently proposes to address with artificial intelligence (AI) methods. There is also a wide literature about causal infernce not performed with artificial intelligence methods, that should be mentioned and whose difference with AI methods should be highlighted.
I recommend to extend a bit the literature references, e.g. (but not limited to) with the following contributions:

- https://www.ncbi.nlm.nih.gov/pmc/articles/PMC6536645/
- https://journals.plos.org/ploscompbiol/article?id=10.1371/journal.pcbi.1009803
- https://journals.plos.org/plosone/article?id=10.1371/journal.pone.0150611
- https://www.ncbi.nlm.nih.gov/pmc/articles/PMC9581010/
- https://link.springer.com/article/10.1007/s10208-022-09581-9
- https://www.nature.com/articles/s41598-017-08582-x

Response:
Thanks to your suggestion, we added the following text in our Background section. "Previously, causal inference methods have been successfully used to provide insights into molecular mechanisms and predict treatment effects. First, to provide insights into molecular mechanisms (e.g., transcriptional regulatory relationship between genes), methods have been developed to integrate multiple types of data where the direction of effect is known from one type to another (e.g., from DNA variants to gene expression). Developments in this approach utilized both score-based (1, 2) and constraint-based (3–6) algorithms. For example, MRPC uses PC to examine a set of causal relationships between DNA variants and gene expression information implied by the principle of mendelian randomization (PMR). Second, to predict treatment effects, causal inference was done using multiple intervention trial or experiment data (e.g., RNAi-based gene knockout experiments). For example, conservative local causal discovery (CLCD) tests

conditional (in)dependence among multiple entities (e.g., proteins) across experiments (7) and BACKSHIFT evaluates particular causal scenarios shared across experiments using a linear causal model (8). Treatment effect can also be predicted based on the relationship of the input samples with other samples for which treatment effects are known. To this end, causal k-nearest neighbor algorithm (causal KNN) estimates the effect based on the nearest neighbors with known treatment effects. Similarly, causal random forest attempts to identify neighbors after recursively partitioning the covariate space through creating a set of decision trees. While they successfully identified biologically meaningful or clinically reasonable causal relations in various validation experiments, they are not necessarily relying on artificial intelligence (AI) methods and tend to test independence for a limited number of entities or based on naïve assumptions on the relationships among data points."

Reviewer 2:
C1: The authors have addressed most of my comments. However, I have two questions below.
- Review of related work. The authors have revised their claim as "the first computational method that explicitly learns nonlinear causal relationships". It was not clear to me what "explicitly" mean here. I'd suggest the authors elaborate more and discuss why previous methods were "implicit".
Response:
We agree that it is important to further clarify this important point. So, we updated our previous text to "However, the (existing DNN) methods utilize the DNN component to nonlinearly navigate the search space over various DAGs while optimizing an optimality score across all the relationships in a DAG as has been done previously. Generally, the optimality scores are based on a likelihood model with product terms to represent the variable relationships. For example, as the optimality score, both DAG-GNN and NOTEARS can use the BIC score that select the product terms to determine significant variable relationships. A product term of two variables assumes that the relationship between the variables is additive and proportional, meaning that the effect of one variable on the other is assumed to be constant across all levels of the other variable. Since the constant effect is satisfied only in linear relationships, the methods based on such optimality scores are designed to consider only linear relationships. In other words, as a DNN component is to address nonlinearity, existing methods use the DNN component to address nonlinearity in how various DAGs are searched through, not to address nonlinearity in each relationship. In this sense, they do not explicitly identify each causal relationship as nonlinear."
On the other hand, "to identify each causal relationship as nonlinear, DAG-deepVASE puts a deep neural network (DNN) model between each potential causal relationship." Thus, DAG-deepVASE explicitly learns nonlinear relationships.

C2: Reproducibility. In response to my question regarding the reproducibility of the proposed method on the BMI dataset, the author mentioned that "we chose to extend our breast cancer data analysis instead of the BMI data analysis since the extension would be more systematic". However, I am not fully sure why it was infeasible (or not systematic) to conduct a reproducibility analysis of results on the BMI dataset. I had this question because it was only mentioned in the manuscript that "we selected the same number (8) of nutrient intakes and bacteria genera" -- it was unclear why the specific number (8) of nutrient intakes was used. Are there only 8 nutrient intakes that are known to affect BMI, or there are more but only 8 were selected? If it was the latter case, how were the 8 selected (randomly or handpicked)? I think the results would be more convincing if the authors can show the proposed method would still perform the best if we select some number of other nutrient intakes as the positive variables in evaluations. If that is not doable, I would like to see some discussions/justifications why it is infeasible to perform the reproducibility analysis, and also more details about the particular experiment the authors have performed already (e.g., how were the 8 intakes chosen, and why 8, etc.).
Response:
We clarified several points raised by the reviewer as follows. The first point regards why the BMI data analysis extension would be less systematic than breast cancer data extension. To me, a systematic extension means another experiment with more entities selected from the same data set where other parameters are kept the same as before. Since PAM50 genes are tightly defined (9), we could select more PAM50 genes to test in the same TCGA breast tumor samples simply by going down on the gene list ranked by the expression variance estimated from the samples. On the other hand, since BMI predicting gut bacteria is not well defined, we need to select more of them by searching through other data sets. In that sense, I viewed the BMI data extension less systematic.
However, as the reviewer pointed out, it is definitely feasible to test on other BMI-predicting gut bacteria

collected from other studies. So, we searched research articles with keyword “gut bacteria affecting BMI”, from which we collected 14 other gut bacteria that affect BMI and obesity (R. Table 1). Among the 14 gut bacteria, DAG-deepVASE identified associations in eight of them (57.1%) while both linear-DG and causalMGM identified two (14.3%) (R. Fig. 1). Further, in determining the causal direction, DAG-deepVASE identified all causal directions from the bacteria to BMI and linear-DG identified the same directions in the two associations it identified. However, causalMGM identified 0 causal directions after removing both associations in the step of testing the conditional independent relationship. This result reproducibly demonstrates that 1) the causal relationship from gut microbiome to BMI is generally nonlinear and 2) DAG-deepVASE consistently outperforms other methods in identifying the nonlinear relationship.
Regarding how we chose 8 gut bacteria and 8 nutrient intakes for testing, we added the following text in the manuscript. “we selected the 16 features since they were previously suggested to have nonlinear associations with BMI by a DNN-based variable selection method (10).”

R. Table 1. 14 gut bacteria genera validated to affect BMI (or obesity) in literature.

phylum	genus	Identified in DAG-deepVASE (Associations /Causalities)	Identified in Linear-DG (Associations /Causalities)	Identified in causalMGM (Associations /Causalities)	Citation
Firmicutes	Faecalibacterium	Yes/Yes	No/No	No/No	(11)
Firmicutes	Lactobacillus	No/No	No/No	No/No	(12)
Bacteroidetes	Prevotella	No/No	No/No	No/No	(13)
Verrucomicrobia	Akkermansia	No/No	No/No	No/No	(14)
Firmicutes	Blautia	Yes/Yes	Yes/Yes	Yes/No	(15)
Firmicutes	Roseburia	Yes/Yes	No/No	No/No	(16)
Bacteroidetes	Bacteroides	Yes/Yes	No/No	No/No	(17)
Bacteroidetes	Parabacteroides	No/No	No/No	No/No	(17)
Firmicutes	Eubacterium	Yes/Yes	No/No	No/No	(17)
Firmicutes	Gemella	No/No	No/No	No/No	(17)
Firmicutes	Ruminococcus	Yes/Yes	No/No	No/No	(17)
Firmicutes	Streptococcus	No/No	No/No	No/No	(17)
Firmicutes	Veillonella	Yes/Yes	Yes/Yes	Yes/No	(17)
Proteobacteria	Desulfovibrio	Yes/Yes	No/No	No/No	(17)

References

1. E. C. Neto, M. P. Keller, A. D. Attie, B. S. Yandell, CAUSAL GRAPHICAL MODELS IN SYSTEMS GENETICS: A UNIFIED FRAMEWORK FOR JOINT INFERENCE OF CAUSAL NETWORK AND GENETIC ARCHITECTURE FOR CORRELATED PHENOTYPES. Ann. Appl. Stat. 4, 320–339 (2010).
2. W. Kruijer, et al., Reconstruction of Networks with Direct and Indirect Genetic Effects. Genetics 214, 781–807 (2020).

3. A. Yazdani, A. Yazdani, A. Samiei, E. Boerwinkle, Generating a robust statistical causal structure over 13 cardiovascular disease risk factors using genomics data. J. Biomed. Inform. 60, 114–119 (2016).
4. A. Yazdani, A. Yazdani, A. Saniei, E. Boerwinkle, A causal network analysis in an observational study identifies metabolomics pathways influencing plasma triglyceride levels. Metabolomics 12, 104 (2016).
5. A. Yazdani, et al., Arachidonic acid as a target for treating hypertriglyceridemia reproduced by a causal network analysis and an intervention study. Metabolomics 14, 78 (2018).
6. A. Yazdani, et al., Genome analysis and pleiotropy assessment using causal networks with loss of function mutation and metabolomics. BMC Genomics 20, 395 (2019).
7. S. Triantafillou, et al., Predicting Causal Relationships from Biological Data: Applying Automated Causal Discovery on Mass Cytometry Data of Human Immune Cells. Sci. Rep. 7, 12724 (2017).
8. D. Rothenhäusler, C. Heinze, J. Peters, N. Meinshausen, BACKSHIFT: Learning causal cyclic graphs from unknown shift interventions. Adv. Neural Inf. Process. Syst. 2015-January, 1513–1521 (2015).
9. D. C. Koboldt, et al., Comprehensive molecular portraits of human breast tumours. Nature 490, 61–70 (2012).
10. Y. Y. Lu, Y. Fan, J. Lv, W. S. Noble, Deeppink: Reproducible feature selection in deep neural networks. Adv. Neural Inf. Process. Syst. 2018-Decem, 8676–8686 (2018).
11. M. Lopez-Siles, S. H. Duncan, L. J. Garcia-Gil, M. Martinez-Medina, Faecalibacterium prausnitzii: from microbiology to diagnostics and prognostics. ISME J. 11, 841–852 (2017).
12. M. Sanchez, et al., Effect of Lactobacillus rhamnosus CGMCC1.3724 supplementation on weight loss and maintenance in obese men and women. Br. J. Nutr. 111, 1507–1519 (2014).
13. F. Fava, et al., The type and quantity of dietary fat and carbohydrate alter faecal microbiome and short-chain fatty acid excretion in a metabolic syndrome "at-risk" population. Int. J. Obes. (Lond). 37, 216–223 (2013).
14. M. C. Dao, et al., Akkermansia muciniphila and improved metabolic health during a dietary intervention in obesity: relationship with gut microbiome richness and ecology. Gut 65, 426–436 (2016).
15. K. Hosomi, et al., Oral administration of Blautia wexlerae ameliorates obesity and type 2 diabetes via metabolic remodeling of the gut microbiota. Nat. Commun. 13, 4477 (2022).
16. K. Nie, et al., Roseburia intestinalis: A Beneficial Gut Organism From the Discoveries in Genus and Species. Front. Cell. Infect. Microbiol. 11, 757718 (2021).
17. V. Palmas, et al., Gut microbiota markers associated with obesity and overweight in Italian adults. Sci. Rep. 11, 5532 (2021).

Close
