# Supplementary material for: Deep neural networks with knockoff features identify nonlinear causal relations and estimate effect sizes in complex biological systems
Source: Gigascience. 2023 Jul 3;12:giad044. doi: 10.1093/gigascience/giad044 (PMC10316696; doi:10.1093/gigascience/giad044)
Supplement: giad044_Response_to_Reviewer_Comments_Revision_2 [file giad044_response_to_reviewer_comments_revision_2.pdf]

Author's Response To Reviewer Comments

Close

Dear Editor,

Thank you for reminding me of the requests.
I now added both bio.tools ID and RRID in the designated place in the manuscript and convert all data and code, including the one on page 30 into citations.
Please let me know if there is anything else I can do to help you proceed with our manuscript.

Best,
HJ

Close
