# Supplementary material for: Deep neural networks with knockoff features identify nonlinear causal relations and estimate effect sizes in complex biological systems
Source: Gigascience. 2023 Jul 3;12:giad044. doi: 10.1093/gigascience/giad044 (PMC10316696; doi:10.1093/gigascience/giad044)
Supplement: giad044_Response_to_Reviewer_Comments_Revision_3 [file giad044_response_to_reviewer_comments_revision_3.pdf]

Author's Response To Reviewer Comments

Close

I modified the manuscript according to your requests (removing citations from Abstract and adding another citation for our data deposited to the GigaDatabse.

Thanks,
HJ

Close
