# Supplementary material for: Deep neural networks with knockoff features identify nonlinear causal relations and estimate effect sizes in complex biological systems
Source: Gigascience. 2023 Jul 3;12:giad044. doi: 10.1093/gigascience/giad044 (PMC10316696; doi:10.1093/gigascience/giad044)
Supplement: giad044_Reviewer_1_Report_Original_Submission — Paola Lecca -- 10/30/2022 Reviewed [file giad044_reviewer_1_report_original_submission.pdf]

Reviewer Report

Title: Deep neural networks with knockoff features identify nonlinear causal relations and estimate effect sizes in complex biological systems

Version: Original Submission **Date:** 10/30/2022

Reviewer name: Paola Lecca

Reviewer Comments to Author:

The paper proposes DAG-deepVASE for the inference of causal networks, a topic that has been of interest to the scientific community working in various fields of systems biology for the past two decades. The literature on the subject is vast, and the several current software performs more than well, although there are still several unresolved problems, including the accuracy in identifying causal relationships from non-linear dynamics, and, especially, in certain types of biological network (e.g. gene regulatory networks) the presence of a high rate of false positives.The article is therefore certainly of interest, and also presents good results. However, it requires some revision in the "Introduction" and some additions in the "Algorithms" that prove the validity and/or justify the mathematical tools used.Here below, my comments and questions.1) For the identification of causality directions in non-linear interaction it proposes the use of artificial intelligence tools and concepts such as DNNs. These methodologies are now of increasingly wide and ubiquitous application. In particular, as far as I know there are other proposals and software in this direction (in published papers, preprints, software repositories, etc.), such as:- https://www.nature.com/articles/s42256-020-0218-x- https://arxiv.org/abs/2011.12508- https://www.arxiv-vanity.com/papers/2011.12508/- https://arxiv.org/pdf/2109.10817.pdf- https://towardsdatascience.com/fantastic-activation-functions-and-when-to-use-them-481fe2bb2bde- https://github.com/kochbj/Deep-Learning-for-Causal-Inference- https://www.deepmind.com/tags/causal-inference- …In the light of this the authors should better justify their sentence"… we developed the first computational method that learns both linear and nonlinear causal relationships, named causal Directed Acyclic Graphs using deep-learning VAriableSElection (DAG-deepVASE)."A few lines later, the authors state that their contribution is an extension of already existing contributions and ideas:"While the knockoff framework has been recently coupled with a deep-learning approach to estimate the effect size for a variable selection context [9], [35], DAG-deepVASE extends this approach for causal inference for the first time."The reader is somewhat confused by these statements. I therefore suggest that the authors better restructure the part of the introduction that contains them.Also in the introduction, this sentence appears to be of rather obscure meaning:"Since a regular DNN model cannot estimate the nonlinear effect size due to its black-box property."I suggest the author to expand this sentence upon.2) Secondly, I remain quite doubtful that the approach proposed by the authors really distinguishes the concept of linear or non-linear association from the concept of causality.The contributions made by the authors seem to bea) the introduction of knockoff variables for the estimation of effect size.The authors say that knockoff variable " …resemble the correlation structure of the input variables, but are conditionally independent of the outcome, given the input variables."Then they explain: "This property of knockoff variables allows us to estimate how important

the original association is in reference to the knockoff variables, leading to the effect size estimation."b) Then, "after identifying both linear and non-linear associations, DAGdeepVASE determines their causal direction using a single metric to ensure causal inferenceconsistency between linear and nonlinear causalities."The step at point a) is necessary to determine the strength of causal relations, but not causal directions, so that the methods at point b) has to be implemented to find the direction of causality. If so, I am doubtful about the appropriateness of step a). Conditional independence is formulated by conditional probability (as it can be understood on what is reported on pages 25, and 26). It is well known, that in general, conditional independence does not mean absence of causality. So, the question is if knockoff variables are actually used for the purpose to identify causal relationship from non-linear association.I believe that these doubts can be dispelled if the authors revise and integrate the presentation of the mathematical part on this aspect, and, highlight also its limitations on this same aspect.

Level of Interest

Please indicate how interesting you found the manuscript: Choose an item.

Quality of Written English

Please indicate the quality of language in the manuscript: Choose an item.

Declaration of Competing Interests

Please complete a declaration of competing interests, considering the following questions:

- Have you in the past five years received reimbursements, fees, funding, or salary from an organisation that may in any way gain or lose financially from the publication of this manuscript, either now or in the future?
- Do you hold any stocks or shares in an organisation that may in any way gain or lose financially from the publication of this manuscript, either now or in the future?
- Do you hold or are you currently applying for any patents relating to the content of the manuscript?
- Have you received reimbursements, fees, funding, or salary from an organization that holds or has applied for patents relating to the content of the manuscript?
- Do you have any other financial competing interests?
- Do you have any non-financial competing interests in relation to this paper?

If you can answer no to all of the above, write 'I declare that I have no competing interests' below. If your reply is yes to any, please give details below.

I declare that I have no competing interests

I agree to the open peer review policy of the journal. I understand that my name will be included on my report to the authors and, if the manuscript is accepted for publication, my named report including any attachments I upload will be posted on the website along with the authors' responses. I agree for my report to be made available under an Open Access Creative Commons CC-BY license (http://creativecommons.org/licenses/by/4.0/). I understand that any comments which I do not wish to be included in my named report can be included as confidential comments to the editors, which will not be published.

Choose an item.

To further support our reviewers, we have joined with Publons, where you can gain additional credit to further highlight your hard work (see: https://publons.com/journal/530/gigascience). On publication of this paper, your review will be automatically added to Publons, you can then choose whether or not to claim your Publons credit. I understand this statement.

Yes Choose an item.
