# Supplementary material for: Deep neural networks with knockoff features identify nonlinear causal relations and estimate effect sizes in complex biological systems
Source: Gigascience. 2023 Jul 3;12:giad044. doi: 10.1093/gigascience/giad044 (PMC10316696; doi:10.1093/gigascience/giad044)
Supplement: giad044_Reviewer_1_Report_Revision_1 — Paola Lecca -- 2/11/2023 Reviewed [file giad044_reviewer_1_report_revision_1.pdf]

Reviewer Report

Title: Deep neural networks with knockoff features identify nonlinear causal relations and estimate effect sizes in complex biological systems

Version: Revision 1 **Date:** 2/11/2023

Reviewer name: Paola Lecca

Reviewer Comments to Author:

I appreciate the efforts of the authors in making more rigourous and clear the mathematical methods.I indicate a further necessary additions since the topic of causal inference is a very large and long-standing problem, which a large body of literature recently proposes to address with artificial intelligence (AI) methods. There is also a wide literature about causal infernce not performed with artificial intelligence methods, that should be mentioned and whose difference with AI methods should be highlighted.I recommend to extend a bit the literature references, e.g. (but not limited to) with the following contributions:- https://www.ncbi.nlm.nih.gov/pmc/articles/PMC6536645/- https://journals.plos.org/ploscompbiol/article?id=10.1371/journal.pcbi.1009803- https://journals.plos.org/plosone/article?id=10.1371/journal.pone.0150611- https://www.ncbi.nlm.nih.gov/pmc/articles/PMC9581010/- https://link.springer.com/article/10.1007/s10208-022-09581-9- https://www.nature.com/articles/s41598-017-08582-x

Level of Interest

Please indicate how interesting you found the manuscript: Choose an item.

Quality of Written English

Please indicate the quality of language in the manuscript: Choose an item.

Declaration of Competing Interests

Please complete a declaration of competing interests, considering the following questions:

- Have you in the past five years received reimbursements, fees, funding, or salary from an organisation that may in any way gain or lose financially from the publication of this manuscript, either now or in the future?
- Do you hold any stocks or shares in an organisation that may in any way gain or lose financially from the publication of this manuscript, either now or in the future?
- Do you hold or are you currently applying for any patents relating to the content of the manuscript?

- Have you received reimbursements, fees, funding, or salary from an organization that holds or has applied for patents relating to the content of the manuscript?
- Do you have any other financial competing interests?
- Do you have any non-financial competing interests in relation to this paper?

If you can answer no to all of the above, write 'I declare that I have no competing interests' below. If your reply is yes to any, please give details below.

I declare that I have no competing interests

I agree to the open peer review policy of the journal. I understand that my name will be included on my report to the authors and, if the manuscript is accepted for publication, my named report including any attachments I upload will be posted on the website along with the authors' responses. I agree for my report to be made available under an Open Access Creative Commons CC-BY license (http://creativecommons.org/licenses/by/4.0/). I understand that any comments which I do not wish to be included in my named report can be included as confidential comments to the editors, which will not be published.

Choose an item.

To further support our reviewers, we have joined with Publons, where you can gain additional credit to further highlight your hard work (see: https://publons.com/journal/530/gigascience). On publication of this paper, your review will be automatically added to Publons, you can then choose whether or not to claim your Publons credit. I understand this statement.

Yes Choose an item.
