# Supplementary material for: Deep neural networks with knockoff features identify nonlinear causal relations and estimate effect sizes in complex biological systems
Source: Gigascience. 2023 Jul 3;12:giad044. doi: 10.1093/gigascience/giad044 (PMC10316696; doi:10.1093/gigascience/giad044)
Supplement: giad044_Reviewer_2_Report_Original_Submission — Yunan Luo -- 10/31/2022 Reviewed [file giad044_reviewer_2_report_original_submission.pdf]

Reviewer Report

Title: Deep neural networks with knockoff features identify nonlinear causal relations and estimate effect sizes in complex biological systems

Version: Original Submission **Date:** 10/31/2022

Reviewer name: Yunan Luo

Reviewer Comments to Author:

Learning causal structure is a long-standing and challenging problem in biomedicine. This paper proposed a deep learning method named DAG-deepVASE to learn non-linear causal relationships for complex biological systems. This method is a two-step approach: 1) identify linear and nonlinear variable associations and estimate the effect sizes and 2) infer causal relationships among the identified associations. The major innovation of this work lies in step 1: it used a deep neural network to capture nonlinear associations and incorporated a knockoff framework (developed by others in refs 9 and 35) to estimate the effect sizes. For step 2, this work adopted a score-based method called degenerate Gaussian score (DG) to learn causal directions. The authors compared their method to two baseline methods, causalMGM and DG, which can only identify linear associations. In evaluation experiments, the proposed methods outperformed the two baselines on one synthetic and three real datasets: it identified more true associations and causal relationships than the baselines. The authors also demonstrated that top associations that have the largest effect sizes estimated by DAG-deepVASE are almost validated, with supporting evidence in the literature.Overall, I think the proposed method is innovative and the experiment results were convincing, especially the results on real data. The paper is well-written and easy to follow. I have the following comments.Major comments:- Review of related work. In several places in the manuscript, the authors stated that the proposed method is the "first computational method that learns both linear and nonlinear causal relationships", but I am not entirely sure whether this is accurate. For example, in the machine learning community, deep learning methods for learning directed acyclic graph (DAG) structure of causal relationships have been widely studied in the last 3-4 years. I list a few notable papers and reviews here:* Zheng, X., Aragam, B., Ravikumar, P. K., & Xing, E. P. (2018). Dags with no tears: Continuous optimization for structure learning. Advances in Neural Information Processing Systems, 31.* Yu, Y., Chen, J., Gao, T., & Yu, M. (2019, May). DAG-GNN: DAG structure learning with graph neural networks. In International Conference on Machine Learning (pp. 7154-7163). PMLR.* Kyono, T., Zhang, Y., & van der Schaar, M. (2020). CASTLE: regularization via auxiliary causal graph discovery. Advances in Neural Information Processing Systems, 33, 1501-1512.* Luo, Y., Peng, J., & Ma, J. (2020). When causal inference meets deep learning. Nature Machine Intelligence, 2(8), 426-427.* Vowels, M. J., Camgoz, N. C., & Bowden, R. (2021). D'ya like DAGs? A survey on structure learning and causal discovery. ACM Computing Surveys (CSUR).Several deep learning-based methods for DAG learning were proposed in the above papers and they are able to learn nonlinear associations/causalities. The authors can review those papers and discuss whether they are applicable to the problem studied in this work.- Performance evaluation. The proposed method has achieved impressive results compared to the baselines in the evaluation

experiments. Presumably, the major reason was that the proposed method was able to identify nonlinear associations but the two baselines were only able to identify linear associations. I was curious about the possibility of comparing the proposed method to baselines that can identify nonlinear associations (e.g., some methods from the papers listed above).- Reproducibility. In the BMI task, 8 nutrient intakes and bacteria genera that are known to affect BMI were selected to evaluate the methods. Similarly, in the breast cancer task, the top 10 genes with the largest variance from PAM50 genes were selected to evaluate the methods. I would like to see some analyses or discussions regarding the reproducibility of those experiments. For example, would we observe similar results if we switch to another 8 nutrient intakes and bacteria genera for the BMI task or consider the top 20 genes in the breast cancer task?- Ablation analyses. The proposed method relies on the nonlinear deep neural network (DNN) to capture the nonlinear associations. A natural question is how nonlinear or complex the DNN should be to capture the nonlinear associations. For example, how would the results change as a function of the number of layers or hidden units in each layer?Minor points:- Please unify the citation format. For example, on page 15, ref 64 was labeled as [64] but ref 63 was labeled as a superscript.

Level of Interest

Please indicate how interesting you found the manuscript: Choose an item.

Quality of Written English

Please indicate the quality of language in the manuscript: Choose an item.

Declaration of Competing Interests

Please complete a declaration of competing interests, considering the following questions:

- Have you in the past five years received reimbursements, fees, funding, or salary from an organisation that may in any way gain or lose financially from the publication of this manuscript, either now or in the future?
- Do you hold any stocks or shares in an organisation that may in any way gain or lose financially from the publication of this manuscript, either now or in the future?
- Do you hold or are you currently applying for any patents relating to the content of the manuscript?
- Have you received reimbursements, fees, funding, or salary from an organization that holds or has applied for patents relating to the content of the manuscript?
- Do you have any other financial competing interests?
- Do you have any non-financial competing interests in relation to this paper?

If you can answer no to all of the above, write 'I declare that I have no competing interests' below. If your reply is yes to any, please give details below.

I declare that I have no competing interests

I agree to the open peer review policy of the journal. I understand that my name will be included on my report to the authors and, if the manuscript is accepted for publication, my named report including any attachments I upload will be posted on the website along with the authors' responses. I agree for my report to be made available under an Open Access Creative Commons CC-BY license (http://creativecommons.org/licenses/by/4.0/). I understand that any comments which I do not wish to be included in my named report can be included as confidential comments to the editors, which will not be published.

Choose an item.

To further support our reviewers, we have joined with Publons, where you can gain additional credit to further highlight your hard work (see: https://publons.com/journal/530/gigascience). On publication of this paper, your review will be automatically added to Publons, you can then choose whether or not to claim your Publons credit. I understand this statement.

Yes Choose an item.
