# Supplementary material for: Deep neural networks with knockoff features identify nonlinear causal relations and estimate effect sizes in complex biological systems
Source: Gigascience. 2023 Jul 3;12:giad044. doi: 10.1093/gigascience/giad044 (PMC10316696; doi:10.1093/gigascience/giad044)
Supplement: giad044_Reviewer_2_Report_Revision_1 — Yunan Luo -- 2/6/2023 Reviewed [file giad044_reviewer_2_report_revision_1.pdf]

Reviewer Report

Title: Deep neural networks with knockoff features identify nonlinear causal relations and estimate effect sizes in complex biological systems

Version: Revision 1 **Date:** 2/6/2023

Reviewer name: Yunan Luo

Reviewer Comments to Author:

The authors have addressed most of my comments. However, I have two questions below.- Review of related work. The authors have revised their claim as "the first computational method that explicitly learns nonlinear causal relationships". It was not clear to me what "explicitly" mean here. I'd suggest the authors elaborate more and discuss why previous methods were "implicit".- Reproducibility. In response to my question regarding the reproducibility of the proposed method on the BMI dataset, the author mentioned that "we chose to extend our breast cancer data analysis instead of the BMI data analysis since the extension would be more systematic". However, I am not fully sure why it was infeasible (or not systematic) to conduct a reproducibility analysis of results on the BMI dataset. I had this question because it was only mentioned in the manuscript that "we selected thesame number (8) of nutrient intakes and bacteria genera" -- it was unclear why the specific number (8) of nutrient intakes was used. Are there only 8 nutrient intakes that are known to affect BMI, or there are more but only 8 were selected? If it was the latter case, how were the 8 selected (randomly or handpicked)? I think the results would be more convincing if the authors can show the proposed method would still perform the best if we select some number of other nutrient intakes as the positive variables in evaluations. If that is not doable, I would like to see some discussions/justifications why it is infeasible to perform the reproducibility analysis, and also more details about the particular experiment the authors have performed already (e.g., how were the 8 intakes chosen, and why 8, etc.).

Level of Interest

Please indicate how interesting you found the manuscript: Choose an item.

Quality of Written English

Please indicate the quality of language in the manuscript: Choose an item.

Declaration of Competing Interests

Please complete a declaration of competing interests, considering the following questions:

- Have you in the past five years received reimbursements, fees, funding, or salary from an organisation that may in any way gain or lose financially from the publication of this manuscript, either now or in the future?

- Do you hold any stocks or shares in an organisation that may in any way gain or lose financially from the publication of this manuscript, either now or in the future?
- Do you hold or are you currently applying for any patents relating to the content of the manuscript?
- Have you received reimbursements, fees, funding, or salary from an organization that holds or has applied for patents relating to the content of the manuscript?
- Do you have any other financial competing interests?
- Do you have any non-financial competing interests in relation to this paper?

If you can answer no to all of the above, write 'I declare that I have no competing interests' below. If your reply is yes to any, please give details below.

I declare that I have no competing interests

I agree to the open peer review policy of the journal. I understand that my name will be included on my report to the authors and, if the manuscript is accepted for publication, my named report including any attachments I upload will be posted on the website along with the authors' responses. I agree for my report to be made available under an Open Access Creative Commons CC-BY license (http://creativecommons.org/licenses/by/4.0/). I understand that any comments which I do not wish to be included in my named report can be included as confidential comments to the editors, which will not be published.

Choose an item.

To further support our reviewers, we have joined with Publons, where you can gain additional credit to further highlight your hard work (see: https://publons.com/journal/530/gigascience). On publication of this paper, your review will be automatically added to Publons, you can then choose whether or not to claim your Publons credit. I understand this statement.

Yes Choose an item.
